# The Host Adapted Fungal Pathogens of *Pneumocystis* Genus Utilize Genic Regional Centromeres

**DOI:** 10.1101/2023.05.12.540427

**Published:** 2023-05-12

**Authors:** Ousmane H. Cissé, Shelly Curran, H. Diego Folco, Yueqin Liu, Lisa Bishop, Honghui Wang, Elizabeth R. Fischer, A Sally Davis, Spenser Babb-Biernacki, Vinson P. Doyle, Jonathan K. Richards, Sergio A. Hassan, John P. Dekker, Pavel P. Khil, Jason M. Brenchley, Shiv Grewal, Melanie Cushion, Liang Ma, Joseph A. Kovacs

**Affiliations:** 1 Critical Care Medicine Department, Clinical Center, National Institutes of Health, Bethesda, Maryland, USA.; 2 Laboratory of Biochemistry and Molecular Biology, National Cancer Institute, National Institutes of Health, Bethesda, Maryland, USA.; 3 Microscopy Unit, Research Technologies Branch, Rocky Mountain Laboratories, National Institute of Allergy and Infectious Diseases, National Institutes of Health, Hamilton, Montana, USA.; 4 Diagnostic Medicine/Pathobiology, Kansas State University College of Veterinary Medicine, Manhattan, USA.; 5 Museum of Natural Science and Department of Biological Sciences, Louisiana State University, Baton Rouge, Louisiana, USA.; 6 Department of Plant Pathology and Crop Physiology, Lousiana State University AgCenter, Baton Rouge, Louisiana, USA.; 7 Bioinformatics and Computational Biosciences Branch, National Institute of Allergy and Infectious Diseases, National Institutes of Health, Bethesda, Maryland, USA.; 8 Bacterial Pathogenesis and Antimicrobial Resistance Unit, National Institute of Allergy, and Infectious Diseases, National Institutes of Health, Bethesda, Maryland, USA.; 9 Department of Laboratory Medicine, Clinical Center, National Institutes of Health, Bethesda, Maryland, USA.; 10 Laboratory of Viral Diseases, National Institute of Allergy, and Infectious Diseases, National Institutes of Health, Bethesda, Maryland, USA.; 11 Department of Internal Medicine, College of Medicine, University of Cincinnati, Cincinnati, Ohio, USA.

## Abstract

Centromeres are genomic regions that coordinate accurate chromosomal segregation during mitosis and meiosis. Yet, despite their essential function, centromeres evolve rapidly across eukaryotes. Centromeres are often the sites of chromosomal breaks which contribute to genome shuffling and promote speciation by inhibiting gene flow. How centromeres form in strongly host-adapted fungal pathogens has yet to be investigated. Here, we characterized the centromere structures in closely related species of mammalian-specific pathogens of the fungal phylum of Ascomycota. Methods allowing reliable continuous culture of *Pneumocystis* species do not currently exist, precluding genetic manipulation. CENP-A, a variant of histone H3, is the epigenetic marker that defines centromeres in most eukaryotes. Using heterologous complementation, we show that the *Pneumocystis* CENP-A ortholog is functionally equivalent to CENP-A^Cnp1^ of *Schizosaccharomyces pombe*. Using organisms from a short-term *in vitro* culture or infected animal models and ChIP-seq, we identified centromeres in three *Pneumocystis* species that diverged ~100 million years ago. Each species has a unique short regional centromere (< 10kb) flanked by heterochromatin in 16–17 monocentric chromosomes. They span active genes and lack conserved DNA sequence motifs and repeats. CENP-C, a scaffold protein that links the inner centromere to the kinetochore appears dispensable in one species, suggesting a kinetochore rewiring. Despite the loss of DNA methyltransferases, 5-methylcytosine DNA methylation occurs in these species, though not related to centromere function. These features suggest an epigenetic specification of centromere function.

## Short summary

*Pneumocystis* species offer a suitable genetic system to study centromere evolution in pathogens during host adaptation because of their unique specificity for mammals, and their phylogenetic proximity with the nonpathogenic yeast *Schizosaccharomyces pombe*, a popular model for cell biology. We used this system to explore how centromeres have evolved after divergence of the two clades ~460 million years ago. To address this question, we established a protocol combining short-term culture and ChIP-seq to characterize centromeres in multiple *Pneumocystis* species. We show that *Pneumocystis* have short epigenetic centromeres that function differently from those in *S. pombe* and exhibit similarities to centromeres in more distantly related host adapted fungal pathogens.

## Introduction

Centromeres are genomic locations where spindles attach during chromosomal segregation. They are essential for cell division. Errors in the chromosomal segregation can result in aneuploidy and cell division defects with disastrous consequences such as cancer. In fungi, centromeres are fragile sites that are often involved in karyotype variability (Sankaranarayanan, Ianiri et al. 2020, Seidl, Kramer et al. 2020) and drug resistance (Selmecki, Forche et al. 2006). In most eukaryotes, centromeres are defined by the presence of CENP-A nucleosomes, a centromere specific histone H3 variant that is essential for the localization of known kinetochore components (McKinley and Cheeseman 2016). There is a remarkable diversity in centromere structures ranging from sequence-dependent (point) centromeres to epigenetically-regulated (regional) centromeres.

Point centromeres are exemplified by *Saccharomyces cerevisiae* with genetically defined 125 bp long centromeres containing conserved DNA elements (I, II and III) that serve as anchors for the recruitment of the centromeric DNA binding factor 3 (CBF3) complex (Clarke and Carbon 1980, Gordon, Byrne et al. 2011). Regional centromeres are much longer, lack universally conserved DNA patterns, and are often made up of repetitive DNA (Kursel and Malik 2016). Regional centromeres are epigenetically regulated; however, which factors contribute to this epigenetic specification of centromeres remains elusive.

*Pneumocystis* is a genus of pathogenic fungi that exclusively infect mammals and remain unculturable. They belong to the Taphrinomycotina subphylum and form a monophyletic clade with fission yeasts *Schizosaccharomyces*, with a separation time estimated at ~460 million years ago (Shen, Steenwyk et al. 2020, Cisse, Ma et al. 2021). In addition to these two classes there are other classes, all of which are plant or soil adapted organisms. Compared to *S. pombe*, *Pneumocystis* species have streamlined genomes due to substantial gene losses during their transition to animal parasitism. Recently, the genomes of multiple *Pneumocystis* species have been sequenced (Slaven, Meller et al. 2006, Cisse, Pagni et al. 2012, Ma, Chen et al. 2016, Cisse, Ma et al. 2021). However, centromeres were not defined in these because they cannot be predicted bioinformatically. *Pneumocystis* have retained CENP-A, which presumably binds to centromeres, and most of the kinetochore proteins. Some data suggest that *Pneumocystis* centromeres differ structurally from those of *S. pombe*. For example, missing in *Pneumocystis* genomes is the heterochromatin protein Swi6/HP1 (Almeida, Cisse et al. 2015), which is required for replication origins at the centromeres flanking regions (pericentromeres) (Hayashi, Takahashi et al. 2009). The RNAi pathway is lost in *Pneumocystis* (Cisse, Pagni et al. 2014), further supporting mechanistic difference with *S. pombe*. Similar to *S. pombe*, DNA methylation has been predicted to be absent in *Pneumocystis* based on the absence of DNA methyltransferases (DMTs) (Freitag 2017).

In this work, we undertook to characterize *Pneumocystis* centromeres and compare them to *Schizosaccharomyces pombe* centromeres. We found that *Pneumocystis* species have small regional centromeres that are structurally different from those of *S. pombe*.

## Results

### Survey of genes related to centromere function in Taphrinomycotina fungi.

To understand the kinetochore evolution in Taphrinomycotina fungi, we screened the predicted proteomes of 14 species spanning six orders, *Pneumocystis* (*n* = 7), *Schizosaccharomyces* (*n* = 3), *Taphrina* (*n* = 1), *Saitoella* (*n* = 1), *Protomyces* (*n* = 1) and *Neolecta* (*n* = 1), as well as other fungi to have a broad overview of kinetochore evolution.

We searched for homologs of CENP-A ^Cnp1^, CENP-C^Cnp3^, CCAN (Constitutive Centromere Associated Network) and KMN (KNL-1/Mis12 complex/Ndc80 complex) proteins. We extended our searches to pathways required for centromere function and chromosomal segregation (heterochromatin, RNAi, and DNA methylation).

CENP-A, CENP-C, and outer kinetochore proteins are conserved across Taphrinomycotina (Supplementary figure 1). Two genes of the inner kinetochore (CCAN) that are absent in *Pneumocystis* are clearly innovations in *S. pombe* (*fta2* and *fta3* genes). The *fta2*, *fta3* and *fta4* gene products associate with the central core (cnt) and innermost repeats (imr) region of the centromere in *S. pombe* (Liu, McLeod et al. 2005). *Pneumocystis* also lack the *mis17* gene exclusively found in *S. pombe*, which encodes a member of the Mis6-Mal2-Sim4 multiprotein required for CENP-A recruitment in *S. pombe* (Shiroiwa, Hayashi et al. 2011). The *S. pombe* centromere-associated protein B genes (*cbp1*, *cbh1* and *cbh2*), which are derived from the domestication of *pogo*-like transposases (Casola, Hucks et al. 2008) have no identified homologs in other fungi.

The chromosomal passenger complex (CPC) is a heterotetrametric complex composed of the Aurora B kinase Ark1 and three regulatory components Pic1 (inner centromere protein), Bir1p (Survivin) and Nbl1 (Borealin) which mediates chromosome segregation and cytokinesis (Leverson, Huang et al. 2002). This pathway is conserved in *Pneumocystis* and *S. pombe* (Supplementary figure 1).

CENP-A recruiting complex, which includes Mis18 and Mis16 (Hayashi, Fujita et al. 2004) as well as the CENP-A histone chaperone Scm3 required for CENP-A loading at the centromere (Williams, Hayashi et al. 2009) are conserved in *Pneumocystis*, as are the monopolin complex genes that coordinate the kinetochore microtubules attached during mitosis (Corbett, Yip et al. 2010).

The network for heterochromatin formation in *Pneumocystis* seems intact with the presence of the Clr4 methyltransferase complex (Clr4/SUV39H). The only notable loss is Swi6, which with Chp2 are HP1 orthologs, while Chp2 is conserved. DNA methylases (DMTs) are not present in any of the Taphrinomycotina fungi sequenced so far, which suggests that DNA methylation does not occur in this subphylum. Overall, the *Pneumocystis* kinetochore protein catalog is similar to those other Taphrinomycotina fungi except *S. pombe* which seems to be an outlier with a few gene additions. We conclude that *Pneumocystis* species have retained an ancestral version of the kinetochore.

### *Pneumocystis* CENP-A^cnp1^ localizes to the nucleus.

**Figure 1.**
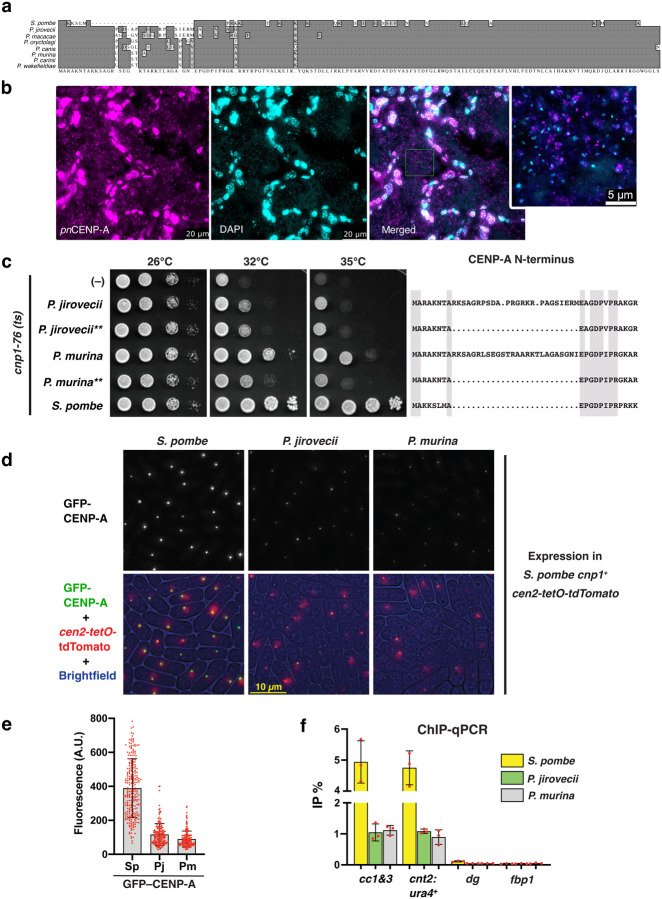
*Pneumocystis* CENP-A variants localize to the nucleus, and support viability and centromere loading in a heterologous system. (**a**) Protein sequence alignment of full-length CENP-A^cnp−1^ homologs in *Schizosaccharomyces pombe* and seven *Pneumocystis* species. Each *Pneumocystis* species only infects a single mammalian host: *P. jirovecii* (infecting humans), *P. macacae* (macaques), *P. oryctolagi* (rabbits), *P. canis* (dogs), *P. murina* (mice), *P. carinii* (rats), and *P. wakefieldiae* (rats). The ~25 amino acid insert in *Pneumocystis* CENP-A N-terminal regions is unique to this genus. (**b**) Immunofluorescence microscopy showing that anti-*Pneumocystis murina* CENP-A (*pn*CENP-A) reacts with *P. murina* organisms. *P. murina*-infected CD40 ligand knockout lung tissue sections were dual labeled using an anti CENP-A antibody(magenta) and 4′,6-diamidino-2-phenylindole (DAPI, light blue). The image is a maximum projection of a 5 μm thick z-stack. The images were minimally adjusted to enhance the contrast. The green box is magnified in the inset showing *pn*CENP-A inside *Pneumocystis* organisms (small dots). *pn*CENP-A signals overlap or are close in proximity with DAPI (represented by a white color produced by the overlap between magenta and light blue colors). (**c**) Left, rescue experiment of fission yeast *cnp1-76* thermosensitive cells using the GFP-tagged CENP-A transgenes. Right, protein sequence alignment of *Pneumocystis* CENP-A N-terminal regions (*P. jirovecii* and *P. murina*) with conserved amino acids highlighted (grey areas). Plasmids containing full length and N-terminal region truncated (**) versions of *Pneumocystis* CENP-A were constructed and integrated as single copy. Growth was assayed at indicated temperatures using 10-fold serial dilutions plated on rich YEA medium. The transgene encoding *S. pombe* CENP-A serves as a non-temperature-sensitive control. (**d**) Representative images of *cen2-tetO-TdTomato* strains expressing the indicated GFP-tagged CENPA transgenes. On the bottom, GFP and tdTomato fluorescence as well as brightfield images are merged. (**e**) Integrated fluorescence intensity of GFP foci was measured and plotted for the strains used in panel e. Mean (bars) and SD (error bars) are shown. n>176. AU, arbitrary units. (**f**) Anti-GFP ChIP-qPCR analysis of indicated loci for strains used in panel e. %IP represents the percentage of input that was immunoprecipitated. Error bars denote the SD (n = 3). Abbreviations: cc1 &3, *S. pombe* centromere central core 1 & 3; cnt2:ura4^+^, ura4^+^ insertion at central core 2; *dg*, a class of heterochromatic outer repeats within pericentromeric regions (control); *fbp1*, euchromatic locus (control).

Centromeres are identified by tracking CENP-A binding regions. There are currently seven *Pneumocystis* species with fully sequenced genomes (Ma, Chen et al. 2016, Cisse, Ma et al. 2021). All of them encode an ortholog of *S. pombe* CENP-A^cnp1,^ (Figure 1a). The CENP-A histones from these species, henceforth referring to as *pn*CENP-A, display a high overall conservation (89% average protein identity) with most divergence in the N-terminal tail.

Precise nuclear localization of CENP-A is required for accurate chromosomal segregation. To determine if *pn*CENP-A localizes in the nucleus, we generated specific antibodies using synthetic immunogenetic peptides representing sequences located in the N-terminus of the protein, to minimize cross reactivity with host histones (Supplementary figure 2; Supplementary methods). We used anti – *Pneumocystis* CENP-A antibodies in conjunction with DAPI to demonstrate that *pn*CENP-A colocalizes with DAPI which support a nuclear localization within the *Pneumocystis* nucleus (Figure 1b).

### *pn*CENP-A variants are functional in *S. pombe*, and support viability and centromere loading.

To confirm that the putative *cenp-a* genes from *Pneumocystis* encode functional CENP-A proteins, we expressed the *cenp-a* genes from *P. jirovecii* and *P. murina* in the related fission yeast *S. pombe*, a tractable organism of the subdivision Taphrinomycotina widely used as model for chromosome and cell biology. The transgenes were codon-optimized, expressed under the endogenous *S. pombe cnp1*, the CENP-A ortholog, gene promoter and inserted in single copy at the *lys1* locus. In addition, the gene products were tagged with GFP at their N-termini since it has been shown that C-terminal tagging (*i.e*., *cnp1-GFP*) displays growth retardation (Lando, Endesfelder et al. 2012). As *Pneumocystis* CENP-A proteins harbor an intervening sequence at the N-terminal tail that may potentially interfere with function in *S. pombe*, we also expressed truncated versions missing ~25 aa from the N-terminal tail (Figure 1c). We first assessed the ability of *Pneumocystis* CENP-A to rescue lethality of *cnp1-76* cells, a thermosensitive mutant, whose mutation T74M lies at the CATD (CENP-A targeting domain) region (Castillo, Mellone et al. 2007). As previously reported (Folco, Campbell et al. 2015), we found that the control transgene (SpCENP-A) restored growth of *cnp1-76* cells at restrictive temperatures (i.e., 32°C and 35°C). Importantly, *Pneumocystis* counterparts were also able to rescue the thermosensitive phenotype, albeit partially (Figure 1c), nevertheless proving the functionality of these genes. Results were similar between constructs harboring either intact or truncated N-termini (Figure 1c). Therefore, we continued our analyses with the full-length proteins. It is well established that in interphase, *S. pombe* displays the Rabl configuration, in which centromeres cluster to the spindle pole body while telomeres associate with each other near nuclear periphery (Funabiki, Hagan et al. 1993, Mizuguchi, Fudenberg et al. 2014). As expected, expressing *S. pombe* CENP-A tagged with GFP resulted in cells displaying single fluorescence foci that colocalized with a *tetO* array inserted at *cen2* (NB: a TetR-tdTomato fusion is expressed to bind to the *tetO* array and this locus is referred to as *cen2-tetO-tdTomato*) (Figure 1d). Consistent with the mentioned rescue of *cnp1-ts* cells and further supporting their role as functional CENP-A proteins, *Pneumocystis* CENP-A proteins also colocalize with *cen2-tetO-tdTomato* and remarkably, they exhibited single foci, although the intensities of centromere foci were dimmer than that of the *S. pombe* counterpart (Figure 1e). Finally, to confirm the specific localization of *Pneumocystis* CENP-A proteins to *S. pombe* centromeres, we performed ChIP-qPCR (Figure 1f). Noteworthily, we observed that *Pneumocystis* CENP-A localized to central core regions (i.e., *cc1&3, cnt2:ura4*^*+*^) similarly to the *S. pombe* counterpart, albeit the enrichment was lower, consistent with the results from live-cell imaging. Additionally, *Pneumocystis* CENP-A localization seems to be specific as no detectable enrichment was found at pericentromeric outer repeats coated with heterochromatin (i.e., *dg*) and euchromatin locations (i.e., *fbp1*). Together, these results confirm that *Pneumocystis* CENP-A genes encode bona fide CENP-A proteins.

### *pn*CENP-A binds to single genomic foci in *Pneumocystis* replicating cells.

**Figure 2.**
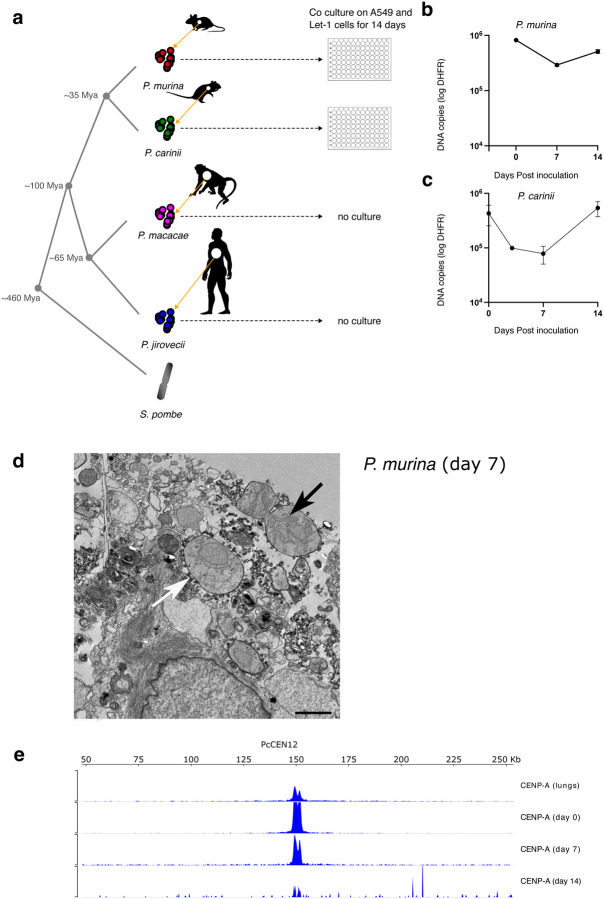
*Pneumocystis* CENP-A binds to single genomic foci in replicating cells **a**) Study workflow and sample preparation. Four *Pneumocystis* species infecting mice, rats, macaques, and humans respectively were analyzed. *P. murina* and *P. carinii* were obtained from CD40 ligand knock-out female mice and immunosuppressed Sprague-Dawley male rats, respectively, and cultured on a co-culture of human lung adenocarcinoma cells (A549) and immortalized murine lung epithelial type 1 cells (Let-1) for 14 days. *P. macacae* organisms were obtained from a Simian Immunodeficiency Virus infected macaque (P2C), and *P. jirovecii* organisms were obtained from a single bronchoalveolar lavage from a non-infected HIV patient and three autopsy samples from patients with HIV infection. Species phylogeny and estimated speciation timing are presented. Animal icons were obtained from http://phylopic.org under creative commons licenses https://creativecommons.org/licenses/by/3.0/: mouse (Anthony Caravaggi; license CC BY-NC-SA 3.0); and rat (by Rebecca Groom; license CC BY-NC-SA 3.0). **b**) *P. murina* population growth in duplicate wells were measured by quantitative PCR targeting a single copy dihydrofolate reductase (*dhfr*) gene over 14 days. Error bars represent the standard deviation (*n*=2). **c**) *P. carinii* growth measurement by qPCR targeting *dhfr* gene. Error bars represent the standard deviation (*n*=2). **d**) Electron micrograph showing a possibly dividing *P. murina* trophic form seven days post culture (black arrow). The field also include a non-dividing trophozoite (white arrow). 2 μm scale bar. **e**) *pn*CENP-A ChIP-seq study in *P. carinii* organisms from infected lungs and cultures at days 0, 7 and 14. CENP-A remains strongly enriched at a single genomic location up to 7 days. Only one representative chromosome is presented. The remaining 16 chromosomes have similar ChIP-seq profiles and presented in supplementary figure 4.

In *S. pombe*, CENP-A^cnp1^ is deposited at the centromeres during the G2 phase of the cell cycle (Lando, Endesfelder et al. 2012). Although *Pneumocystis* cell replication is not fully understood, these organisms take 5 to 8 days to replicate *in vivo* (Vestereng, Bishop et al. 2004). To determine *pn*CENP-A bound genome regions, we established a short-term co-culture system for *P. murina* and *P. carinii* (Figure 2a). We used quantitative PCR targeting the single copy gene dihydrofolate reductase (*dhfr*) to assess organism growth; a typical growth curve shows a decline from day 0 to 7 followed by a gradual increase (Figures 2b and 2c). Population growth is further supported by the presence of mitotic or fusing cells 7 days post culture, though some cells do not appear to be dividing (Figure 2d). We assessed *Pn*CENP-A location on the *P. carinii* genome using ChIP-seq targeting chromatin prepared from infected lung tissue lysates and cultured organisms harvested at three time points. CENP-A binds to discrete areas, which is consistent with centromeres (Figure 2e and Supplementary figure 3).

We mapped centromeres in all *P. carinii* and *P. murina* chromosomes by ChIP-seq using anti*pn*CENP-A and anti-*pn*CENP-C antibodies (Supplementary figures 2a and b) with cell cultures collected from three timepoints: days 0 (normalized initial inoculum), 7 and 14; pre-immune sera served as negative controls (Input). Five independent experiments were performed for each species. We removed host DNA contamination from raw sequence reads by mapping against the rat and mouse genomes; filtered reads (0.3–5% of total reads) were aligned to the *Pneumocystis* genomes. We the genome-wide ratios of immunoprecipitated DNA sequences and matching controls (input sequences) to identify significantly enriched genomic loci using a false discovery rate [FDR] < 0.05 and a minimum fold enrichment of 1.2. Enriched regions following immunoprecipitation (IP) were inspected using IGV genome browser. DeepTools fingerprint plots show that most reads are mapped within a small fraction of the genome (~3% of the total 7–8 Mb), which indicates that the antibodies demonstrated a restricted binding (Supplementary figure 2c). Using ChIP-qPCR, we confirmed *pn*CENP-A enrichment over regions flagged by ChIP-seq compared to a control region taken 200 kilobases away from centromere 1 of each species) for 16 of 17 *P. murina* chromosomes and all 17 *P. carinii* chromosomes (Supplementary figure 4).

**Figure 3.**
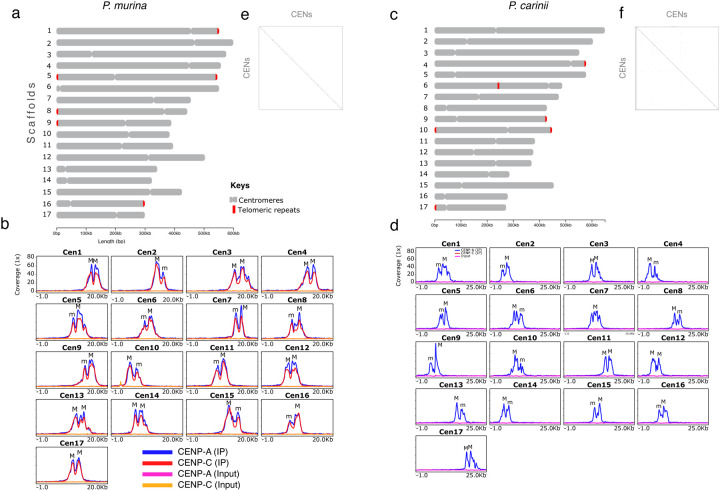
*Pneumocystis* displays seventeen centromeres. **a**) Scaled ideogram of 17 chromosomal level scaffolds (grey) of *P. murina* genome showing CENP-A binding regions (constricted areas) and telomeric repeat motif (red). **b**) CENP-A and CENP-C binding regions delineate putative centromeres (CENs) in *P. murina* genome. Color coded peaks represent enrichment of immunoprecipitated DNA (IP DNA) relative to controls (Input DNA). The 1-kb flanking sequences from the center of the enrichment peak are presented. Each scaffold displays two peaks labelled M (Major) and m (minor) according to the enrichment level. **c**) Scaled ideogram of 17 chromosomal level scaffolds (grey) of *P. carinii* genome showing CENP-A binding regions (constricted areas) and telomeric repeat motif (red). **d**) CENP-A binding regions delineate putative centromeres (CENs) in *P. carinii* genome. Note that no peak was observed with anti-CENP-C antibody. **e**) A DNA dot plot of CENP-A binding regions in *P. murina* showing regional self-similarity. The main diagonal represents the sequence alignment with itself. Lines off the main diagonal which are repetitive patterns within the sequences are not observed. The plot shows that each CEN is unique and repeat-free. **f**) DNA dot plot of CENP-A binding regions in *P. carinii* showing that each CEN is unique and repeat-free.

In *P. murina*, each of the 17 chromosome-level scaffolds display a single region with overlapping peaks of CENP-A and CENP-C (Figures 3a and 3b). The *P. carinii* genome also displays monocentric chromosomes enriched with CENP-A but they lack any CENP-C enrichment (Figures 3c and 3d).

**Figure 4.**
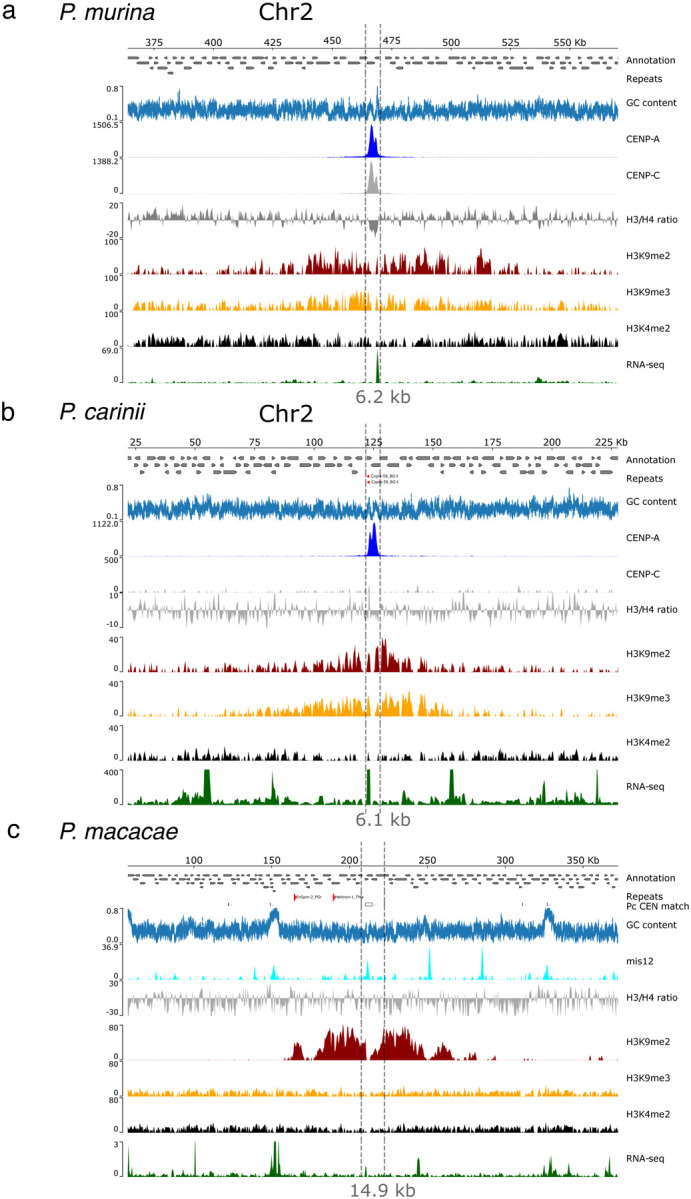
Centromeres are flanked by heterochromatin and contain active genes. All three scaffolds are homologous. **a**) Genomic view of chromosome 2 of *P. murina* genome subsequently showing annotated genes (directed grey boxes), DNA repeats (not present), percent GC content (blue), ChIP-seq read coverage distribution (BPM normalized over bins of 50 bp; input subtracted) of CENP-A, CENP-C, histones H3 and H4 ratio, heterochromatin-associated modifications (H3K9me2 and H3K9me3), euchromatin (H3K4me2) and gene expression (RNA-seq) in relation with centromeres. Genome views of remaining 16 chromosomes are presented in Supplementary dataset figure 7. **b**) Genomic view of chromosome 2 of *P. carinii* genome with the same features presented as *P. murina*. A duplicated copy of *copia*-retrotransposon is presented (red arrows), which is present in syntenic regions in other *Pneumocystis* genomes. Genome views of all remaining 16 chromosomes are presented in supplementary figure 6. **c**) Genomic view of chromosome 14 of *P. macacae* genome showing the distribution of gene density, repeats (DNA transposons), homology region with GC content, mis12 ChIP-seq read coverage, chromatin-associated modifications (H3K9me2 and H3K9me3 and H3K4me2) and RNA-seq in relation with centromeres. The white box indicates a DNA sequence homologous to *P. carinii* centromere 2. A single copy of the DNA transposon EnSPM (CACTA family) and the class 2DNA transposon Helitron are present upstream the centromere (forward red arrows). These elements are not present in syntenic regions in other *Pneumocystis* genomes. Genome views of all remaining 15 chromosomes are presented in Supplementary figure 5.

Enriched regions in both species span 4.8 to 8.0 kb, with an average length of 6.7 kb, and have an average of 3.9% lower GC content than the rest of the genome (Figures 4a and 4b; Supplementary Table 1). *P. murina* and *P. carinii* centromeres encode 74 and 58 genes respectively; all of them are conserved in the genomes of both species except a prefoldin gene that is lost in *P. carinii* (Supplementary Table 2). Of the 74 *P. murina* centromeric genes, 73 were expressed based on RNA-seq and four were further detected by protein mass spectrometry mapping (LC-MS); of the 58 *P. carinii* genes, 56 are expressed and 53 were detected by LC-MS. Analysis of the predicted function of these genes revealed housekeeping functions without major differences compared to randomly sampled genes.

To map *P. macacae* centromeres, we performed ChIP-seq on infected lung tissues from two Simian immunodeficiency virus (SIV)-infected macaques with untreated *Pneumocystis* pneumonia (cf. Supplementary Methods). We prepared chromatin directly from lung tissues without culture. Because anti-CENP-A and anti-CENP-C antibodies specific to *P. macacae* (Supplementary figures 2a and 2b) yielded nonspecific peaks in ChIP-seq experiments, we utilized two antibodies targeting *P. macacae* Mis12 (Supplementary figure 2d), a member of the outer kinetochore that has been used as replacement of CENP-A for centromeres identification in the fungal pathogen *Mucor circinelloides* (Navarro-Mendoza, Perez-Arques et al. 2019). Mis12 ChIP-seq identified −4–6 peaks per chromosome (Figure 4c; Supplementary figure 5). Mis12 peaks tend to occur in GC rich regions. It is unclear if the multiple peaks correspond to ChIP-seq artifacts or if Mis12 truly binds to multiple locations per chromosome. However, there is only one peak flanked by heterochromatin (H3K9me2) per chromosome, which also flanked *P. carinii* and *P. murina* centromeres (Figures 4a and 4b, Supplementary figures 6 and 7). DNA sequence similarity searches showed that this peak is homologous to *P. carinii* and *P. murina* centromeres. We consider this peak to be a putative centromere.

We were unable to map centromeres in *P. jirovecii* despite utilizing anti-CENP-A and anti-CENP-C antibodies specific for this species (inconclusive ChIP-seq results), possibly because the only samples available for Chip-seq studies were from patients previously treated with anti-*Pneumocystis* drugs.

### Centromeric nucleosomes assemble differently among species.

CENP-C binds to specific motifs in CENP-A in the C-terminal cupin domain involved in dimerization (Kato, Jiang et al. 2013). To confirm the absence of CENP-C ChIP-seq peaks at *P. carinii* centromeres, we analyzed chromatin by co-immunoprecipitation (Co-IP) with anti-CENP-A, anti-CENP-C and anti-H4 antibodies followed by LC-MS analysis. While CENP-A was detected by peptide mapping with high confidence (peptide: “WQSTAILCLQEATEAFLVHLFEDTNLCAIHAK”, Qvality q-value = 0.00553986), none of the CENP-C peptides were identified in either of the two runs. Of note, we detected CENP-C in total proteins from both species by LC-MS and Western blotting (Supplementary figures 2e and f). These results are consistent with ChIP-seq results and suggest that *P. carinii* CENP-C does not localize at the *P. carinii* centromeres. *pn*CENP-C may be dispensable in *P. carinii*, similar to *S. pombe* (Sridhar and Fukagawa 2022).

**Figure 5.**
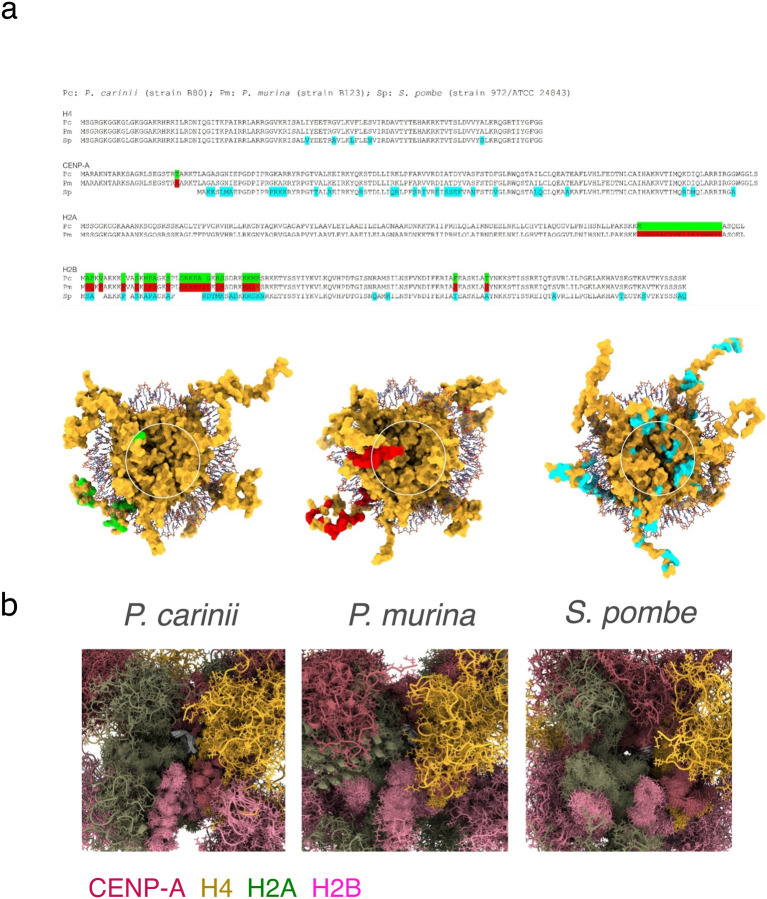
Modeling of Molecular dynamics of CENP-A nucleosomes in *P*. carinii, *P. murina* and *S. pombe*. **a**) Upper panel: Sequence alignment of the centromeric histones used in modeling and simulations. Differences between *P. carinii* and *P. murina* colored green and red; differences between *S. pombe* and *pneumocystis* colored blue. Lower panel: surface representation of the centromeric octamers of *P. carinii* (left), *P. murina* (middle), and *S. pombe* (right), showing the spatial location of the mutations (snapshots from the simulations); DNA (not simulated) shown only as a reference based on the crystal structure of the human centromere nucleosome, PDB: 3AN2). The white circles show the central crevices, the behaviors of which are expected to dictate the assembly of the inner kinetochore in each species. **b**) Aggregation of snapshots along the dynamic trajectory illustrating the extent of species-specific conformational variations of the central crevices (see Animation in Supplementary dataset 2); histones color code is presented at the bottom). The C-terminal tip of CENP-A is located at the center of the core (white ribbon; residues 146–151 in *Pneumocystis* and 120 in *S. pombe*; see alignment in panel a). The observed differences between *P. carinii* and *P. murina* are due to structural and dynamic changes induced by the different (H2A:H2B) dimers (cf. alignment in panel a); the differences between these species and *S. pombe* are likely the result of more extensive sequence variations in the four histones, including several solvent-exposed mutations at the core center (cf. panel a). Early-stage assembly of the inner kinetochore is thus expected to be strongly determined by features of this crevice, including its size, dynamics, and other physicochemical features.

To gain further molecular insight into the CENP-A nucleosome differences, we performed molecular dynamics (MD) simulations of centromeric complex models of *P. carinii*, *P, murina*, and *S. pombe* (cf. Supplementary Methods). The analysis suggests that the observed differences can be explained by the different histones H2A:H2B dimers recruited by each species at the centromeres (Figure 5a). Comparative analysis shows that variation in the terminal regions of these proteins can induce structural and dynamic changes in the region surrounding the central crevice formed by the spatial confluence of four histones (cf. Figure 5b and Supplementary animation D2). This region of the octameric complex is solvent-exposed and poised for binding to inner-kinetochore proteins such as CENP-C. Slight variations in its average size or dynamics can affect protein selection and binding process, hence the early-stage assembly mechanism, even when its amino acid composition is identical, as is the case of *P. carinii* and *P. murina*. Compared to these species, *S. pombe* presents several mutations lining the crevice (cf. Figure 5a), which adds physicochemical features to consider when comparing the two organisms. For the *Pneumocystis* sequences modeled here, the structural and dynamic differences between the two species stem from the longer C-terminal domain of H2A in *P. murina*, which adopts a helical conformation with several nonpolar moieties. This helix is quite mobile and tend to interact hydrophobically with the disordered N-terminus of CENP-A (Supplementary Table 3). However, when free from these interactions, the helix can transiently gravitate towards the core center, affecting the exposure of the crevice to the solvent, hence to other proteins (cf. Figure 5b). Transient helices in intrinsically disordered segments can have functional implications (Chen, Yang et al. 2021, Zhao, Nguyen et al. 2022), and hydrophobic helixes, in particular, can play a role in molecular recognition and drive protein association and aggregation (Zhao, Nguyen et al. 2022).

### *Pneumocystis* CENP-A replaces the canonical histone H3 at the centromeres.

In *S. pombe*, CENP-A^cnp1^ enrichment at the centromeres correlates with a depletion of histone H3 (Thakur, Talbert et al. 2015). To determine if this feature is present in *Pneumocystis*, we quantified the relative abundance of canonical histones H3 and H4 using ChIP-seq. We examined the 16–17 centromeres per species, their flanking regions (30 kb) and control regions selected 25 kb away from centromeres. H3 occupancy (expressed as ratio between H3 and H4) was found to be significantly reduced at centromeres relative to controls in *P. carinii* and *P. murina* but not *P. macacae* (Wilcoxon test; *P* < .0001; Supplementary figure 8). In parallel to ChIP-seq, we found by ChIP-qPCR a reduction of histone H3 in 15 of the 17 *P. carinii* centromeres, and in 2 of the 17 *P. murina* centromeres compared to the controls (Supplementary figure 9). The discrepancy between *P. murina* ChIP-seq and ChIP-qPCR is potentially due to variability in culture experiments.

### Centromeres sequences are unique and lack repetitive sequences.

Centromeres are located in non-repetitive sequences within the *Pneumocystis* genomes (Figures 3e and 3f). Motif searches also did not reveal any conserved motif either in the centromeric regions as a whole or when focused on *pn*CENP-A peaks. To determine if repeats (DNA transposons and retrotransposons) are associated to centromeres, we searched for known signatures of repeats. In *P. murina*, we detected long terminal repeats (LTRs) (*copia* and *gypsy*) in the vicinity of 5 of the 17 centromeres but they are not directly flanking the CENs (Supplementary figure 7). In *P. carinii*, of the 17 centromeres, the CEN2 contains a duplicated copy of *copia*-retrotransposons and CEN16 has a *gypsy* repeat in its upstream region (Figure 4b; Supplementary figure 6). The repeated DNA elements are likely nonfunctional for further transposition because they lack essential domains required for transposition activity (LTRs, reverse transcriptase, integrase) and they are integrated into genes. No repeat is present in the vicinity of *P. macacae* centromeres (Supplementary figure 5).

### Centromeres are flanked by heterochromatin and lack DNA methylation marks.

In *S. pombe*, H3K9me2 marking of chromatin is associated with the repression of centromeres, subtelomeres, ribosomal rDNA and the mating locus (Cam, Sugiyama et al. 2005). There are two types of chromatins: euchromatin, the lightly packed form of the chromatin enriched with H3K4me2 (di-methylation of the 4^th^ lysine residue of the histone H3), which is associated with active transcription, and heterochromatin enriched with H3K9me2 and H3K9me3 (di and tri-methylation of the 9^th^ lysine residue of the histone H3 protein). Heterochromatin regulates mating and sporulation gene expression in *S. pombe*, in addition to controlling mating-type switching (Allshire and Ekwall 2015). To test if this feature is shared in *Pneumocystis*, we performed ChIP-seq with antibodies targeting histones H3 modifications (H3K9me2/3 and H3K4me2). In *Pneumocystis*, peaks of H3K9me2 and H3K9me3 are only present at the pericentromeres (Supplementary figures 5, 6 and 7). In all three species analyzed (*P. carinii*, *P. murina* and *P. macacae)*, H3K9me2 enrichment is significantly more pronounced in pericentromeric regions compared to centromeres (Figure 4; Wilcoxon test, *P* < .00001; Supplementary figure 8). H3K9me3 enrichment at pericentromeric regions compared to centromeres is only significant in *P. carinii*. We observed a reduced transcriptional activity by RNA-seq analysis only in *P. carinii* pericentromeres (Supplementary figure 8). H3K4me2 is correlated with H3K9me2 in the centromeres (Pearson rho = .72) but not at the whole genome level (r = 0.05). However, research has shown that the histone modifications H3K4me2 and H3K9me2 decorate heterochromatin and euchromatin regions, respectively (Yasuhara and Wakimoto 2008). Therefore, the correlation observed between H3K4me2 and H3K9me2 may be due to the *Pneumocystis* population of cells used in the ChIP analysis. Furthermore, similar to *S. pombe* (Cam, Sugiyama et al. 2005), a small amount of H3K4me2 is present at the CENP-A containing of centromeres in *Pneumocystis* (Supplementary figure 8). These results suggest that *Pneumocystis* centromeres are flanked by heterochromatin.

DNA methylation is frequently associated with centromeres in fungi, where they silence repeats (Miura, Yonebayashi et al. 2001, Smith, Galazka et al. 2012). To determine whether DNA methylation plays a role in *Pneumocystis* centromeres, we performed bisulfite sequencing (5 methylcytosine) in *P. carinii* and *P. macacae*. The overall level of 5mC DNA methylation as measured by the average weighted methylation percentage is 0.6% for *P. carinii* and 2% for *P. macacae* at the CG dinucleotides (Supplementary figure 10). These levels are in range with reported levels for other fungi e.g., *Verticillium* (0.4%) (Cook, Kramer et al. 2020) and *Neurospora* (2.5%) (Hosseini, Meunier et al. 2020). To assess the potential role of DNA methylation implication in centromere function, we analyzed the DNA methylation patterns over different genomic features (genes, intergenic spacers, and centromeres). DNA methylation levels are higher in genes compared to centromeres (Mann-Whitney U-test *P* = .005; Supplementary figure 10). However, there is no significant difference among centromeric or pericentromeric regions (defined as 30 kb flanking the centromeres: P-value > 0.3), and the randomly selected genomic regions (genomic background). These results suggest that 5-mC DNA methylation is not required for centromere function in *Pneumocystis*, consistent with the absence of repeats in the centromeres.

### Centromere conservation during speciation

**Figure 6.**
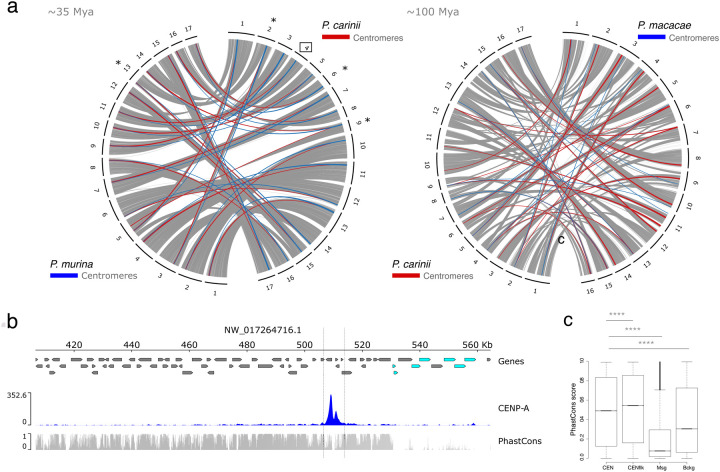
Centromere locations, not sequences, are evolutionary constrained by negative selection. **a**) Genome organization and synteny in *Pneumocystis*. Circos plots depicting pairwise *P. carinii*, *P. murina* and *P. macacae* genome synteny. Rodent infecting Pneumocystis (*P. carinii* and *P. murina*) and macaques infecting *Pneumocystis* (*P. macacae*) have 17 and 16 chromosomal level scaffolds, respectively. Colored connectors indicate regions of synteny between species. Centromeres that overlap with recent chromosomal breakpoints are indicated (*). The square highlights *P. carinii* centromere 4 displayed in the panel b. **b**) Genome view of centromere 4 in the *P. carinii* genome. Genes are represented by directed boxes (grey for protein coding genes and cyan for polymorphic major surface glycoprotein genes), *pn*CENP-A binding region (centromere) and sequence conservation scores which were calculated from whole genome alignments of *P. carinii*. *P. murina* and *P. wakefieldiae* (PhasCons). The phastCons scores represent probabilities of negative selection and range between 0 (no conservation) and 1 (total conservation). **c**) Boxplot of conservation scores per genomic context summarized over all *P. carinii* centromeres (CEN), 30-kb regions flanking the centromeres (Cenflk), major surface glycoproteins (Msg) and random genomic background (Bckg). Statistical differences for the indicated comparisons were obtained using one-sided nonparametric Mann-Whitney test; *P*-values < .0001, ****; *P* < .001, ***; *P* < .01, **; *P* < .05, *; ns, not significant.

*Pneumocystis* species diverged about 100 Mya (Figure 2a). Yet, homologous regions act as centromeres in *P. carinii*, *P. murina* and *P. macacae* (Figure 6a, Supplementary table 4). Centromeres in these three species have similar DNA sequences and in *P. jirovecii* as well as *P. wakefieldiae*, *P. oryctolagi* and *P. canis* (Supplementary figure 11), which indicates that sequences are vertically transmitted.

To investigate footprints of selection acting on centromeres, we computed conservation scores (PhastCons) from whole genome alignments. PhastCons conservation scores range from 0 to 1 and represent probabilities of negative selection. Centromeric regions are more conserved than genomic background (Wilcoxon test *P* < 2.2e-16; Figures 6b and 6c). This suggests that centromeres are maintained by negative selection. This is consistent with the hypothesis that centromere positioning tends to be conserved in obligate sexual fungi due to their role in meiosis (Schotanus, Soyer et al. 2015).

Centromeres may contribute to karyotypic diversity in fungi. However, we found little support for this hypothesis here because most centromeres do not overlap with chromosomal breaks (only 3 of 17 in the *P. carinii* versus *P. murina* pairwise comparison) (Figure 6a).

## Discussion

**Figure 7.**
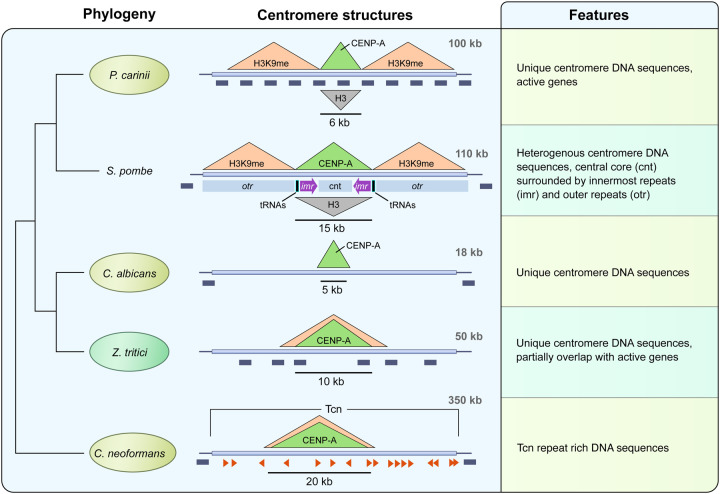
Model of regional centromere structures in *Pneumocystis* and other representative fungi. On the left is presented the phylogeny of selected fungi inferred from maximum likelihood phylogenetic analysis of shared core protein orthologs. The overall centromere structures for *Pneumocystis carinii* (details supporting our model are provided in the text), *Schizosaccharomyces pombe* (Pidoux and Allshire 2004), *Candida albicans* (Sanyal, Baum et al. 2004), *Zymoseptoria tritici* (Schotanus, Soyer et al. 2015) and *Cryptococcus neoformans* (Janbon, Ormerod et al. 2014) are used as representative to showcase the diversity of regional centromeres in fungi. Animal pathogens are highlighted in pale olive and the plant fungal pathogen *Zymoseptoria tritici* is in pale green. For sake of brevity, only *Pneumocystis carinii* is presented for brevity, although there are differences with other *Pneumocystis* species. In the middle are presented DNA structures of centromeres. *P. carinii* has 17 centromeres (one for each of its 17 chromosomes) that share the same overall architecture. Centromeres are delineated by a localized enrichment of the centromeric histone CENP-A, which overlap with a reduction of the canonical histone H3 (inverted grey triangle). Centromeres are flanked by heterochromatin H3K9me (stands here stands for both H3K9me2 and H3K9me3). All 17 *Pneumocystis carinii* centromeres span active genes (dark grey boxes). Each centromere sequence is different and lacks shared DNA sequence motif. *S. pombe* has three centromeres that share the same overall structure in which a central core (*cnt*) domain is surrounded by innermost repeats (*imr*) and outer repeats (*otr*). The *imr* repeats incorporate clusters of transfer RNAs (tRNAs) that play a role in restricting CENP-A spread. *S. pombe* centromeres are flanked by heterochromatin (H3K9me). Genes are found 0.75–1.5 kb beyond the limits of the centromeres. *C. albicans* has 8 unique and different centromeres that are gene free and lack shared sequence motifs. *Z. tritici* has 21 centromeres ranging from 6 to 14 kb in size, that partially overlap with genes. *C. neoformans* has 14 centromeres that are gene free and enriched with Tcn transposons.

In the current study we have identified putative centromeres of multiple *Pneumocystis* species and demonstrated that they are short regional centromeres with pericentromeric heterochromatin and span active genes (Figure 7). Cell replication and chromosomal segregation pathways are unexplored in these species. Central to these pathways are the centromeres.

As centromeres cannot be predicted bioinformatically, the locations and characteristics of *Pneumocystis* centromeres were unknown. Moreover, there are several roadblocks for characterizing centromeres in *Pneumocystis*, with the most significant one being the lack of continuous culture and transfection tools. Here we utilized two species of organisms in a short-term culture system to determine where *pn*CENP-A, the epigenetic marker for centromeres, binds in the genome. We demonstrated that *Pn*CENP-A functions similarly to CENP-A^*Cnp1*^ in *S. pombe*, though less efficiently, validating its use in characterizing *Pneumocystis* centromeres. Unlike the phylogenetically related *S. pombe*, in *Pneumocystis* these regions overlap with active genes, but like *S. pombe*, these centromeres are flanked by heterochromatin. Our work provides the first experimental evidence that DNA methylation occurs in these species, although it may not be involved in centromere function. Taken together, our results suggest that *Pneumocystis* has small epigenetically regulated centromeres. Individual centromeres are conserved among different species, though not across chromosomes within species.

Differences between *P. carinii* and other species analyzed here (*P. murina* and *P. macacae*) need to be clarified. *P. carinii* and *P. murina* centromeres are homologous, yet there seems to be heterochromatin silencing only in *P. carinii*. Also, CENP-C is not linked to CENP-A or centromeres in *P. carinii*, which may instead be using another histone linker (e.g., CENP-T) to connect the centromere to the kinetochore (Sridhar and Fukagawa 2022).

The presence of genes within centromeres is rare and has only been described in rice (Nagaki, Cheng et al. 2004) and the plant fungal pathogen *Zymoseptoria tritici* (Schotanus, Soyer et al. 2015). Centromeres bearing genes are interpreted as young centromeres (neocentromeres), in which the genes are progressively inactivated. This hypothesis lacks support here because there is no sign of pseudogenization in these genes: nearly all are expressed and translated, they are conserved in all species, and many are involved in housekeeping cellular pathways that are presumably critical for organism survival.

Findings in *Pneumocystis* cannot be generalized to other Taphrinomycotina species. The determination of ancestral traits between *Pneumocystis* and fission yeast centromeres will require characterizing the centromeres in additional Taphrinomycotina species. The loss of RNAi is often associated with shortening of centromeres (Yadav, Sun et al. 2018); consistent with this, *Pneumocystis* centromeres are much smaller than *Schizosaccharomyces* centromeres.

Our study will benefit from a microscopy validation of CENP-A loading kinetics when a reliable long term culture system becomes available. Live imaging would help to determine at which cell cycle stage CENP-A is loaded.

In summary, we have identified atypical short regional centromeres in genetically intractable micro-organisms. Our results provide insights into the formation of centromeres in host-adapted fungal pathogens. Our ultimate goal is to use *Pneumocystis* centromeres to stabilize plasmids for future genetic manipulation. This is the first step along this path, which should lead to better understanding of the biology of *Pneumocystis* and facilitate the discovery of novel interventions to effectively control and prevent the disease caused by this pathogen.

## MATERIAL AND METHODS

### Ethics and organism source

Studies involving human and mouse samples were approved by the NIH Institutional Review Board (IRB) (IRB protocols 99-I-0084 and CCM 19–05, respectively), rat *Pneumocystis* studies by the Animal Care and Use Committees of the Cincinnati VA Medical Center (protocol 20-11-08-01) and macaque *Pneumocystis* studies by the National Institute of Allergy and Infectious Diseases (NIAID) Division of Intramural Research (protocol LVD 26).

*P. carinii* organisms were collected from heavily infected lungs of corticosteroid-treated Sprague-Dawley male rats; *P. murina* from heavily infected lungs of CD40 ligand knock out mouse; *P. macacae* from heavily infected lungs of two Simian Immunodeficiency Virus (SIV) infected macaques (P2C and P3C); and *P. jirovecii* organisms were isolated from a single bronchoalveolar lavage from one patient and 3 heavily infected autopsy lung tissues from three different patients. The list of samples is presented in the key resource file.

### *Pneumocystis* culture and growth quantification

*P. murina* and *P. carinii* were partially purified by Ficoll-Hypaque density gradient centrifugation (Kovacs, Halpern et al. 1988) and frozen at −80°C in cell recovery media (Gibco). For short term *Pneumocystis* cultures, A549 (ATCC) and LET1 (a gift from Dr. Paul Thomas, St Jude Children’s Research Hospital) cells were cultured in culture medium F12 media with 2.5% or 5% heat inactivated fetal bovine serum (GIBCO) and Penicillin-Streptomycin (GIBCO), plated to approximately 60 – 80% confluency and incubated for 24h at 37°C. The next day, frozen *Pneumocystis* vials were thawed, washed in 50ml 1x PBS, and centrifuged at 2,000g for 20 minutes. *Pneumocystis* cell pellets were resuspended in culture medium and added to the plated cells. Media were partially changed every 3 or 4 days. At set time points, wells were scraped for collection. Cell/organism suspensions were centrifuged at 10,000g for 3 min, the supernatant was removed, and the pellets were frozen at −80°C. Genomic DNA was extracted using QiAmp DNA Extraction Kit (Qiagen). Quantitative PCR targeting the single copy *dhfr* gene was performed as described previously (Liu, Davis et al. 2020).

### *Pn*CENP-A complementation and ChIP-qPCR in *S. pombe*

Standard procedures were used for fission yeast growth, genetics, and manipulations (Sabatinos and Forsburg 2010). *P. jirovecii* and *P. murina* CENP-A full-length and N-terminal truncated DNA sequences were codon optimized for *S. pombe* codon bias (Supplementary dataset 1), synthesized commercially (GenScript, NJ) and subcloned into pS2 vector (Takayama, Sato et al. 2008). Recombinant plasmids were isolated from *E. coli* strain *DH5α* colonies (Invitrogen) using QIAprep Spin Miniprep Kit (Qiagen), linearized with *Nsi*I and introduced by transformation at the *lys1+* locus of *cnp1-76* (Castillo, Mellone et al. 2007) and *cen2-tetO-tdTomato* (Sakuno and Watanabe 2009). For ChIP-qPCR experiments, *S. pombe* cells were grown overnight at 30°C in rich medium YEA and 2 μl of anti-GFP (ab290, Abcam) was used for each immunoprecipitation. PCR oligonucleotides detecting central core (*cc1&3; ura4*), heterochromatic *dg* and euchromatin control *fbp1* were previously reported (Folco, McCue et al. 2019).

### Ortholog identification

Fungal proteomes were retrieved from NCBI. Ortholog identification was performed using OrthoFinder (Emms and Kelly 2019). Remote homology detection was performed using PSI-BLASTp (Altschul, Madden et al. 1997) and HMMER v.3.x.x (http://www.hmmer.org) (E-value < 10^−5^) using *S. pombe* and *S. cerevisiae* proteins as seeds. Ortholog results were further validated by BLAST searches on NCBI website, and searches in eggNOG 6.0 (Hernandez-Plaza, Szklarczyk et al. 2023). Gene reannotations were performed using protein-to-genome alignment with Exonerate (Slater and Birney 2005) followed by manual adjustments if necessary (cf. Supplementary Methods). *P. macacae* strain P3C was sequenced using Oxford nanopore sequencing (cf. Supplementary Methods). Protein multiple sequence alignments were generated using Clustal Omega (Sievers, Wilm et al. 2011). Maximum likelihood phylogeny was performed using RAxML-ng (Kozlov, Darriba et al. 2019). Predicted histones phylogenetic classification was performed with reference protein sequences identified by (Postberg, Forcob et al. 2010). Immunogenetic peptides were selected for *Pneumocystis* CENP-A, CENP-C and mis12 orthologs, used for antibody production and quality control (Supplementary Methods).

### Light, electron and immunofluorescence microscopy

*P. murina* and A549-LET1 cells described above were grown on Thermanox^™^ coverslips (Ted Pella, Redding, CA, USA), harvested at day 7, fixed with 2% paraformaldehyde/2.5% glutaraldehyde in 0.1 M Sorenson’s phosphate buffer and then post-fixed with 1.0% osmium tetroxide/0.8% potassium ferricyanide in 0.1 M sodium cacodylate buffer for 1 hour, washed with buffer then stained with 1% tannic acid in dH_2_O for 1 hr. After additional buffer washes, the samples were further osmicated with 2% osmium tetroxide in 0.1M sodium cacodylate, then washed with dH_2_O. Specimens were then stained overnight at 4C with 1% aqueous uranyl acetate. The cells were then washed with dH_2_O and dehydrated with a graded ethanol series, prior to embedding in Spurr’s resin. Thin sections were cut with a Leica UC7 ultramicrotome (Buffalo Grove, IL) prior to viewing at 120 kV on a FEI BT Tecnai transmission electron microscope (ThermoFisher/FEI, Hillsboro, OR). Digital images were acquired with a Gatan Rio camera (Gatan, Pleasanton, CA).

Fluorescence labelling of *Pneumocystis*-infected tissues was performed by Histoserv, Inc (Germantown, Maryland) and visualized using Leica DMi8 microscope (DMI Medical Inc).

*S. pombe* cells were grown overnight at 32°C in minimal medium PMG until logarithmic phase and then mounted in PMG 2% agarose as described (Tran, Paoletti et al. 2004). Images were acquired on a Delta Vision Elite microscope (Applied Precision) with a 100X 1.35 NA oil lens (Olympus). Twenty 0.35-μm z sections were acquired generating maximum intensity projections. Further image processing including background-subtracted centromere foci intensity measurements, was performed using ImageJ (National Institutes of Health).

### ChIP-seq

Purified cells (~5 × 10^6^ cells) and tissue preparations were fixed by 37% formaldehyde treatment. Chromatin preparation, immunoprecipitation and Illumina sequencing libraires were prepared using the Low Cell ChiP-Seq kit (Active Motif, Carlsbad, CA) according to the manufacturer instructions. ChIP-seq libraries were sequenced commercially (Psomagen Rockville, Maryland, USA) using Illumina NovaSeq 6000 (150-bp paired end reads).

### RNA-Seq

Total RNA was extracted using RNeasy Mini kit (Qiagen). RNA quality and integrity were estimated using Bioanalyzer RNA 6000 Pico Assay (Agilent) and sequenced commercially using Illumina HiSeq 4000 (150-base paired end libraries, Novogene Inc, USA). Reads were mapped using STAR v.2.7.9a (Dobin, Davis et al. 2013), and normalized to bins per million mapped reads (BPM). Transcripts counts were quantified using kallisto 0.44.0 (Bray, Pimentel et al. 2016).

### ChIP-seq peak calling and sequence analysis

Raw reads were quality-checked with multi-QC (Ewels, Magnusson et al. 2016). Host reads (human, macaque, rat, mice) were removed by mapping using Bowtie2 (Langmead and Salzberg 2012) with default parameters. Unmapped reads were retrieved and mapped to *Pneumocystis* reference genomes (*P. murina* (GenBank accession number GCA_000349005.2), *P. carinii* (GCA_001477545.1), *P. macacae* (GCA_018127085.1) and *P. jirovecii* (GCA_001477535.1)) using the Burrows-Wheeler Aligner BWA-MEM (Li 2013). Alignments were filtered and sorted using SAMTools (Li, Handsaker et al. 2009) and duplicates marked using PICARD MarkDuplicates http://broadinstitute.github.io/picard). Quality controls were performed using deepTools plotFingerprint (Ramirez, Ryan et al. 2016) and phantompeakqualtools (Landt, Marinov et al. 2012), which estimate the normalized strand coefficient (NSC) and the relative strand correlation (RSC). Chip-Seq coverage data were calculated as 50-bp bin and normalized per genomic content (1x normalization). Chromosome ideograms were generated using chromoMap v0.3 (Lakshay Anand 2020).

ChIP enrichment peaks were detected using Model-Based Analysis for ChIP-Sequencing (Zhang, Liu et al. 2008)(MACS3 v3.0.0a5) callpeak function with default parameters and estimated genome sizes and sorted by fold-enrichment and FDR values (broad cutoff 0.1; FDR < 0.05). Enriched peaks were further inspected using deepTools bamCompare versus shuffle non centromeric genomic regions. Peaks were further examined in conjunction with other data (e.g. conservation score, RNA-seq, methylation data, GC content, repeats) with the Integrative Genomics Viewer (Robinson, Thorvaldsdottir et al. 2011), ensuring that peaks are present in all IP samples and absent in controls. *De novo* DNA motif searches were performed using MEME v5.1.0 (Bailey, Boden et al. 2009) and Homer v4.9 (Heinz, Benner et al. 2010) with a p-value cut off of 10^−10^. GC content across windows (bin) was computed using BEDTools (Quinlan and Hall 2010). Normalized coverage and annotations across chromosomes were visualized using pyGenomeTracks (Lopez-Delisle, Rabbani et al. 2021).

Repeated elements (DNA transposons, retrotransposons and low complexity repeats) were identified using RepeatMasker (Smit AFA. 2013–2015) and RepBase (Bao, Kojima et al. 2015).

### Genome synteny and conservation

DNA alignments were generated using Satsuma2 (Grabherr, Russell et al. 2010) and visualized with Circos (Krzywinski, Schein et al. 2009). To estimate the sequence conservation scores, we performed one-to-one pairwise whole genome alignments using LAST (Kielbasa, Wan et al. 2011) with the MAM4 seeding scheme (Frith and Noe 2014). We consider two trios (1) *P. jirovecii*/*P. macacae*/*P. oryctolagi* and (2) *P. carinii*/*P. murina*/*P. wakefieldiae*. We generated a neutral model using phyloFit (Siepel and Haussler 2004) from PHAST which fit the tree model to multiple sequence alignments by maximum likelihood using the REV substitution model. Sequence conservation scores were estimated using phastCons (Siepel, Bejerano et al. 2005) (--target-coverage 0.25 --expected-length 20 --estimate-trees). Wig files and Bedgraph files were converted using ‘wigToBigWig’ and ‘bigWigToBedGraph’ tools (Kent, Zweig et al. 2010).

### Bisulfite sequencing

*Pneumocystis* DNA (5 μg) was extracted by a *Pneumocystis* DNA enrichment protocol (Ma, Chen et al. 2016). Bisulfite conversion, library preparation and sequencing (Illumina NovaSeq 150 bp paired end reads) were performed commercially (Novogene). Bisulfite conversion of non-methylated DNA was performed using EZ DNA Methylation kit. Data analysis was performed using BSMAP and methratio script (Xi and Li 2009). Only cytosine positions with more than 5-fold coverage were considered. We used weighted methylation percentage (Cook, Kramer et al. 2020), which is calculated as the number of reads supporting methylation over the number of cytosines sequenced to quantify methylation levels. Methylation data were partitioned over different genomic compartments using BEDTools (Quinlan 2014). Statistical comparisons were performed using R (https://www.R-project.org) to compute non-parametric Mann-Whitney U test and Bonferroni correction to adjust *P*values for multiple comparisons.

### ChIP-qPCR

CENP-A enrichment and H3 depletion relative to H4 within the centromeres were evaluated using primers specific to centromeric and non-centromeric loci (see Supplementary Table 5 for primers). Primers were designed using primer3 (Koressaar, Lepamets et al. 2018) from regions visualized using IGV genome browser. Primers with potential matches against mammal genomes were excluded using NCBI BLASTn. Dilutions of 1:10 were used based on preliminary test runs. Each reaction contains 5 μl of iTaq universal Sybr green supermix (Biorad), 1.25 μl of primers (500 nM)) and 2.5 μl of DNA. All assays were run on CFX96 thermocycler (Biorad). Three technical replicates were taken for each assay, and the standard errors of the mean were calculated. The PCR program was as follow: initial denaturation for 2 min at 95°C, followed by 30 cycles of 30 seconds at 95°C, 60°C for 30s, and 72° for 30s. Locus ΔCt values were normalized using the formula: (^ΔΔCt^ ChIP – ^ΔCt^ Input; https://www.sigmaaldrich.com/US/en/technical-documents/protocol/genomics/qpcr/chip-qpcr-data-analysis) and the fold enrichment of the ChIP DNA over the input was computed as log2 (2^ΔΔCt^). The plots and statistical analyses were performed with GraphPad Prism 8.

### ChIP-LC-MS

Formalin-fixed purified chromatin preparations were separately co-immunoprecipitated with anti CENP-A, CENP-C, H4 and pre immune serum (control) using Pierce^™^ Direct Magnetic IP/Co-IP Kit (ThermoFisher). Each IP reaction was performed separately on aliquots from the same chromatin preparation. Elution was performed using 1% formic acid. Extracts were frozen in dry ice, lyophilized for 1 hour and digested using trypsin, diluted, and injected to a Thermo Orbitrap Fusion LC-MS/MS to identify unique peptides. LC-MS/MS data were searched against the proteomes using Proteome Discoverer 2.4 (Thermo Fisher Scientific, Waltham, MA). Label-free quantification was analyzed based on the peak intensity of the precursor ion.

## Supplementary Material

Supplement 1 Supplementary Methods.
**Supplementary figure 1.** Conservation of kinetochore, chromatin formation, chromatin modifying methyltransferases, DNA methylation and RNAi components in Taphrinomycotina and other representative fungi.
**Supplementary figure 2.** Antibody selection and testing
**Supplementary figure 3.** *Pneumocystis* CENP-A binds to single genomic foci in replicating cells.
**Supplementary figure 4.** CENP-A enrichment at *Pneumocystis* centromeres by ChIP-qPCR.
**Supplementary figure 5.** Genomic view of *P. macacae* genome showing pericentromeric heterochromatin.
**Supplementary figure 6**. Genomic view of *P. carinii* genome showing pericentromeric heterochromatin.
**Supplementary figure 7**. Genomic view of *P. murina* genome showing pericentromeric heterochromatin.
**Supplementary figure 8.** Centromeres are flanked by heterochromatin and contain active genes.
**Supplementary figure 9.** Analysis of canonical histone H3 at *Pneumocystis* centromeres by ChIPq-PCR.
**Supplementary figure 10.** 5-methylcytosine (5mC) DNA methylation in *Pneumocystis*
**Supplementary figure 11.** Genome synteny and centromere locations in *Pneumocystis species*.
**Supplementary table 1.** Coordinates, length, and GC content (in %) of Pneumocystis centromeres identified by direct ChIP.
**Supplementary table 2.** Expression of genes located in homologous *P. murina* and *P. carinii* centromeres.
**Supplementary table 3.** Persistent hydrophobic/nonpolar interactions between the tetramer (H4:CENP-A)2 and dimers (H2A:H2B)
**Supplementary table 4.** Centromeres sequence similarity across *Pneumocystis* species.
**Supplementary table 4.** *Pneumocystis* ChIP-qPCR oligo nucleotides
Key Resources table.
**Supplementary dataset 1.** *P. jirovecii* and *P. murina* CENP-A full-length and N-terminal truncated DNA sequences codon optimized for *S. pombe* codon bias.
**Supplementary dataset 2.** Dynamic animations of the molecular dynamics trajectories showing both sides of each octameric complex in *P. carinii*, *P. murina* and *S. pombe*.

## Data Availability

ChIP-seq datasets and associated MACS2 outputs generated in this study (CENP-A, CENP-C, Mis12, H3, H4, H3K9me2/3, H3K4me2) are deposited at NCBI GEO (accession numbers GSE226536, GSE230598).
