# Supplementary material for: The Host Adapted Fungal Pathogens of Pneumocystis Genus Utilize Genic Regional Centromeres
Source: bioRxiv. 2023 May 12:2023.05.12.540427. Preprint. [Version 1] doi: 10.1101/2023.05.12.540427 (PMC10327204; doi:10.1101/2023.05.12.540427)
Supplement: Supplement 1 — Supplementary Methods. Supplementary figure 1. Conservation of kinetochore, chromatin formation, chromatin modifying methyltransferases, DNA methylation and RNAi components in Taphrinomycotina and other representative fungi. Supplementary figure 2. Antibody selection and testing Supplementary figure 3. Pneumocystis CENP-A binds to single genomic foci in replicating cells. Supplementary figure 4. CENP-A enrichment at Pneumocystis centromeres by ChIP-qPCR. Supplementary figure 5. Genomic view of P. macacae genome showing pericentromeric heterochromatin. Supplementary figure 6. Genomic view of P. carinii genome showing pericentromeric heterochromatin. Supplementary figure 7. Genomic view of P. murina genome showing pericentromeric heterochromatin. Supplementary figure 8. Centromeres are flanked by heterochromatin and contain active genes. Supplementary figure 9. Analysis of canonical histone H3 at Pneumocystis centromeres by ChIPq-PCR. Supplementary figure 10. 5-methylcytosine (5mC) DNA methylation in Pneumocystis Supplementary figure 11. Genome synteny and centromere locations in Pneumocystis species. Supplementary table 1. Coordinates, length, and GC content (in %) of Pneumocystis centromeres identified by direct ChIP. Supplementary table 2. Expression of genes located in homologous P. murina and P. carinii centromeres. Supplementary table 3. Persistent hydrophobic/nonpolar interactions between the tetramer (H4:CENP-A)2 and dimers (H2A:H2B) Supplementary table 4. Centromeres sequence similarity across Pneumocystis species. Supplementary table 4. Pneumocystis ChIP-qPCR oligo nucleotides Key Resources table. Supplementary dataset 1. P. jirovecii and P. murina CENP-A full-length and N-terminal truncated DNA sequences codon optimized for S. pombe codon bias. Supplementary dataset 2. Dynamic animations of the molecular dynamics trajectories showing both sides of each octameric complex in P. carinii, P. murina and S. pombe. [file media-1.pdf]

Supplementary Material for

The Host Adapted Fungal Pathogens of *Pneumocystis* Genus Utilize Genic Regional Centromeres

Authors:
Ousmane H. Cissé, Shelly Curran, H. Diego Folco, Yueqin Liu, Lisa Bishop, Honghui Wang, Elizabeth R. Fischer, A Sally Davis, Spenser Babb-Biernacki, Vinson P. Doyle, Jonathan K. Richards, Sergio A. Hassan, John P. Dekker, Pavel P. Khil, Jason M. Brenchley, Shiv Grewal, Melanie Cushion, Liang Ma and Joseph A. Kovacs

Correspondence to: ousmane.cisse@nih.gov and/or jkovacs@mail.nih.gov

This file includes:

Supplementary methods, key resource table,
supplementary table 3 and supplementary figures 1 to 11

Supplementary Methods 3
P. macacae nanopore sequencing 3
Gene annotation corrections. 3
Immunogenic peptides selection, antibody production, and quality control 4
Verifying antibody reactivity with *Pneumocystis* 5
Modeling and Simulations 5
Key Resources table 8
Supplementary table 3: hydrophobic/nonpolar interactions 15
Additional citations: 18

Supplementary Methods

P. macacae nanopore sequencing

Pneumocystis macacae strain P3C genome DNA was extracted by a *Pneumocystis* DNA enrichment protocol (Ma, Chen et al. 2016) and sequenced using GridION X5 and MinION Mk1B Oxford Nanopore Technologies (Oxford, UK). For sequencing performed on the MinION device, host sequences were selectively ejected by mapping to the *P. macacae* strain P2C genome (Cisse, Ma et al. 2021) (NCBI accession no. GCA_018127085.1) in real-time to enrich for *Pneumocystis* reads using the program UNCALLED (Kovaka, Fan et al. 2021). Chunk size was set to 0.5 to maximize target enrichment; all other settings were kept at the default. Host DNA reads removal and *P. macacae* strain P3C *de novo* genome assembly were performed using protocols previously described (Cisse, Ma et al. 2021).

Gene annotation corrections.

We refined the annotation of the gene encoding the CENP-A homologues in current *Pneumocystis* genome assemblies based on detailed comparative sequence analysis including using RNA-Seq data and whole genome sequencing data from additional *Pneumocystis* strains. Although all *Pneumocystis* genomes encode a two-introns containing *cenp-a* histone gene, full length protein alignments indicate errors in some *Pneumocystis* predicted CENP-A homologs. First, the annotated CENP-A protein in the *P. jirovecii* reference genome (strain RU7) (Ma, Chen et al. 2016) was truncated in its C-terminal region likely due to an annotation error in an intron splicing site. A corrected version of the gene was annotated using published RNA -seq data (Cisse, Pagni et al. 2012, Cisse, Ma et al. 2018) and protein to DNA alignment of *P. carinii* orthologs. The *cenp-a* gene sequences in assemblies of two other *P. jirovecii* strains were partial due to incomplete assemblies (SE8 (Cisse, Pagni et al. 2012) and SE2178 (Schmid-Siegert, Richard et al. 2017). Using phylogenetic analysis, we found that the protein annotated as CENP-A in *P. macacae* genome (strain P2C) (Cisse, Ma et al. 2021) was in fact a

canonical H3 histone. We annotated the valid *cenp-a-* gene which had been excluded because of partial overlap with another gene. To further confirm, we sequenced the genome of another *P. macacae* strain (P3C) using Illumina and Nanopore technologies and found an identical *cenp-a* gene (cf. *P. macacae* nanopore sequencing). We constructed a maximum likelihood phylogeny, which confirmed that *P. macacae* CENP-A protein clusters with CENP-A histones apart from canonical H3 histones. The CENP-A protein annotated in the *P. murina* reference genome (strain B123) (Ma, Chen et al. 2016) was truncated in the C-terminus due to error causing a premature stop codon. The CENP-A protein- in the *P. oryctolagi* genome (Cisse, Ma et al. 2021), the species infecting rabbits was truncated in the N-terminus and corrected. Corrected CENP-A protein sequences are presented in **Figure 1a**. Unexpectedly, the histone H4 gene was not annotated in all *P. jirovecii* assemblies (RU7, SE8, SE2178), thought present in other species (e.g., *P. carinii*). We annotated the *h4* gene genome using tBLASTn and protein alignment to genomes and confirmed the presence of the protein by immunofluorescence (**Figure 1c**).

Immunogenic peptides selection, antibody production, and quality control

To characterize *pn*CENP-A, we focused on four species of which sufficient material for ChIP-seq studies were available: *P. macacae*, *P. carinii* and *P.* murina and to a lesser extend *P. jirovecii*. We identified immunogenic peptides in *Pneumocystis* CENP-A, CENP-C and mis12 (Supplementary Figure 2 panels a, b, d). We used BLAST searches to remove peptides with matches against host genomes. These peptides have no sequence matches against host predicted proteomes (e.g., human, macaques, mouse, and rats). Yet, the generated antibodies showed some level of cross reactivity against the host cell lysates by western blots and immunofluorescence (**Supplementary Figure 2 panels e and f**). Rabbit antibodies targeting *Pneumocystis* CENP-A, CENP-C were generated by from GenScript (see key resource table). *P. carinii* and *P. murina* shared the same immunogens for CENP-A and CENP-C, and the obtained antibodies work for both species. *P. jirovecii* CENP-A antibodies are specific to this species and Pj/Pmac CENP-C antibodies recognize both *P. jirovecii* and *P. macacae*. Commercial antibodies targeting histones (H3, H4, H3K9me2/3, H3K4me2) were obtained from

Abcam (see **key resource table**). For commercial antibodies, we obtained the epitopes from the company and confirmed their conservation in *Pneumocystis* proteins.

Verifying antibody reactivity with *Pneumocystis*

We tested all custom antibodies for reactivity with *Pneumocystis* using a variety of methods described below prior ChIP-seq studies. Anti-CENP-A and anti-CENP-C reacted with the immunogenic peptides, SDS extracted cell lysates and formalin fixed chromatin with a minimal background and no cross reactivity with the uninfected host proteins.

Pneumocystis proteins (antigens) were prepared using glass beads. Partially purified organism pellets were resuspended in 1X PBS buffer. 0.65 ml of 0.5 mm glass beads (Biospec Products, Inc) was added to the suspensions and vortexed for 5 minutes at 4°C. Beads were allowed to settle for less 30 seconds and the supernatant transferred to a new tube for sonication. The samples were centrifuged for 20, 0000 X g for 10 minutes and the supernatants were transferred to a new tube. The pellet was resuspended in 20 μl PBS.

For western blots, cell lysates and formalin-fixed chromatin preparations were run on Tris-Glycine SDS 4-20% wedgewell gel (Thermo Fisher Scientific) and transferred on nitrocellulose membranes by incubation at room temperature for 30 minutes, followed by 1 hour incubation in blocking buffer (1X PBS + 5% milk + 0.05% Tween 20) with shaking. Nitrocellulose blots were incubated with primary antibodies (1:100) diluted in blocking buffer for an hour with shaking, followed by a washing and then 1 hour incubation with secondary anti rabbit or anti mouse IgG HRP goat polyclonal antibody (1:2000) (PRF&L) and developed with Pierce 1-Step^{TM} Ultra TMB-Blotting solution (Pierce).

Modeling and Simulations

Models of centromeric octamers were created from the predicted AlphaFold (Jumper, Evans et al. 2021) structure of individual histones and assembled by backbone superposition on the human

centromeric nucleosome (PDB: 3AN2). Besides the revised CENP-A sequences of *Pneumocystis* reported in this study, the UniProt AC of the proteins used here (cf. Figure 5a) are as follows (H4, CENP-A, H2A, and H2B): *P. carinii* (A0A0W4ZDE3, A0A0W4ZSS3, A0A0W4ZH07, A0A0W4ZGY7); *P. murina* (M7PG14, corrected annotation, M7NMV0, M7NIN8); *S. pombe* (P09322, Q9Y812, P04909, P04913); alternate annotations exist for the same strains of H2A and H2B but not considered here. All the simulations were performed with NAMD (Brooks, Brooks et al. 2009) using the all-atom CHARMM forcefield (param36) in the NPT ensemble, at 37 ^{0}C and 1 atm, at [NaCl] ~150 mM and pH 7 (all residues were uncharged except Asp^{-}, Glu^{-}, Arg^{+}, and Lys^{+}; ions were added as needed to neutralize the system; N- and C-termini of each histone protein were uncapped). The side length of the simulation box was initially set at ~12.0 nm and filled with ~56,000 TIP3P water molecules, yielding an average density of ~0.993 g/cm^3 after equilibration. Simulations were performed in a cubic cell with PBC and PME; all bond lengths involving hydrogen atoms were constrained with the SHAKE algorithm (Ryckaert, Ciccotti et al. 1977), and an integration step of 2 fs was used. Each system was heated and equilibrated for 10 ns, followed by a productive phase of 50 ns. Soft definitions of H-bond/salt-bridge and hydrophobic/non-polar interactions are based on a distance (δ) criterion between donor and acceptor atoms ($\delta_{AD} < 4$ Å) and side-chain carbon atoms ($\delta_{CC} < 4.8$ Å), respectively (analysis scripts available online). The number and persistence of the interaction throughout the simulation are thus proxies for the strength of an interaction between two residues. Except when indicated, the main analysis focused on the core structure of the octamers and

not on the N-terminal tails since these are disordered and poorly predicted by AF and are affected by the wrapped DNA, which was not included. All the analyses were performed with CHARMM.

Key Resources table

**Reagent type (species) or resource**	**Designation**	**Source or reference**	**Identifiers**	**Additional information**
Biological sample (*Pneumocystis macacae*)	P2C	NIAID (Cisse *et al.* 2021)	NCBI taxon id 2698480	Isolated from *Macaca fascicularis* with PCP/ SIV infection
Biological sample (*Pneumocystis macacae*)	P3C	NIAID (collected 2021)	NCBI taxon id 2698480	Isolated from *Macaca fascicularis* with PCP/ SIV infection
Biological sample (*Pneumocystis jirovecii*)	ZA	Ma *et al.* 2016 Cisse *et al.* 2018	NCBI taxon id 42068	Isolated from human lungs postmortem
Biological sample (*Pneumocystis jirovecii*)	SP	Cisse *et al.* 2018	NCBI taxon id 42068	Isolated from human lungs postmortem
Biological sample (*Pneumocystis carinii*)	Ind Rat 1 Date: 01/18/00 Infection level: 5+	Indiana University (contract)	NCBI taxon id 1408658	Corticoid immune suppressed rats
Biological sample (*Pneumocystis carinii*)	Ind Rat 2 Date: 11/7/00 Infection level: 4-5+	Indiana University (contract)	NCBI taxon id 1408658	Corticoid immune suppressed rats

Biological sample (*Pneumocystis carinii*)	Ind Rat 4 Date: 11/21/00 Infection level: 4-5+	Indiana University (contract)	NCBI taxon id 1408658	Corticoid immune suppressed rats
Biological sample (*Pneumocystis carinii*)	Ind Rat Date: 03/22/22 Infection level: 4-5+	U. Cincinnati	NCBI taxon id 1408658	Corticoid immune suppressed rats
Biological sample (*Pneumocystis murina*)		This study	NCBI taxon id 263815	CD40L KO
Cell line	A549	ATCC # CC-185		Used for *P. murina* culture
Cell line	LET1	BEI Resources # NR-42941		Used for *P. murina* culture
antibody	*P. macacae* anti-CENP-A (Rabbit polyclonal)	GenScript	Cat#: 1617329	IF (1:1000), WB (1:1000) ChIP-Seq (1:100)
antibody	*P .macacae*/*P. jirovecii* anti-CENP-C (Rabbit polyclonal)	GenScript	Cat#: 1617329	IF (1:1000) WB (1:1000) ChIP-seq (4 µg for 30 µl reaction)
antibody	*P .macacae* anti-mis12 (Rabbit polyclonal)	GenScript	Cat#: U0148GF150	ChIP-seq (4 µg for 30 µl reaction)

antibody	*P. jirovecii* anti-CENP-A (Rabbit polyclonal)	GenScript	Cat#: 1617329	IF (1:1000), WB (1:1000) ChIP-Seq (4 µg for 30 µl reaction)
antibody	*P. carinii/P. murina* anti-CENP-A (Rabbit polyclonal)	GenScript	Cat#: 1598083	IF (1:1000), WB (1:1000) ChIP-Seq (4 µg for 30 µl reaction)
antibody	*P. carinii/P. murina* anti-CENP-C (Rabbit polyclonal)	GenScript	Cat#: 1525058	IF (1:1000), WB (1:1000) ChIP-Seq (4 µg for 30 µl reaction)
antibody	Pan *Pneumocystis* (cross-reacts with all known *Pneumocystis*)	(Kovacs, Halpern et al. 1989)	7D7	WB (1/1000) ELISA (1/1000)
antibody	Anti-mouse IgG HRP (goat polyclonal)	PRF&L	Anti-mouse IgG HRP	WB (1:2000) ELISA (1:1000)
antibody	Anti-rat IgG HRP (goat polyclonal)	PRF&L	Anti-rat IgG HRP	WB (1:2000) ELISA (1:1000)
antibody	Anti-rabbit IgG HRP (goat polyclonal)	PRF&L	Anti-rabbit IgG HRP	WB (1:2000) ELISA (1:1000)

antibody	Anti-H3 (rabbit polyclonal)	Abcam	Cat#: ab1791	ChIP (1:100)
antibody	Histone H3K4me2 (rabbit polyclonal)	Active motif	Cat#: 39142	ChIP (1:100)
antibody	Anti-Histone (di methyl K9) H3K9me2	Abcam	Cat#: ab1220	ChIP (1:100)
antibody	Anti-Histone H3 (tri methyl K9)	Abcam	Cat#: ab8898	ChIP (1:100)
antibody	Anti-Histone H3 (tri methyl K4)	Abcam	Cat#: ab8580	ChIP (1:100)
antibody	Anti-H4 (rabbit polyclonal)	Abcam	Cat#: ab10158	ChIP (1:100)<br>IF (1:200)<br>IHC (1:100)
commercial assay or kit	Low Cell ChIP-Seq kit	Active motif	Cat#: 53084	
commercial assay or kit	MasterPure Yeast DNA purification kit	LUCIGEN	Cat # MPY80200	
Synthetic DNA	Pj_CENPA_full	GenScript	Cat # U247RHD070	Full length *P. jirovecii*

				CENP-A CDS (codon optimized for *S. pombe*) *
Synthetic DNA	Pj_CENPA_tr1	GenScript	Cat # U247RHD070	N-terminal truncated *P. jirovecii* CENP-A CDS (codon optimized for *S. pombe*) *
Synthetic DNA	Pm_CENPA_full2	GenScript	Cat # U247RHD070	Full length *P. murina* CENP-A CDS (codon optimized for *S. pombe*) **
Synthetic DNA	Pm_CENPA_tr1	GenScript	Cat # U247RHD070	N-terminal truncated *P. jirovecii* CENP-A CDS (codon optimized for *S. pombe*) *
recombinant DNA reagent	pS2 – (plasmid)		Cat # U247RHD070	Empty pS2 provided by D. Folco
recombinant DNA reagent	Pm_CENP-A_full2_pS2 (plasmid)	GenScript	Cat # U1773HC290-12	SC1017 Subcloning bundled with gene synthesis: Pm_CENP-A_full2_pS2

Synthetic peptide (Immunogen)	*P. jirovecii* CENP-A	GenScript	Cat#: 1617329	AGRPSDAP RTGRKRC
Synthetic peptide (Immunogen)	*P. macacae* CENP-A	GenScript	Cat#: xxxxx	AGRAGEGVR GGKRC
Synthetic peptide (Immunogen)	*P .macacae/P. jirovecii* CENP-C	GenScript	Cat#: 1617329	CLPRGGEKTT KPSKH
Synthetic peptide (Immunogen)	*P. carinii/P. murina* CENP-C	GenScript	Cat#: 1525058	CLPRGGEKAT KPSKH
Synthetic peptide (Immunogen)	*P. carinii/P. murina* CENP-A	GenScript	Cat#: 1598083	IEPGDPIPRGK ARRC
Synthetic peptide (Immunogen)	*P. murina* RNA polymerase II	GenScript	Cat#: U8245GB220-4	AKIEYPEIM DESGHC (Lot: U1721GC110 -1) GKTPLQDD QATPYDSRS PC (Lot: U2655GC110 -1)
Synthetic peptide (Immunogen)	*P. macacae mis12*	GenScript	Cat#: U0148GF150	CHQMDVD FSKSEKDF (Lot: U8960GG160 -1) CDISKEYDV KPLFET (Lot: U8348GG160 -1)

Sequence-based reagent	List of primers used in this study	IDT	Cat # 18474072	Supplementary Table 4

Supplementary table 3: hydrophobic/nonpolar interactions

Persistent hydrophobic/nonpolar interactions between the tetramer (H4: CENP-A)$_2$ and dimers (H2A:H2B) as estimated from the dynamics simulations (cf. Fig. S2 and Methods); rightmost column shows normalized frequencies; freq. < 5 and interactions with N-terminal tails not included (except gray lines in *P. murina*; see text). See sequence alignments in Figure 5.

P. carinii

CENP-A	(A)	58	ILE	H2A	(G)	113	ILE	5
CENP-A	(A)	114	VAL	H2A	(G)	109	VAL	10
CENP-A	(A)	122	LEU	H2A	(G)	117	LEU	10
CENP-A	(A)	125	ILE	H2A	(G)	117	LEU	5
CENP-A	(A)	125	ILE	H2A	(G)	130	LEU	15
H4	(B)	73	TYR	H2B	(D)	87	LEU	15
H4	(B)	73	TYR	H2B	(D)	90	TYR	10
H4	(B)	73	TYR	H2B	(D)	107	LEU	20
H4	(B)	89	TYR	H2B	(D)	90	TYR	85
H4	(B)	98	ILE	H2A	(G)	105	ALA	5
H4	(B)	99	TYR	H2A	(G)	99	LEU	10
H4	(B)	99	TYR	H2A	(G)	102	VAL	30
H4	(B)	99	TYR	H2A	(G)	104	ILE	15
H4	(B)	101	PHE	H2A	(G)	105	ALA	15
H4	(B)	99	TYR	H2B	(H)	68	ILE	20
H4	(B)	99	TYR	H2B	(H)	72	PHE	25
H4	(B)	100	GLY	H2B	(H)	68	ILE	10
H2A	(C)	109	VAL	CENP-A	(E)	114	VAL	10
H2A	(C)	117	LEU	CENP-A	(E)	55	LEU	10
H2A	(C)	117	LEU	CENP-A	(E)	58	ILE	15
H2A	(C)	99	LEU	H4	(F)	99	TYR	10
H2A	(C)	102	VAL	H4	(F)	99	TYR	30
H2A	(C)	104	ILE	H4	(F)	99	TYR	15
H2A	(C)	105	ALA	H4	(F)	98	ILE	5
H2A	(C)	105	ALA	H4	(F)	101	PHE	10
H2B	(D)	68	ILE	H4	(F)	99	TYR	20
H2B	(D)	68	ILE	H4	(F)	100	GLY	10
H2B	(D)	72	PHE	H4	(F)	99	TYR	25
H4	(F)	73	TYR	H2B	(H)	87	LEU	15
H4	(F)	73	TYR	H2B	(H)	90	TYR	15
H4	(F)	73	TYR	H2B	(H)	107	LEU	15
H4	(F)	89	TYR	H2B	(H)	90	TYR	95

P. murina

CENP-A	(A)	58	ILE	H2A	(G)	113	ILE	5
CENP-A	(A)	114	VAL	H2A	(G)	109	VAL	10
CENP-A	(A)	125	ILE	H2A	(G)	117	LEU	5
H4	(B)	73	TYR	H2B	(D)	89	LEU	10
H4	(B)	73	TYR	H2B	(D)	92	TYR	10
H4	(B)	73	TYR	H2B	(D)	109	LEU	15
H4	(B)	89	TYR	H2B	(D)	92	TYR	80
H4	(B)	99	TYR	H2A	(G)	99	LEU	10
H4	(B)	99	TYR	H2A	(G)	102	VAL	30
H4	(B)	99	TYR	H2A	(G)	104	ILE	15
H4	(B)	101	PHE	H2A	(G)	105	ALA	15
H4	(B)	99	TYR	H2B	(H)	70	ILE	20
H4	(B)	99	TYR	H2B	(H)	74	PHE	25
H4	(B)	100	GLY	H2B	(H)	70	ILE	10
H2A	(C)	109	VAL	CENP-A	(E)	114	VAL	15
H2A	(C)	132	VAL	CENP-A	(E)	40	ILE	5

H2A	(C)	132	VAL	CENP-A	(E)	43	GLY	10
H2A	(C)	135	ILE	CENP-A	(E)	27	LEU	10
H2A	(C)	135	ILE	CENP-A	(E)	40	ILE	10
H2A	(C)	136	ILE	CENP-A	(E)	40	ILE	20
H2A	(C)	138	ILE	CENP-A	(E)	27	LEU	10
H2A	(C)	139	VAL	CENP-A	(E)	27	LEU	5
H2A	(C)	139	VAL	CENP-A	(E)	40	ILE	10
H2A	(C)	142	TYR	CENP-A	(E)	27	LEU	20
H2A	(C)	142	TYR	CENP-A	(E)	29	GLY	5
H2A	(C)	142	TYR	CENP-A	(E)	36	PRO	15
H2A	(C)	143	LEU	CENP-A	(E)	36	PRO	10
H2A	(C)	143	LEU	CENP-A	(E)	37	GLY	5
H2A	(C)	99	LEU	H4	(F)	99	TYR	10
H2A	(C)	102	VAL	H4	(F)	99	TYR	30
H2A	(C)	104	ILE	H4	(F)	99	TYR	15
H2A	(C)	105	ALA	H4	(F)	98	ILE	5
H2A	(C)	105	ALA	H4	(F)	101	PHE	15
H2B	(D)	70	ILE	H4	(F)	99	TYR	20
H2B	(D)	70	ILE	H4	(F)	100	GLY	10
H2B	(D)	74	PHE	H4	(F)	99	TYR	25
H4	(F)	73	TYR	H2B	(H)	89	LEU	15
H4	(F)	73	TYR	H2B	(H)	92	TYR	10
H4	(F)	73	TYR	H2B	(H)	109	LEU	15
H4	(F)	89	TYR	H2B	(H)	92	TYR	100

S. pombe

CENP-A	(A)	29	LEU	H2A	(G)	113	ILE	10
CENP-A	(A)	29	LEU	H2A	(G)	118	LEU	5
CENP-A	(A)	29	LEU	H2A	(G)	119	PRO	10
CENP-A	(A)	32	ILE	H2A	(G)	113	ILE	15
CENP-A	(A)	88	VAL	H2A	(G)	109	VAL	10
CENP-A	(A)	99	ILE	H2A	(G)	117	LEU	10
CENP-A	(A)	104	VAL	H2A	(G)	117	LEU	10
H4	(B)	73	TYR	H2B	(D)	80	LEU	10
H4	(B)	73	TYR	H2B	(D)	83	TYR	10
H4	(B)	73	TYR	H2B	(D)	100	LEU	20
H4	(B)	89	TYR	H2B	(D)	83	TYR	85
H4	(B)	98	ILE	H2A	(G)	105	ALA	5
H4	(B)	99	TYR	H2A	(G)	99	LEU	10
H4	(B)	99	TYR	H2A	(G)	102	VAL	30
H4	(B)	99	TYR	H2A	(G)	104	ILE	15
H4	(B)	101	PHE	H2A	(G)	105	ALA	10
H4	(B)	99	TYR	H2B	(H)	61	ILE	15
H4	(B)	99	TYR	H2B	(H)	65	PHE	25
H4	(B)	100	GLY	H2B	(H)	61	ILE	10
H2A	(C)	109	VAL	CENP-A	(E)	88	VAL	10
H2A	(C)	113	ILE	CENP-A	(E)	29	LEU	5
H2A	(C)	113	ILE	CENP-A	(E)	32	ILE	10
H2A	(C)	117	LEU	CENP-A	(E)	99	ILE	15
H2A	(C)	117	LEU	CENP-A	(E)	104	VAL	5
H2A	(C)	118	LEU	CENP-A	(E)	29	LEU	5
H2A	(C)	99	LEU	H4	(F)	99	TYR	10
H2A	(C)	102	VAL	H4	(F)	99	TYR	30
H2A	(C)	104	ILE	H4	(F)	99	TYR	15
H2A	(C)	105	ALA	H4	(F)	98	ILE	5
H2A	(C)	105	ALA	H4	(F)	101	PHE	10
H2B	(D)	61	ILE	H4	(F)	99	TYR	15
H2B	(D)	61	ILE	H4	(F)	100	GLY	10
H2B	(D)	65	PHE	H4	(F)	99	TYR	25
H4	(F)	73	TYR	H2B	(H)	80	LEU	15
H4	(F)	73	TYR	H2B	(H)	83	TYR	10
H4	(F)	73	TYR	H2B	(H)	100	LEU	20
H4	(F)	89	TYR	H2B	(H)	83	TYR	95

Additional citations:

Brooks, B. R., C. L. Brooks, 3rd, A. D. Mackerell, Jr., L. Nilsson, R. J. Petrella, B. Roux, Y. Won, G. Archontis, C. Bartels, S. Boresch, A. Caflisch, L. Caves, Q. Cui, A. R. Dinner, M. Feig, S. Fischer, J. Gao, M. Hodoscek, W. Im, K. Kuczera, T. Lazaridis, J. Ma, V. Ovchinnikov, E. Paci, R. W. Pastor, C. B. Post, J. Z. Pu, M. Schaefer, B. Tidor, R. M. Venable, H. L. Woodcock, X. Wu, W. Yang, D. M. York and M. Karplus (2009). "CHARMM: the biomolecular simulation program." J Comput Chem **30**(10): 1545-1614.

Cisse, O. H., L. Ma, J. P. Dekker, P. P. Khil, J. H. Youn, J. M. Brenchley, R. Blair, B. Pahar, M. Chabe, K. K. A. Van Rompay, R. Keesler, A. Sukura, V. Hirsch, G. Kutty, Y. Q. Liu, L. Peng, J. Chen, J. Song, C. Weissenbacher-Lang, J. Xu, N. S. Upham, J. E. Stajich, C. A. Cuomo, M. T. Cushion and J. A. Kovacs (2021). "Genomic insights into the host specific adaptation of the Pneumocystis genus." Communications Biology **4**(1).

Cisse, O. H., L. Ma, D. Wei Huang, P. P. Khil, J. P. Dekker, G. Kutty, L. Bishop, Y. Liu, X. Deng, P. M. Hauser, M. Pagni, V. Hirsch, R. A. Lempicki, J. E. Stajich, C. A. Cuomo and J. A. Kovacs (2018). "Comparative Population Genomics Analysis of the Mammalian Fungal Pathogen Pneumocystis." MBio **9**(3): e00381-00318.

Cisse, O. H., M. Pagni and P. M. Hauser (2012). "De novo assembly of the Pneumocystis jirovecii genome from a single bronchoalveolar lavage fluid specimen from a patient." MBio **4**(1): e00428-00412.

Jumper, J., R. Evans, A. Pritzel, T. Green, M. Figurnov, O. Ronneberger, K. Tunyasuvunakool, R. Bates, A. Zidek, A. Potapenko, A. Bridgland, C. Meyer, S. A. A. Kohl, A. J. Ballard, A. Cowie, B. Romera-Paredes, S. Nikolov, R. Jain, J. Adler, T. Back, S. Petersen, D. Reiman, E. Clancy, M. Zielinski, M. Steinegger, M. Pacholska, T. Berghammer, S. Bodenstein, D. Silver, O. Vinyals, A. W. Senior, K. Kavukcuoglu, P. Kohli and D. Hassabis (2021). "Highly accurate protein structure prediction with AlphaFold." Nature **596**(7873): 583-589.

Kovacs, J. A., J. L. Halpern, B. Lundgren, J. C. Swan, J. E. Parrillo and H. Masur (1989). "Monoclonal antibodies to Pneumocystis carinii: identification of specific antigens and characterization of antigenic differences between rat and human isolates." J Infect Dis **159**(1): 60-70.

Kovaka, S., Y. Fan, B. Ni, W. Timp and M. C. Schatz (2021). "Targeted nanopore sequencing by real-time mapping of raw electrical signal with UNCALLED." Nat Biotechnol **39**(4): 431-441.

Ma, L., Z. Chen, W. Huang da, G. Kutty, M. Ishihara, H. Wang, A. Abouelleil, L. Bishop, E. Davey, R. Deng, X. Deng, L. Fan, G. Fantoni, M. Fitzgerald, E. Gogineni, J. M. Goldberg, G. Handley, X. Hu, C. Huber, X. Jiao, K. Jones, J. Z. Levin, Y. Liu, P. Macdonald, A. Melnikov, C. Raley, M. Sassi, B. T. Sherman, X. Song, S. Sykes, B. Tran, L. Walsh, Y. Xia, J. Yang, S. Young, Q. Zeng, X. Zheng, R. Stephens, C. Nusbaum, B. W. Birren, P. Azadi, R. A. Lempicki, C. A. Cuomo and J. A. Kovacs (2016). "Genome analysis of three Pneumocystis species reveals adaptation mechanisms to life exclusively in mammalian hosts." Nat Commun **7**: 10740.

Ryckaert, J. P., G. Ciccotti and H. J. C. Berendsen (1977). "Numerical-Integration of Cartesian Equations of Motion of a System with Constraints - Molecular-Dynamics of N-Alkanes." Journal of Computational Physics **23**(3): 327-341.
Schmid-Siegert, E., S. Richard, A. Luraschi, K. Muhlethaler, M. Pagni and P. M. Hauser (2017). "Mechanisms of Surface Antigenic Variation in the Human Pathogenic Fungus Pneumocystis jirovecii." MBio **8**(6): e01470-01417.

Supplementary figure 1. Conservation of kinetochore, chromatin formation, chromatin modifying methyltransferases, DNA methylation and RNAi components in *Taphrinomycotina* and other representative fungi.

Schematic representation of centromere and a subset of associated kinetochore proteins (right). The maximum likelihood fungal phylogeny is displayed at the top with the following species: *Pneumocystis jirovecii* (Pj), *P. macacae* (Pmac), *P. canis* (Pcan), *P. oryctolagi* (Po), *P. carinii* (Pc), *P. murina* (Pm), *P. wakefieldiae* (Pwk), *Schizosaccharomyces pombe* (Spom), *Taphrina deformans* (Tapde), *Protomyces inoyeii* (Pino), *Neolecta irregularis* (Nirr), *Saitoella complicata* (Saico), *Candida albicans* (Calb), *Saccharomyces cerevisiae* (Scer), *Neurospora crassa* (Ncra), *Zymoseptoria tritici* (Ztri) and *Cryptococcus neoformans* (Cneo). Gene presence or absence profiles are represented by grew and red boxes respectively for the above species. S. *pombe* gene nomenclature is used in the matrix (https://www.pombase.org/). CTGS: cotranscriptional gene silencing. Image credit: microtubule image was modified from the original svg file available under the Creative Commons Attribution-Share Alike 4.0 International license (https://en.wikipedia.org/wiki/File:Microtubule_Structure.svg). The basic unit of chromatin structure was modified form the original file available at https://commons.wikimedia.org/wiki/File:Basic_units_of_chromatin_structure.svg.

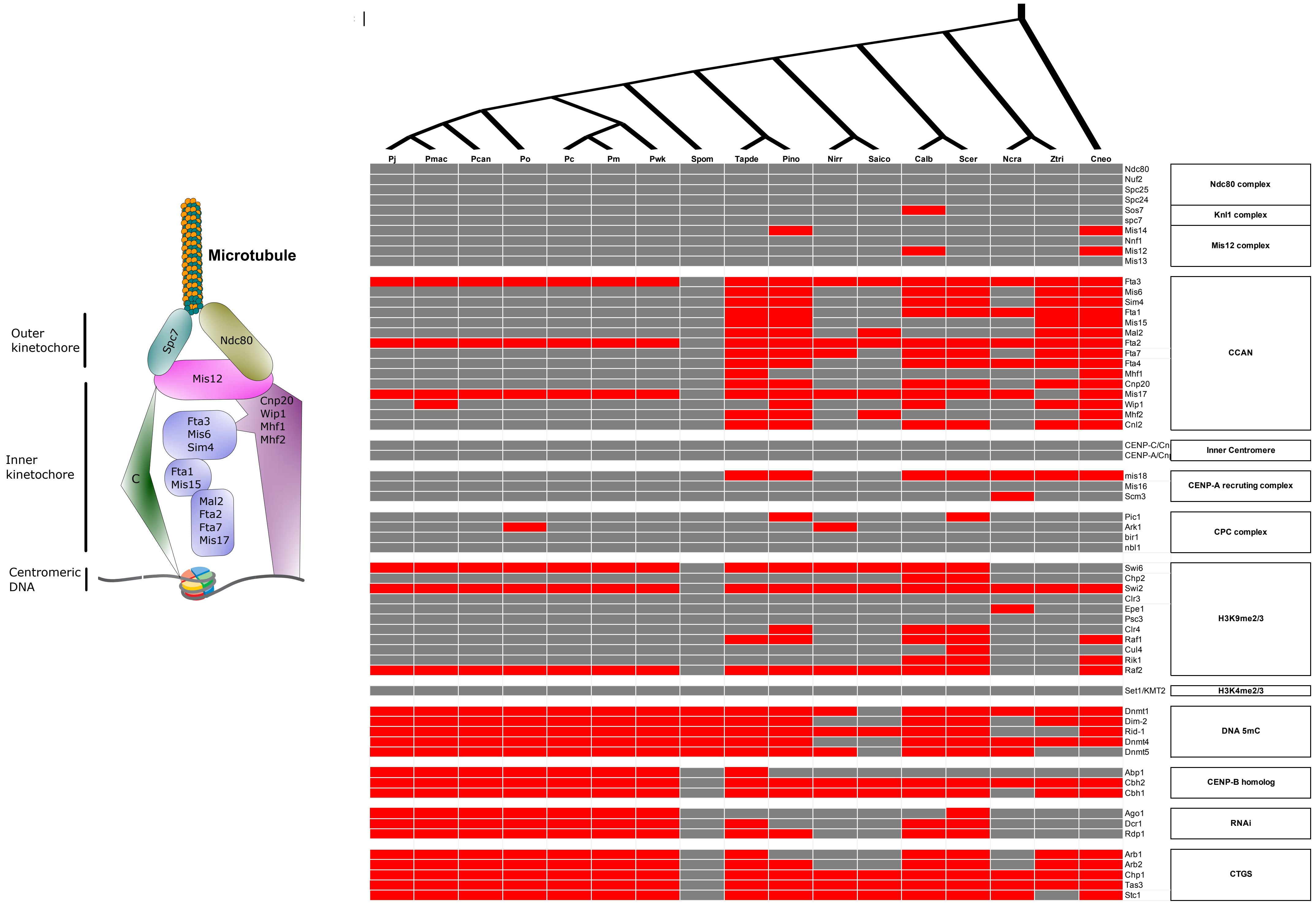
Microtubule
Outer kinetochore
Inner kinetochore
Centromeric DNA
Spc7
Ndc80
Mis12
Cnp20
Wip1
Mhf1
Mhf2
Fta3
Mis6
Sim4
Fta1
Mis15
Mal2
Fta2
Fta7
Mis17
C
Pj
Pmac
Pcan
Po
Pc
Pm
Pwk
Spom
Tapde
Pino
Nirr
Saico
Calb
Scer
Ncra
Ztri
Cneo
Ndc80
Nuf2
Spc25
Spc24
Sos7
spc7
Mis14
Nnf1
Mis12
Mis13
Fta3
Mis6
Sim4
Fta1
Mis15
Mal2
Fta2
Fta7
Fta4
Mhf1
Cnp20
Mis17
Wip1
Mhf2
Cnl2
CENP-C/Cn
CENP-A/Cn
mis18
Mis16
Scm3
Pic1
Ark1
bir1
nbl1
Swi6
Chp2
Swi2
Clr3
Epe1
Psc3
Clr4
Raf1
Cul4
Rik1
Raf2
Set1/KMT2
Dnmt1
Dim-2
Rid-1
Dnmt4
Dnmt5
Abp1
Cbh2
Cbh1
Ago1
Dcr1
Rdp1
Arb1
Arb2
Chp1
Tas3
Stc1
Ndc80 complex
Knl1 complex
Mis12 complex
CCAN
Inner Centromere
CENP-A recruting complex
CPC complex
H3K9me2/3
H3K4me2/3
DNA 5mC
CENP-B homolog
RNAi
CTGS

Supplementary figure 2. Antibody selection and testing

a-b, Selection of CENP-A and CENP-C immunogenic peptides in *Pneumocystis* CENP-A (red boxes). **c**, deepTools fingerprint analysis of initial ChIP-seq experiments. The approach determines how well the signal in the ChIP-seq sample can be differentiate from the background distribution of reads in the control sample. For factors that will enrich well defined, rather narrow regions, the resulting plot is used to assess the strength of a ChIP, but the broader the enrichment are to be expected, the less clear the plot will be. When counting the reads contained in the reads contained in 97% of all genomic binds, only 65% of the maximum number of reads are reached (i.e., *P. murina* CENP-A ChIP-seq). ChIP-seq for *P. murina* (CENP-A and CENP-C), *P. carinii* (CENP-A) displayed a restricted binding profile compared to the input (control). There is no clear difference between immunoprecipitated DNA (IP) and input in *P. carinii* CENP-C ChIP-seq as well as in *P. macacae* and *P. jirovecii* (both CENP-A and CENP-C). **d,** Selection of immunogenic peptides in *Pneumocystis macacae* Mis12. **e-f,** anti CENP-A and anti-CENP-C antibodies reactivity to *Pneumocystis* antigens measured by western blots. The inverted triangle indicates protein of interest in different blots. CENP-C present some level of cross reactivity against unidentified proteins. CENP-A and CENP-C protein locus tags are presented in parentheses (e.g., T552_00067). Anti-CENP-C antibody present some cross reactivity with unidentified proteins. Abbreviations: aa, amino acids; kDa, kilodalton.

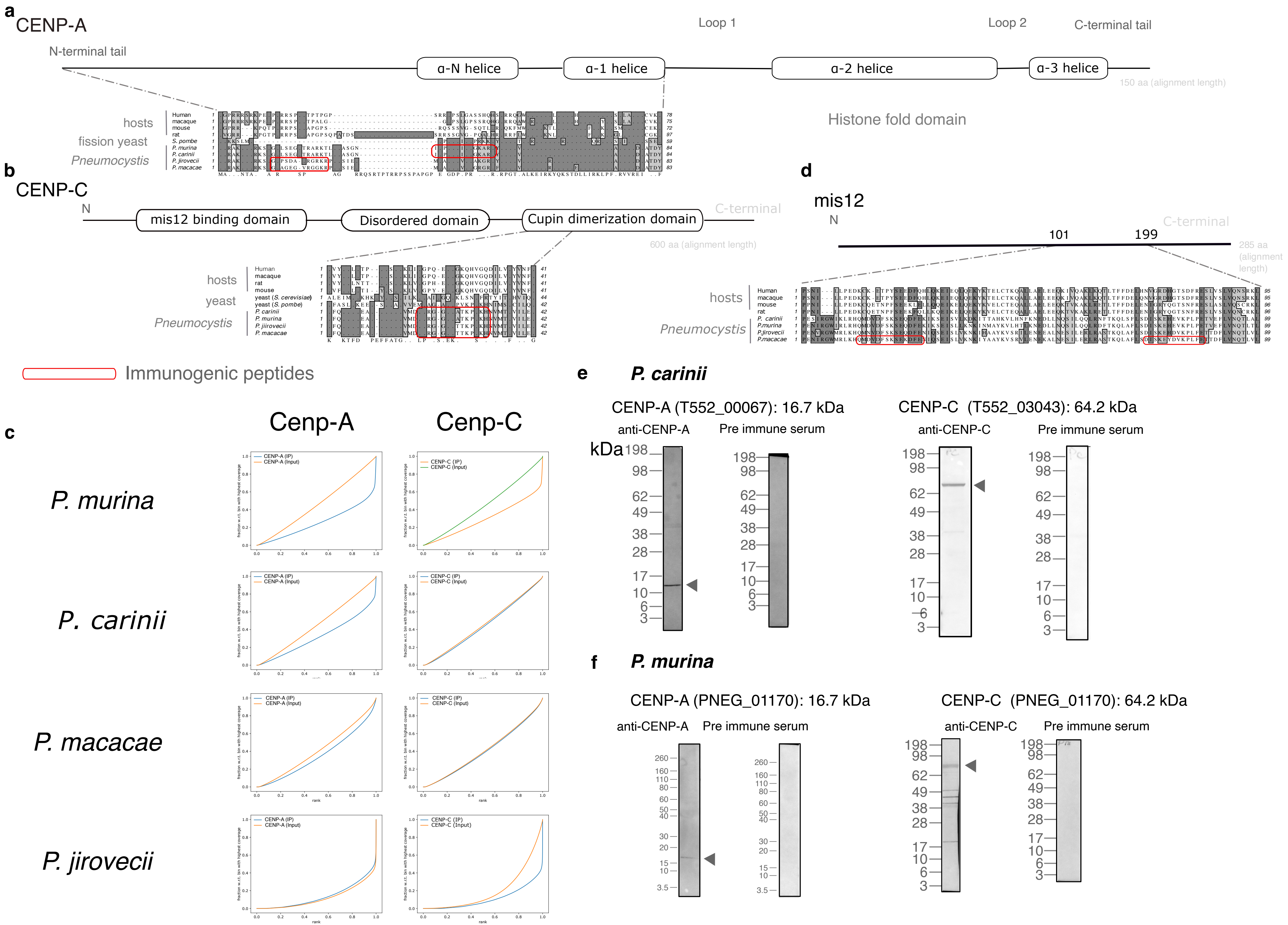

a
CENP-A
N-terminal tail
α-N helice
α-1 helice
Loop 1
α-2 helice
Loop 2
α-3 helice
C-terminal tail
150 aa (alignment length)
Histone fold domain
hosts
fission yeast
Pneumocystis
Human
macaque
mouse
rat
S. pombe
P. murina
P. carinii
P. jirovecii
P. macacae
b
CENP-C
N
mis12 binding domain
Disordered domain
Cupin dimerization domain
C-terminal
600 aa (alignment length)
hosts
yeast
Pneumocystis
Human
macaque
rat
mouse
yeast (S. cerevisiae)
yeast (S. pombe)
P. carinii
P. murina
P. jiirovecii
P. macacae
d
mis12
N
C-terminal
101
199
285 aa (alignment length)
hosts
Pneumocystis
Human
macaque
mouse
rat
P. carinii
P.murina
P.jirovecii
P.macacae
Immunogenic peptides
c
Cenp-A
Cenp-C
P. murina
P. carinii
P. macacae
P. jirovecii
CENP-A (IP)
CENP-A (Input)
CENP-C (IP)
CENP-C (Input)
rank
e
P. carinii
CENP-A (T552_00067): 16.7 kDa
anti-CENP-A
Pre immune serum
CENP-C (T552_03043): 64.2 kDa
anti-CENP-C
Pre immune serum
kDa
f
P. murina
CENP-A (PNEG_01170): 16.7 kDa
anti-CENP-A
Pre immune serum
CENP-C (PNEG_01170): 64.2 kDa
anti-CENP-C
Pre immune serum

Supplementary figure 3. *Pneumocystis* CENP-A binds to single genomic foci in replicating cells. CENP-A ChIP-seq test in *P. carinii* organisms from infected lungs and cultures at days 0, 7 and 14. CENP-A remains strongly enriched at a single genomic location up to 7 days. ChIP-seq profiles of 17 chromosomes are presented.

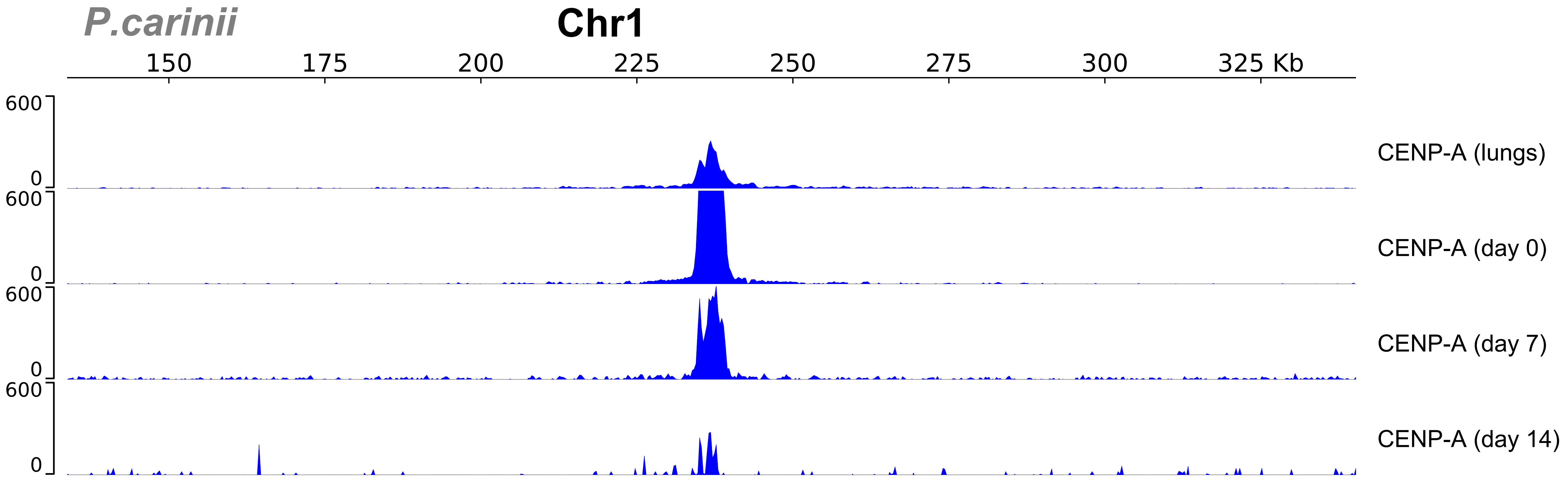

P.carinii
Chr1
150
175
200
225
250
275
300
325 Kb
600
0
CENP-A (lungs)
600
0
CENP-A (day 0)
600
0
CENP-A (day 7)
600
0
CENP-A (day 14)

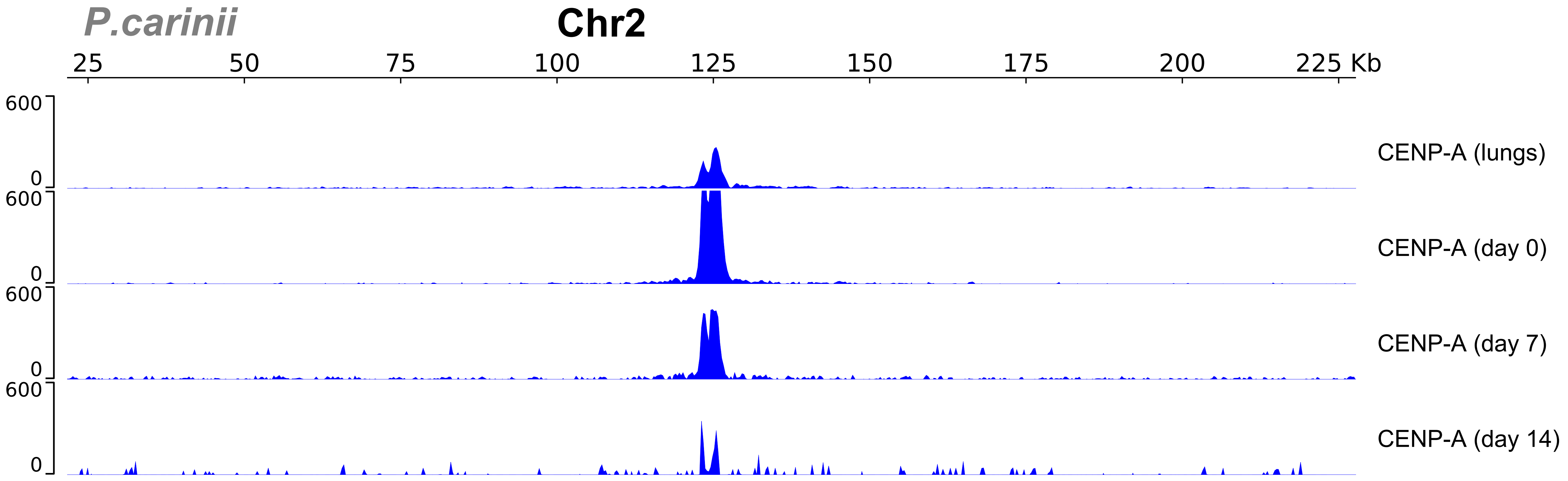

P.carinii
Chr2
25
50
75
100
125
150
175
200
225 Kb
600
0
CENP-A (lungs)
600
0
CENP-A (day 0)
600
0
CENP-A (day 7)
600
0
CENP-A (day 14)

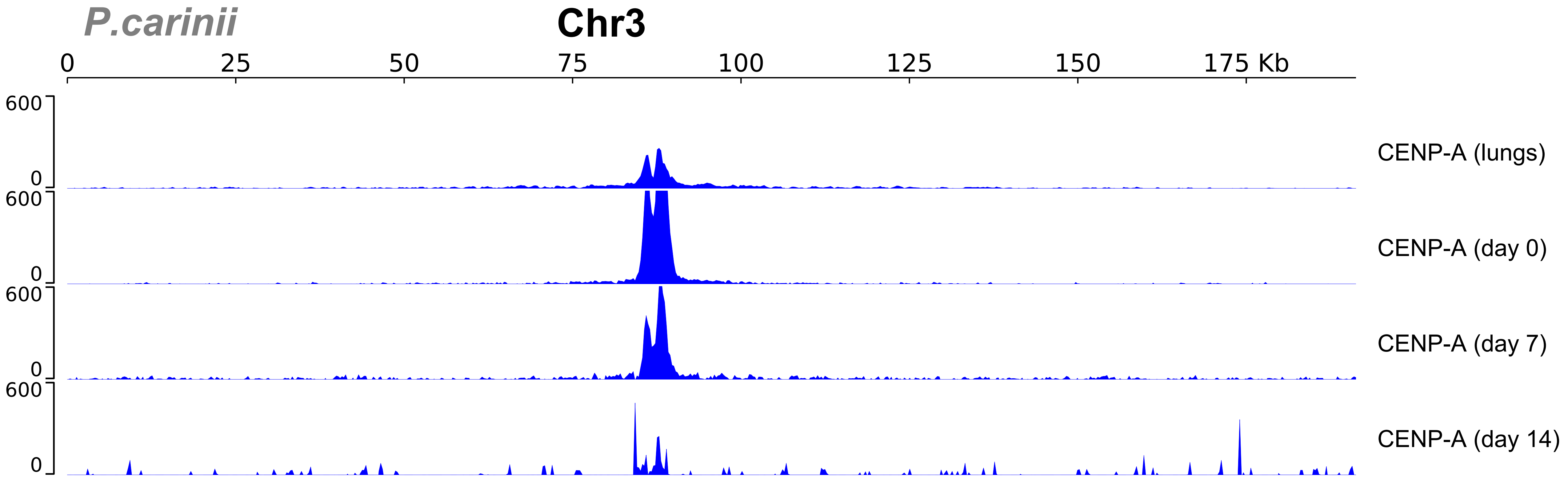
P.carinii
Chr3
0
25
50
75
100
125
150
175 Kb
600
0
CENP-A (lungs)
CENP-A (day 0)
CENP-A (day 7)
CENP-A (day 14)

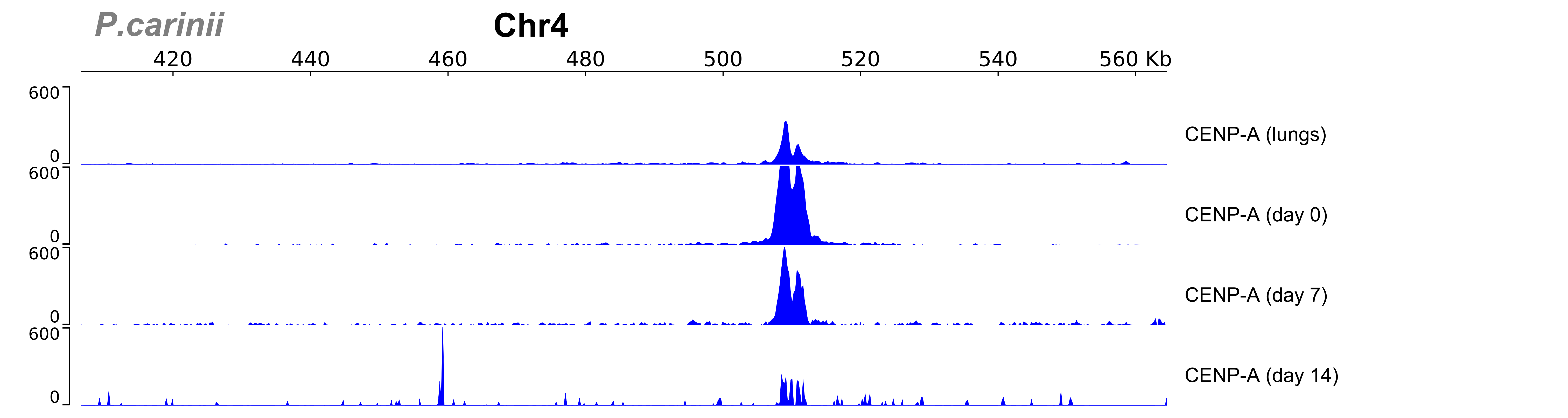

P.carinii
Chr4
420
440
460
480
500
520
540
560 Kb
600
0
CENP-A (lungs)
600
0
CENP-A (day 0)
600
0
CENP-A (day 7)
600
0
CENP-A (day 14)

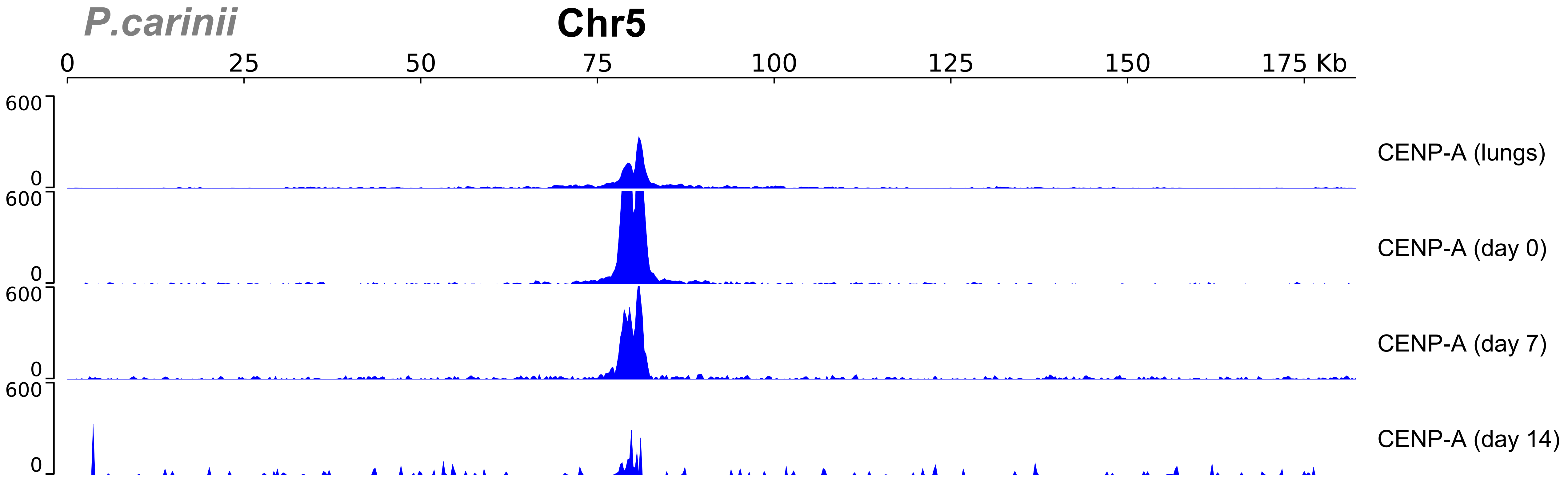
P.carinii
Chr5
0
25
50
75
100
125
150
175 Kb
600
0
CENP-A (lungs)
CENP-A (day 0)
CENP-A (day 7)
CENP-A (day 14)

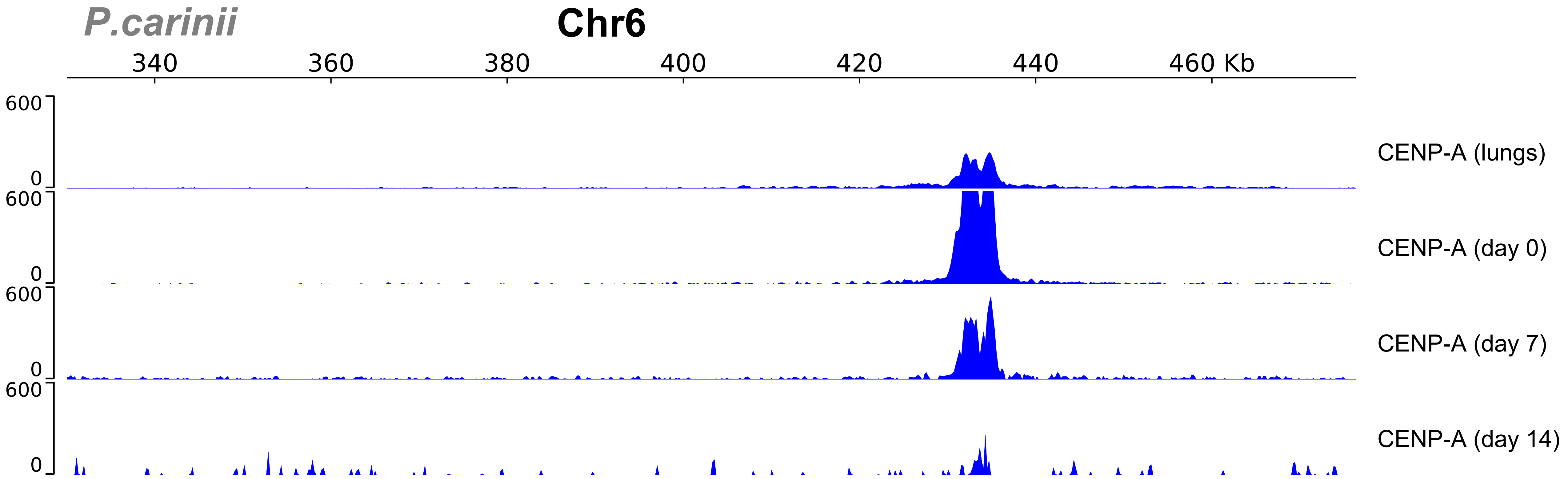
P.carinii
Chr6
340
360
380
400
420
440
460 Kb
600
0
CENP-A (lungs)
600
0
CENP-A (day 0)
600
0
CENP-A (day 7)
600
0
CENP-A (day 14)

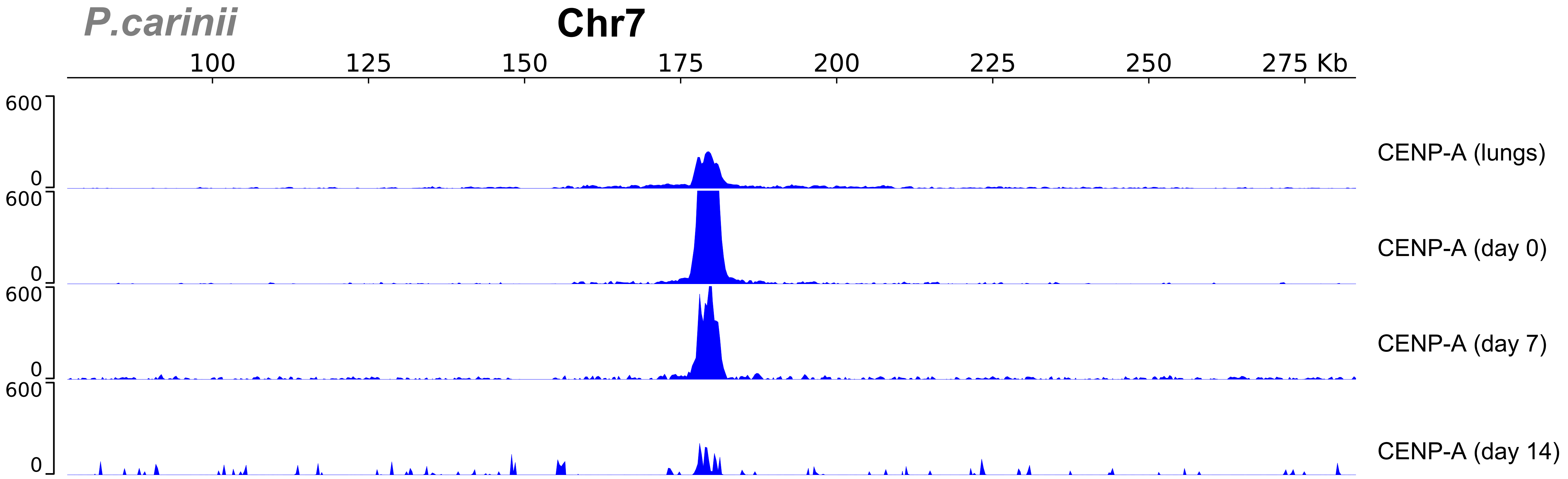

P.carinii
Chr7
100
125
150
175
200
225
250
275 Kb
600
0
CENP-A (lungs)
600
0
CENP-A (day 0)
600
0
CENP-A (day 7)
600
0
CENP-A (day 14)

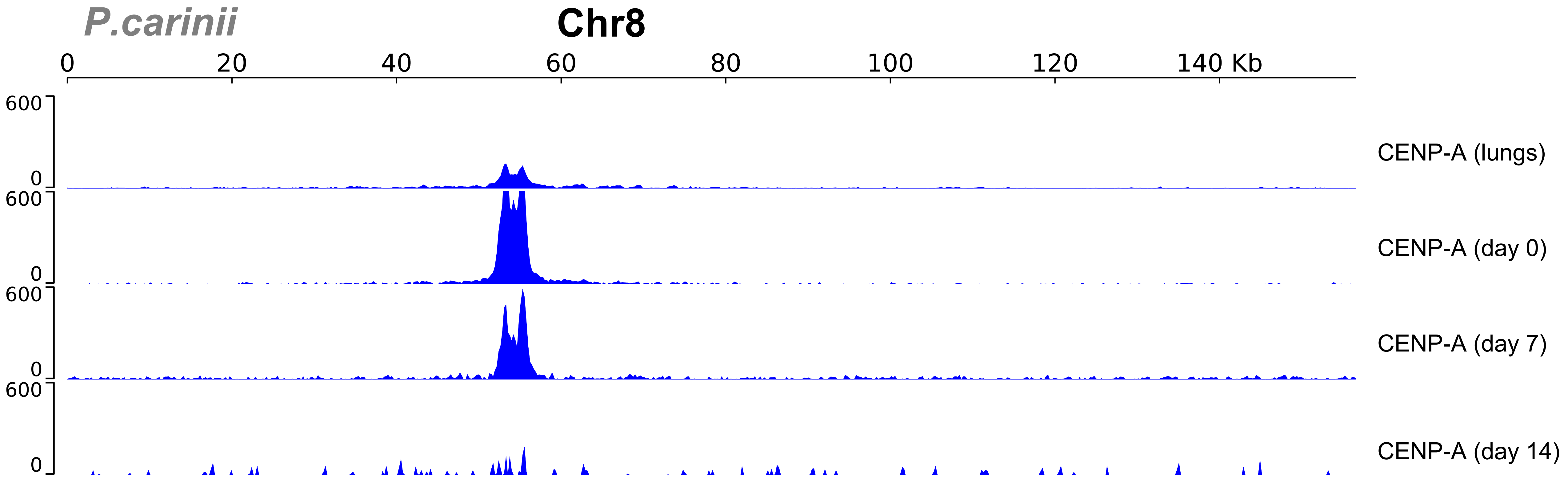

P.carinii
Chr8
0
20
40
60
80
100
120
140 Kb
600
0
CENP-A (lungs)
600
0
CENP-A (day 0)
600
0
CENP-A (day 7)
600
0
CENP-A (day 14)

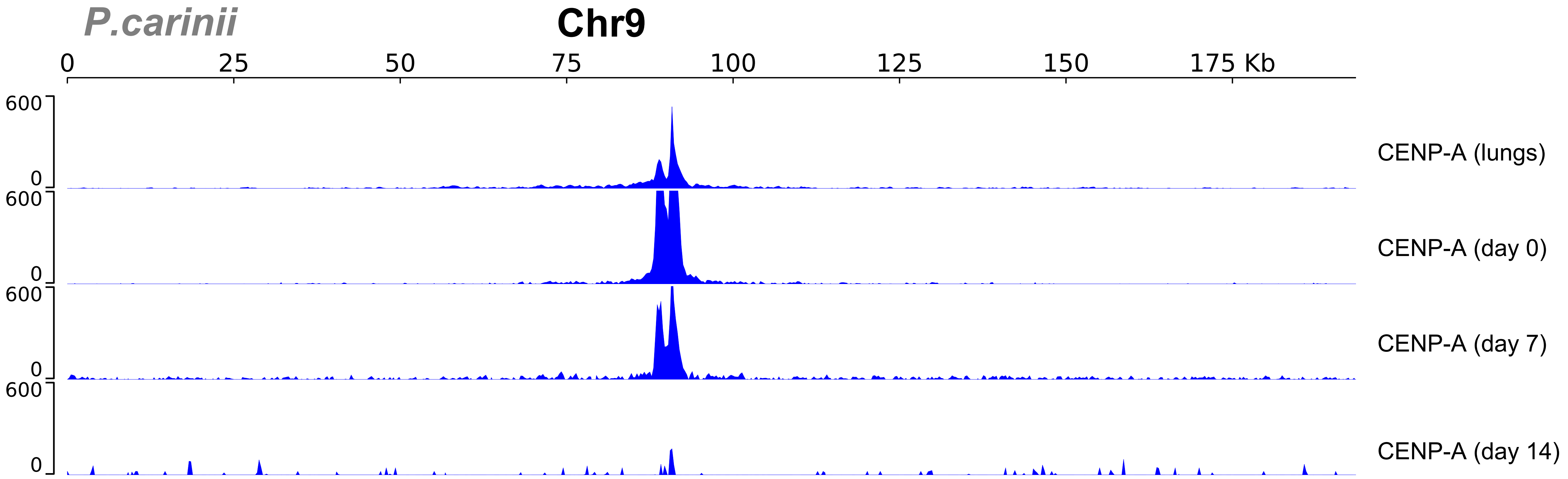
P.carinii
Chr9
0
25
50
75
100
125
150
175 Kb
600
0
CENP-A (lungs)
600
0
CENP-A (day 0)
600
0
CENP-A (day 7)
600
0
CENP-A (day 14)

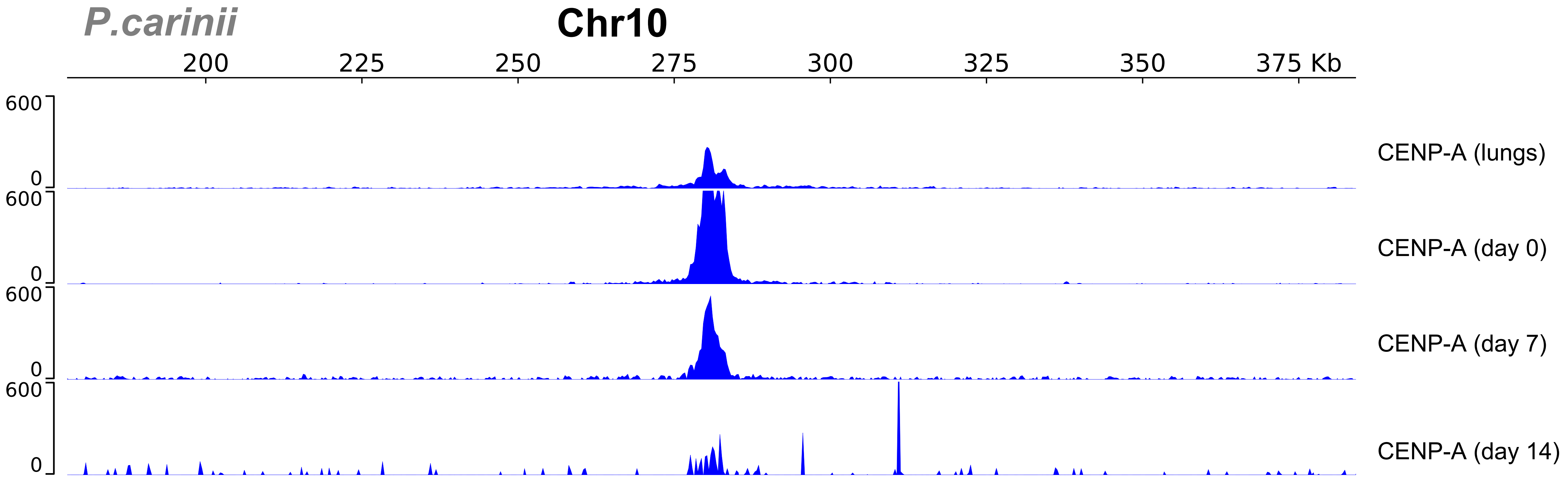

P.carinii
Chr10
200
225
250
275
300
325
350
375 Kb
600
0
CENP-A (lungs)
600
0
CENP-A (day 0)
600
0
CENP-A (day 7)
600
0
CENP-A (day 14)

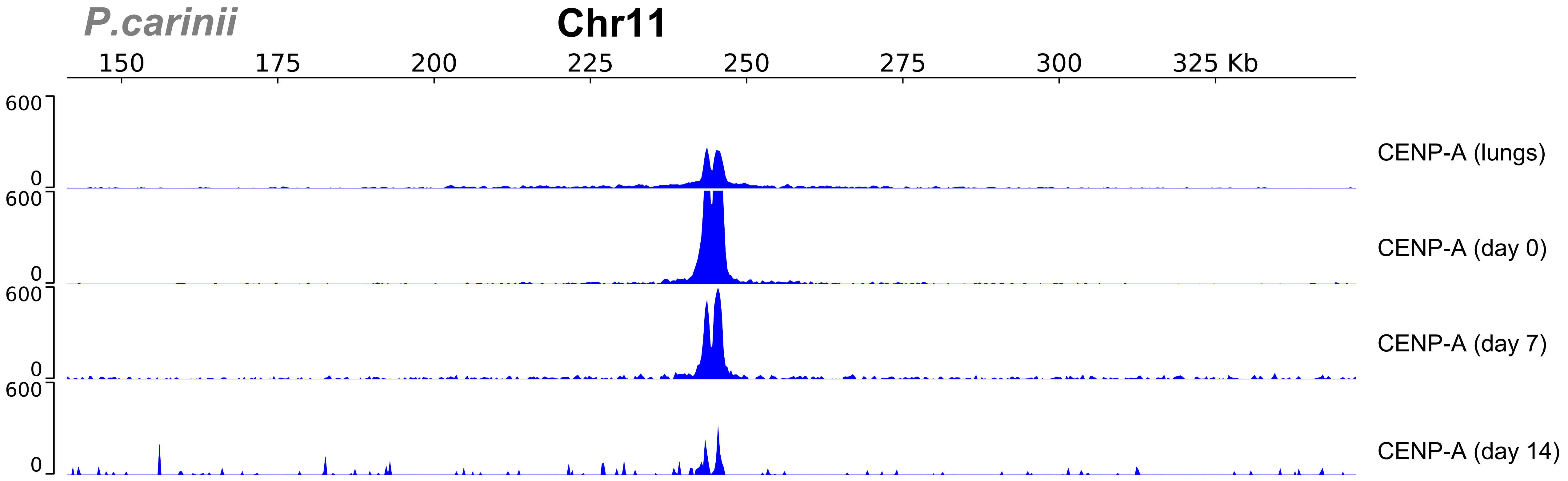
P.carinii
Chr11
150
175
200
225
250
275
300
325 Kb
600
0
CENP-A (lungs)
600
0
CENP-A (day 0)
600
0
CENP-A (day 7)
600
0
CENP-A (day 14)

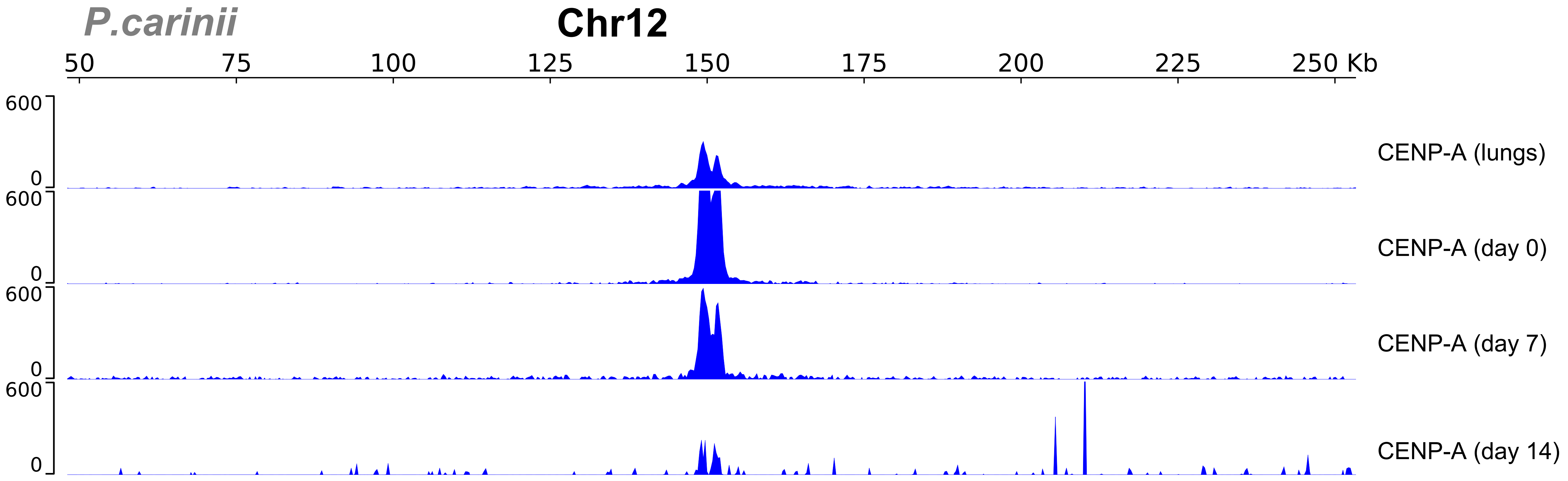

P.carinii
Chr12
50
75
100
125
150
175
200
225
250 Kb
600
0
CENP-A (lungs)
600
0
CENP-A (day 0)
600
0
CENP-A (day 7)
600
0
CENP-A (day 14)

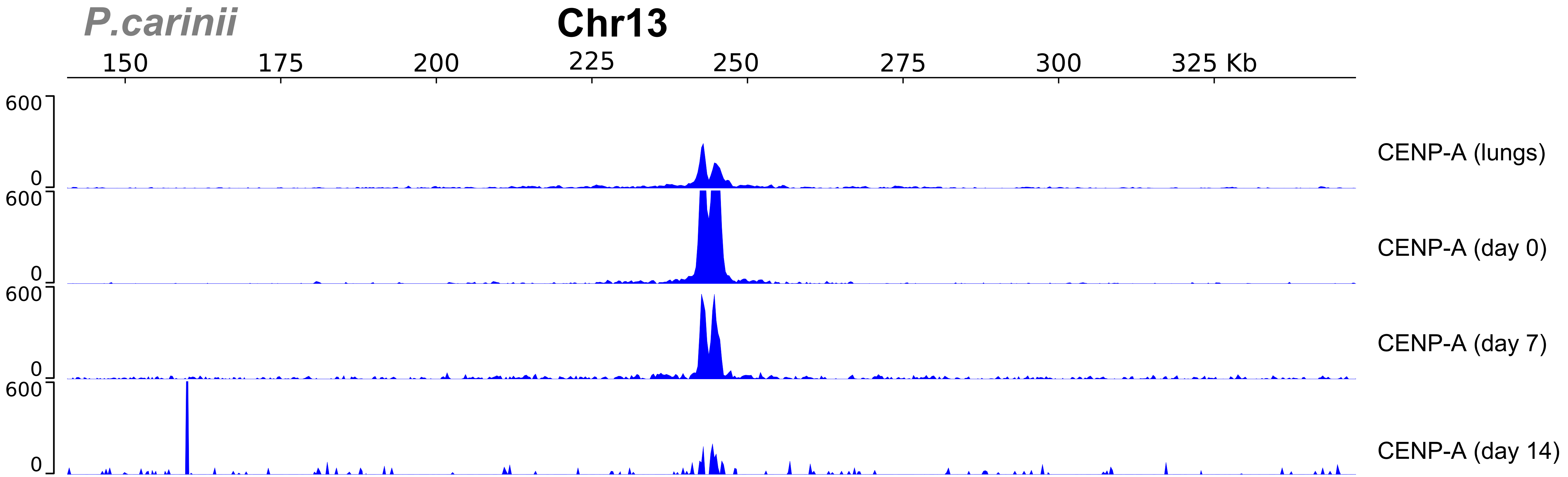

P.carinii
Chr13
150
175
200
225
250
275
300
325 Kb
600
0
CENP-A (lungs)
600
0
CENP-A (day 0)
600
0
CENP-A (day 7)
600
0
CENP-A (day 14)

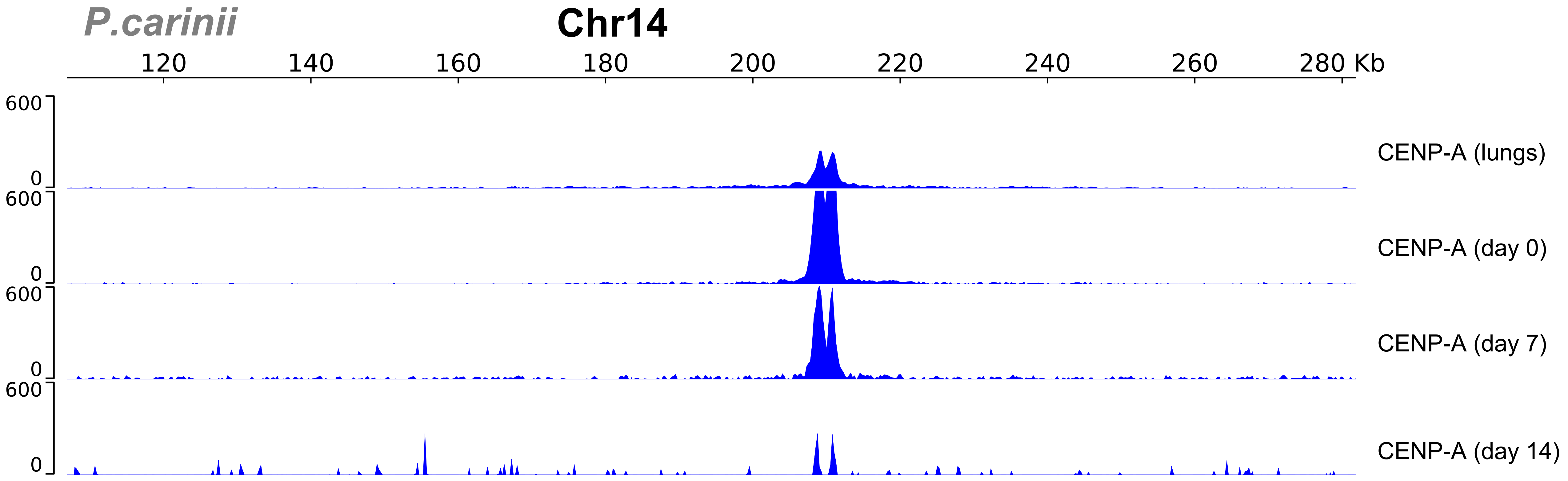
P.carinii
Chr14
120
140
160
180
200
220
240
260
280 Kb
600
0
CENP-A (lungs)
CENP-A (day 0)
CENP-A (day 7)
CENP-A (day 14)

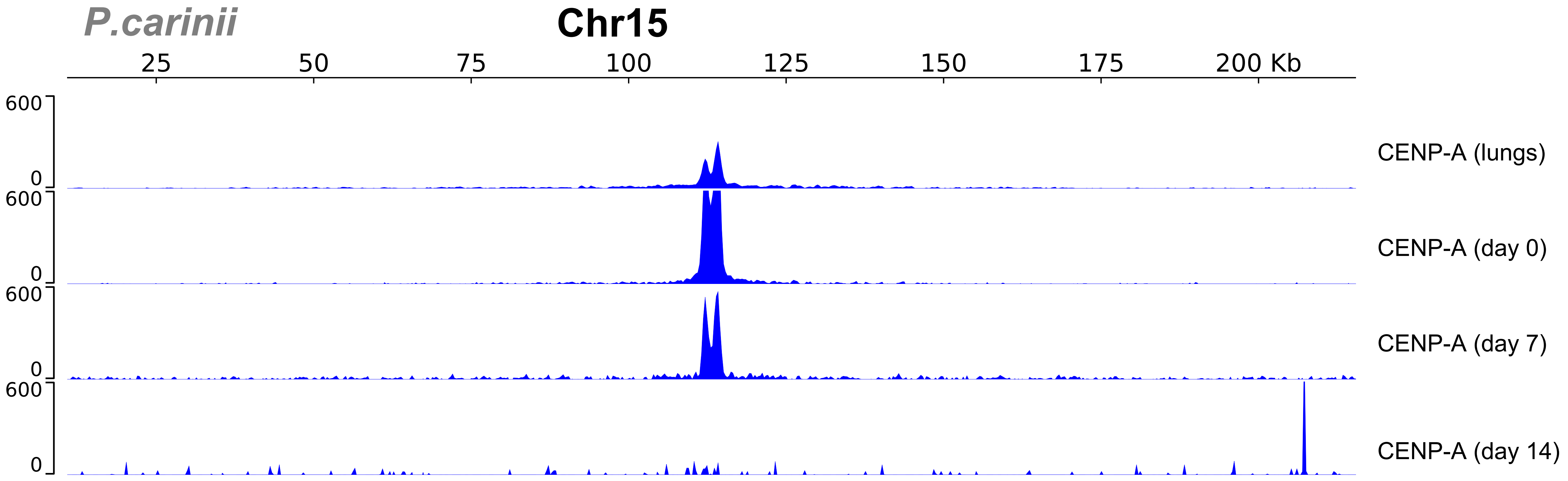
P.carinii
Chr15
25
50
75
100
125
150
175
200 Kb
600
0
CENP-A (lungs)
600
0
CENP-A (day 0)
600
0
CENP-A (day 7)
600
0
CENP-A (day 14)

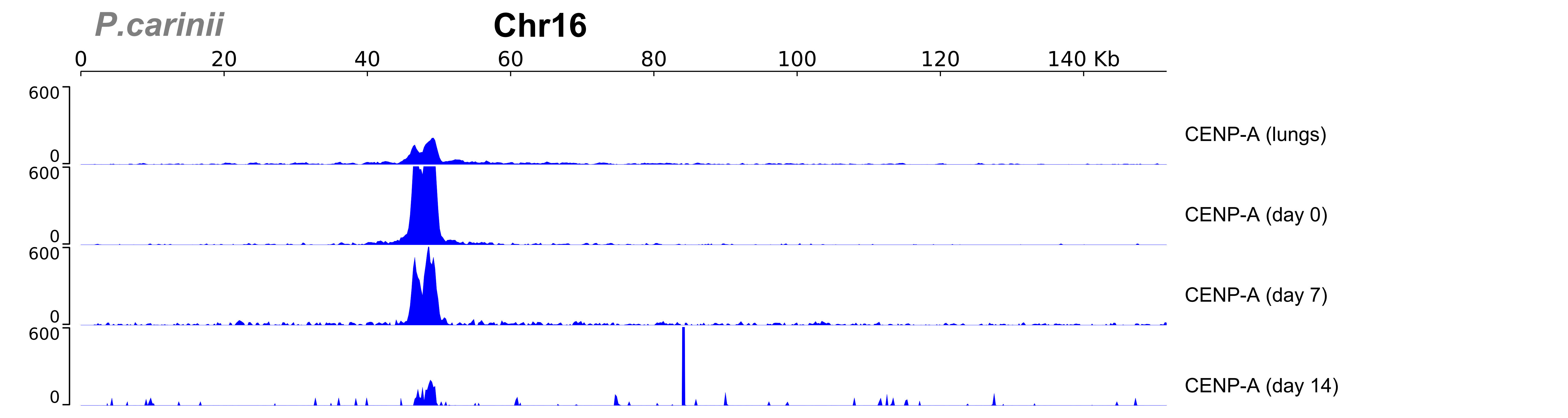
P.carinii
Chr16
0
20
40
60
80
100
120
140 Kb
600
0
CENP-A (lungs)
600
0
CENP-A (day 0)
600
0
CENP-A (day 7)
600
0
CENP-A (day 14)

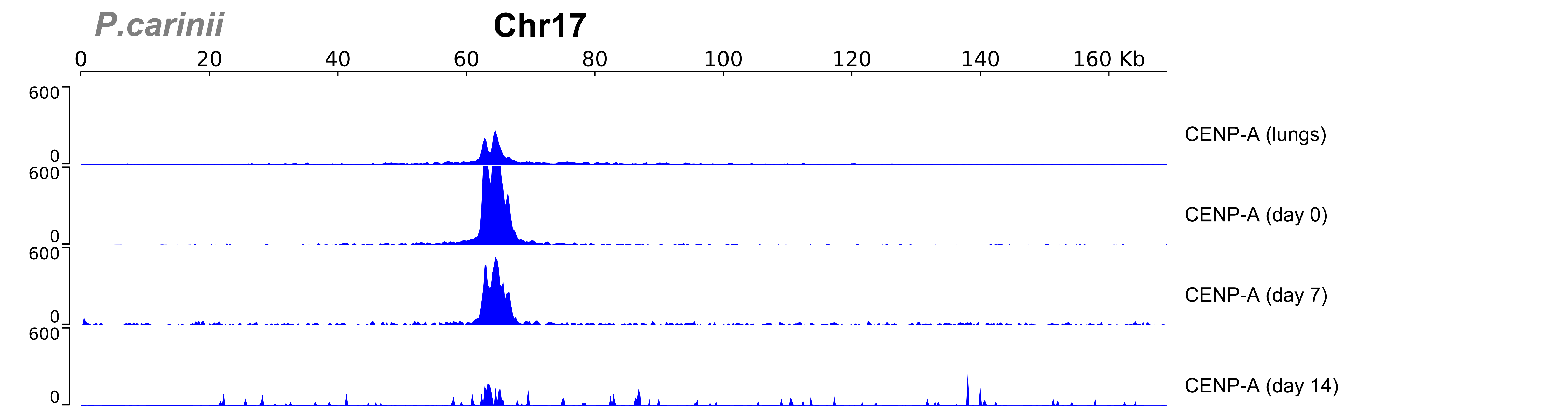

P.carinii
Chr17
0
20
40
60
80
100
120
140
160 Kb
600
0
CENP-A (lungs)
600
0
CENP-A (day 0)
600
0
CENP-A (day 7)
600
0
CENP-A (day 14)

Supplementary figure 4. CENP-A enrichment at *Pneumocystis* centromeres by ChIP-qPCR. Formalin fixed chromatins were prepared from *P. murina* and *P. carinii* organisms cultured on coculture of A549 and Let-1 cell line for 18 days, followed by immunoprecipitation with anti *pn*CENP-A antibody and DNA extraction. Only cultured *Pneumocystis* species are presented. ChIP-qPCR values are determined from three technical replicates. The percent input values of centromeres (Cen) are compared to a non-centromeric control region selected 25 kb away from the centromere 1 in each species (far-Cen1). Three independent experiments were performed for reproducibility and the result of a representative experiment is presented. Error bars represent the standard errors of the mean. Statistical analyses were performed using one-Way ANOVA and pairwise comparisons were performed using unpaired t-test; ****, $P < 0.0001$; *** $P < 0.001$; ns, not significant). Top, *pn*CENP-A enrichment is significant in 16 of 17 centromeres in *P. murina*. Bottom, *pn*CENP-A is significantly enriched in all *P. carinii* centromeres.

P. murina

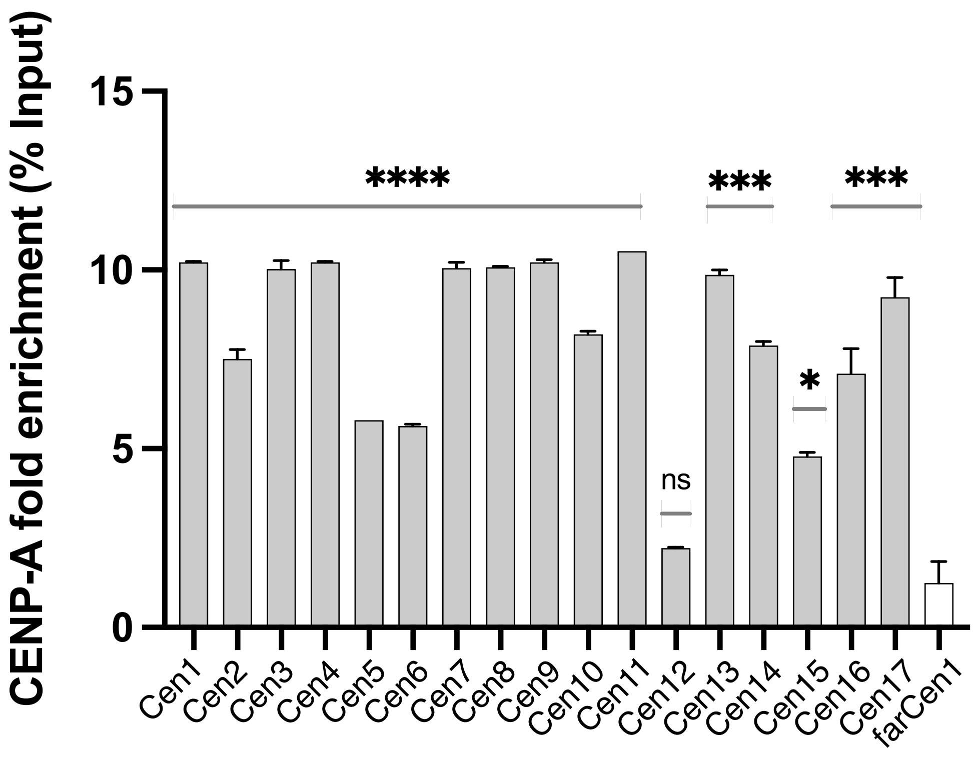

P. carinii

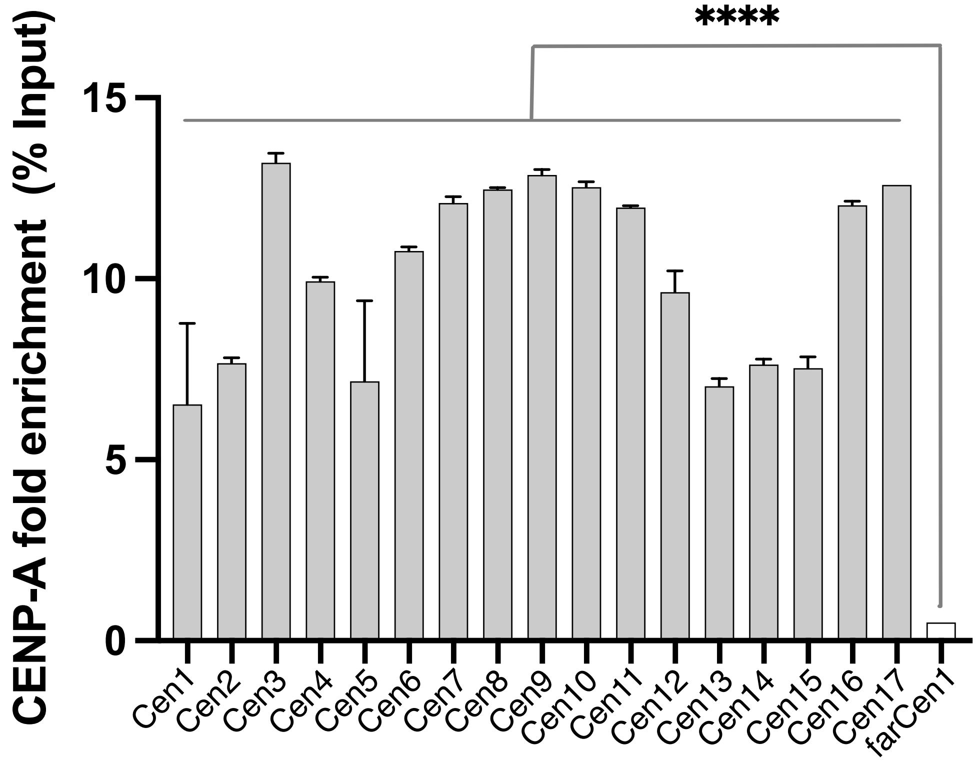

Supplementary figure 5. Genomic view of *P. macacae* genome showing pericentromeric heterochromatin.

Genomic views of *P. macacae* genomic scaffolds subsequently showing annotated genes (directed grey boxes), repeats (DNA transposons and retrotransposons), percent GC content (blue), ChIP-seq read coverage distribution (BPM normalized over bins of 50 bp; input subtracted) of CENP-A, CENP-C, histones H3 and H4 ratio, heterochromatin-associated modifications (H3K9me2 and H3K9me3), euchromatin (H3K4me2) and gene expression (RNA-seq) in relation with centromeres.

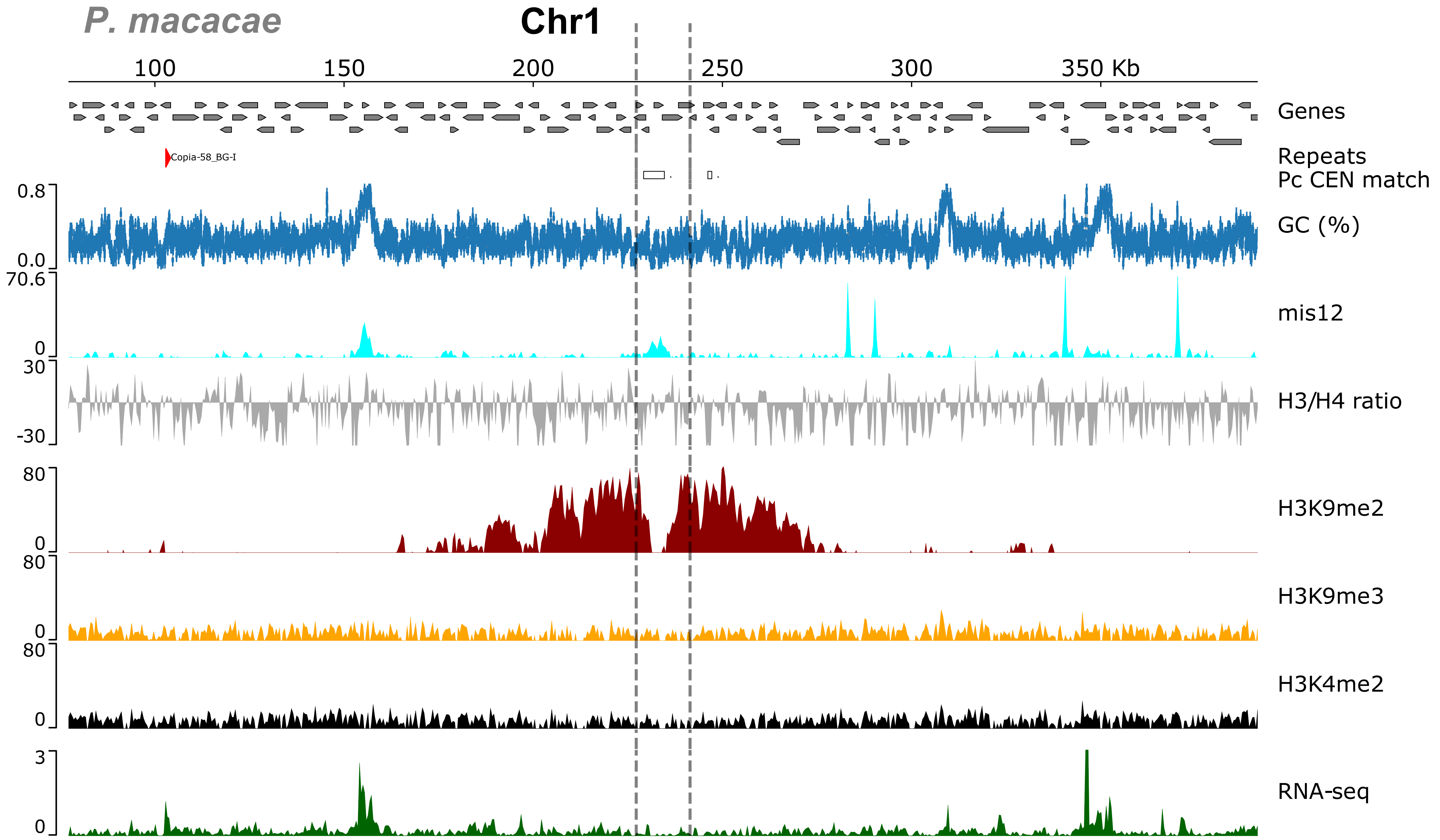

P. macacae
Chr1
100
150
200
250
300
350 Kb
Genes
Repeats
Copia-58_BG-I
Pc CEN match
0.8
0.0
GC (%)
70.6
0
mis12
30
-30
H3/H4 ratio
80
0
H3K9me2
80
0
H3K9me3
80
0
H3K4me2
3
0
RNA-seq

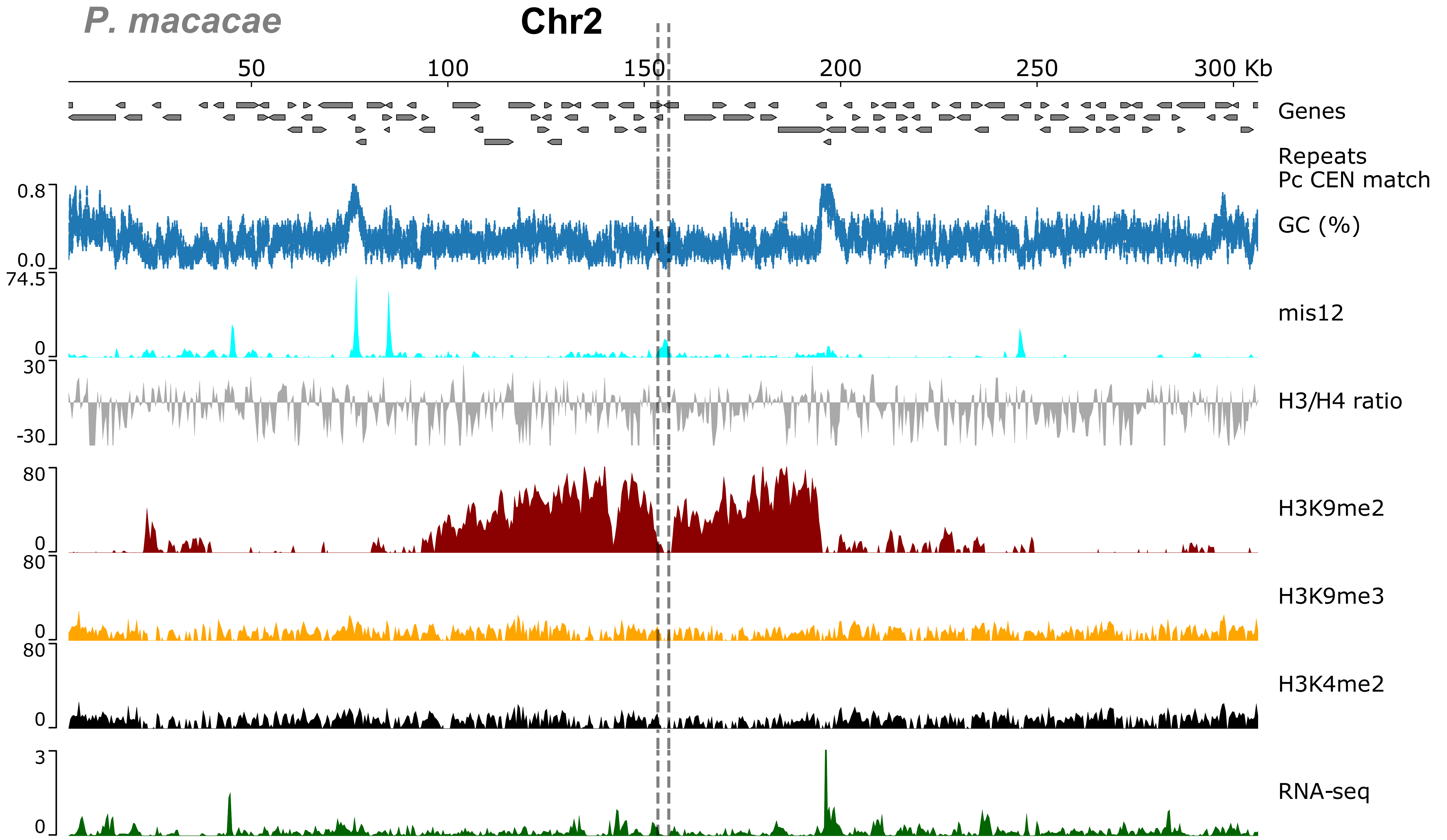
P. macacae
Chr2
50
100
150
200
250
300 Kb
Genes
Repeats
Pc CEN match
0.8
0.0
GC (%)
74.5
0
mis12
30
-30
H3/H4 ratio
80
0
H3K9me2
80
0
H3K9me3
80
0
H3K4me2
3
0
RNA-seq

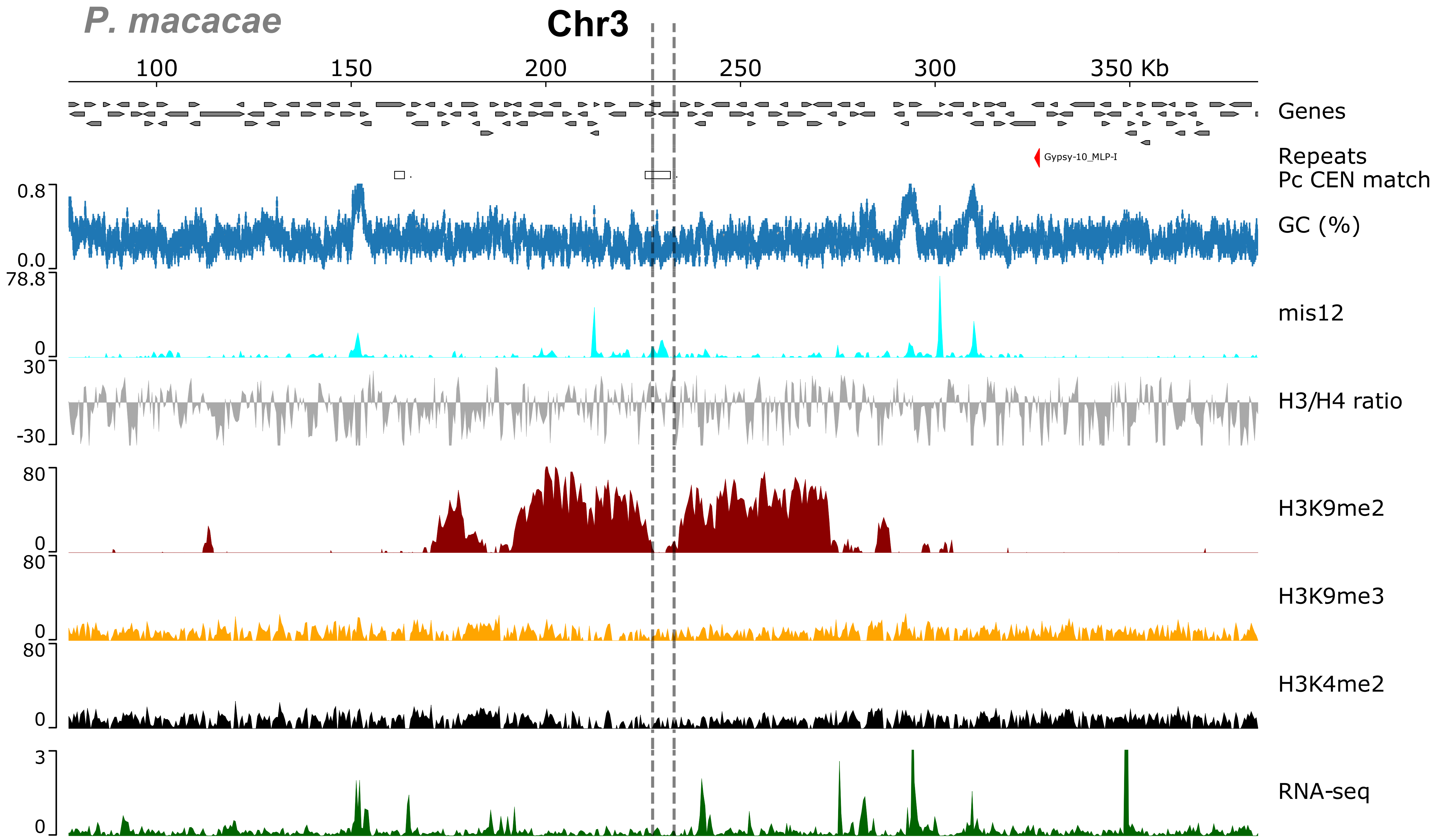
P. macacae
Chr3
100
150
200
250
300
350 Kb
Genes
Repeats
Gypsy-10_MLP-I
Pc CEN match
0.8
0.0
GC (%)
78.8
0
mis12
30
-30
H3/H4 ratio
80
0
H3K9me2
80
0
H3K9me3
80
0
H3K4me2
3
0
RNA-seq

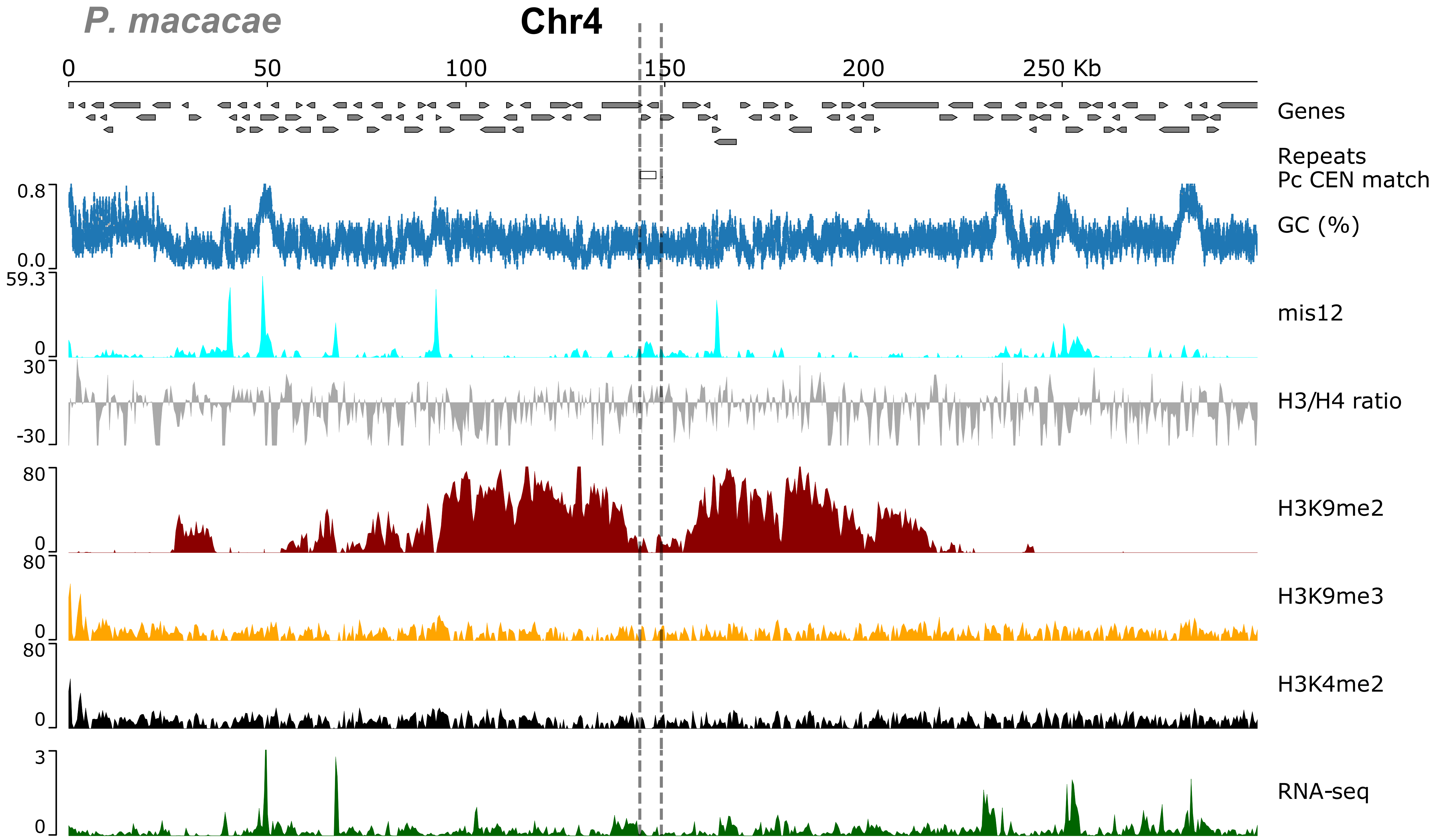

P. macacae
Chr4
0
50
100
150
200
250 Kb
Genes
Repeats
Pc CEN match
0.8
0.0
GC (%)
59.3
0
mis12
30
-30
H3/H4 ratio
80
0
H3K9me2
80
0
H3K9me3
80
0
H3K4me2
3
0
RNA-seq

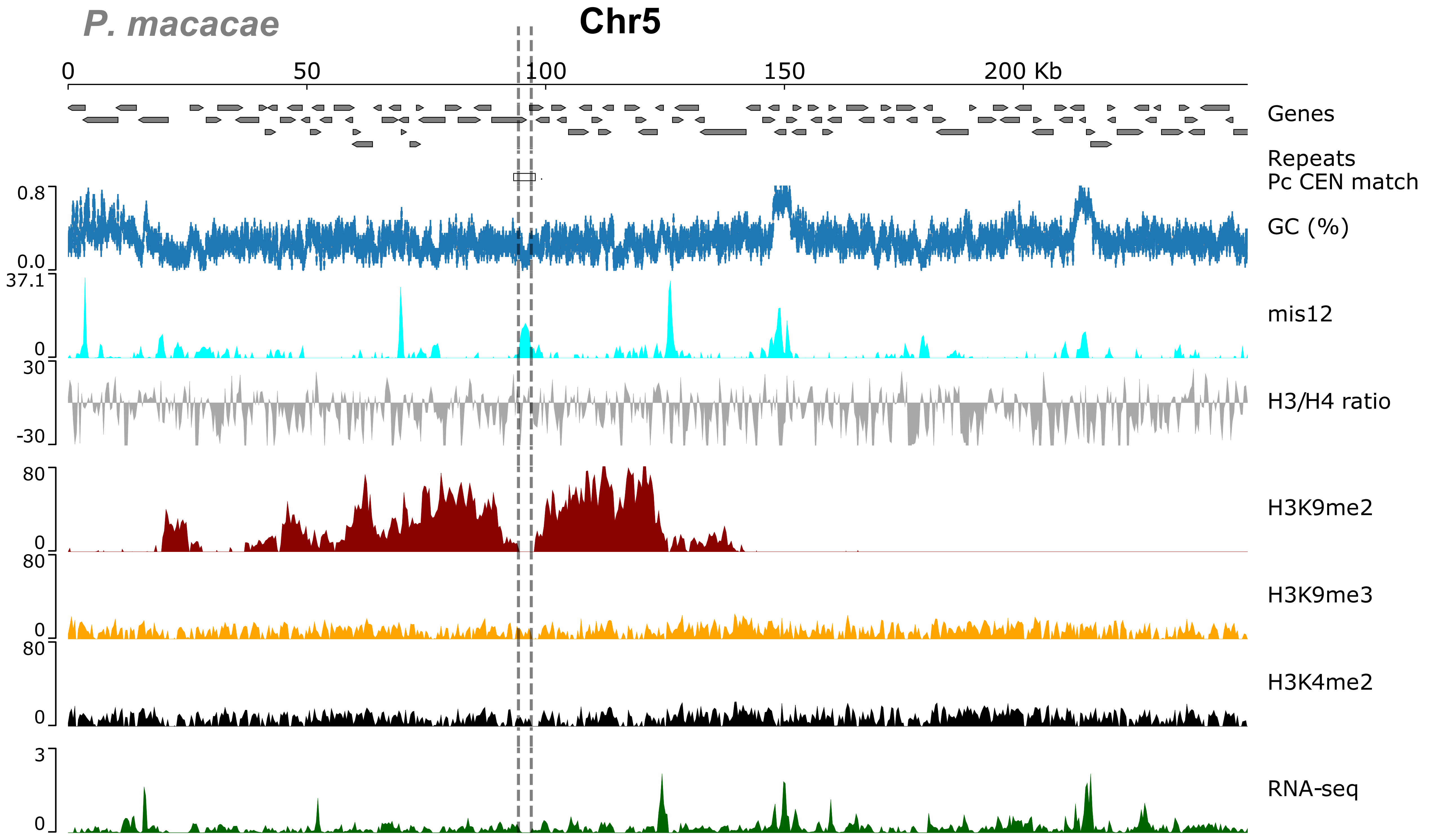

P. macacae
Chr5
0
50
100
150
200 Kb
Genes
Repeats
Pc CEN match
0.8
0.0
GC (%)
37.1
0
mis12
30
-30
H3/H4 ratio
80
0
H3K9me2
80
0
H3K9me3
80
0
H3K4me2
3
0
RNA-seq

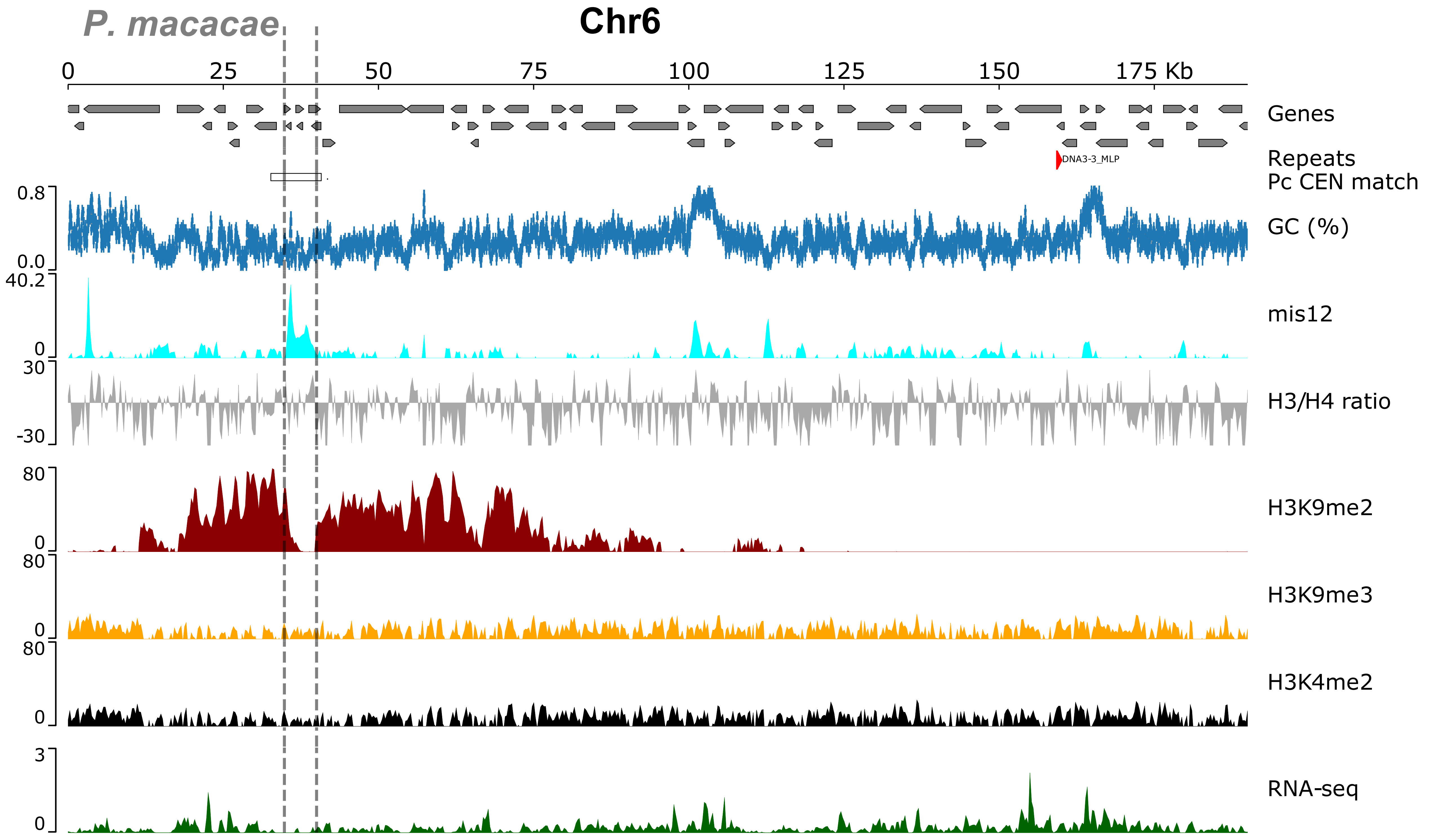
P. macacae
Chr6
0
25
50
75
100
125
150
175 Kb
Genes
Repeats
DNA3-3_MLP
Pc CEN match
0.8
0.0
GC (%)
40.2
0
mis12
30
-30
H3/H4 ratio
80
0
H3K9me2
80
0
H3K9me3
80
0
H3K4me2
3
0
RNA-seq

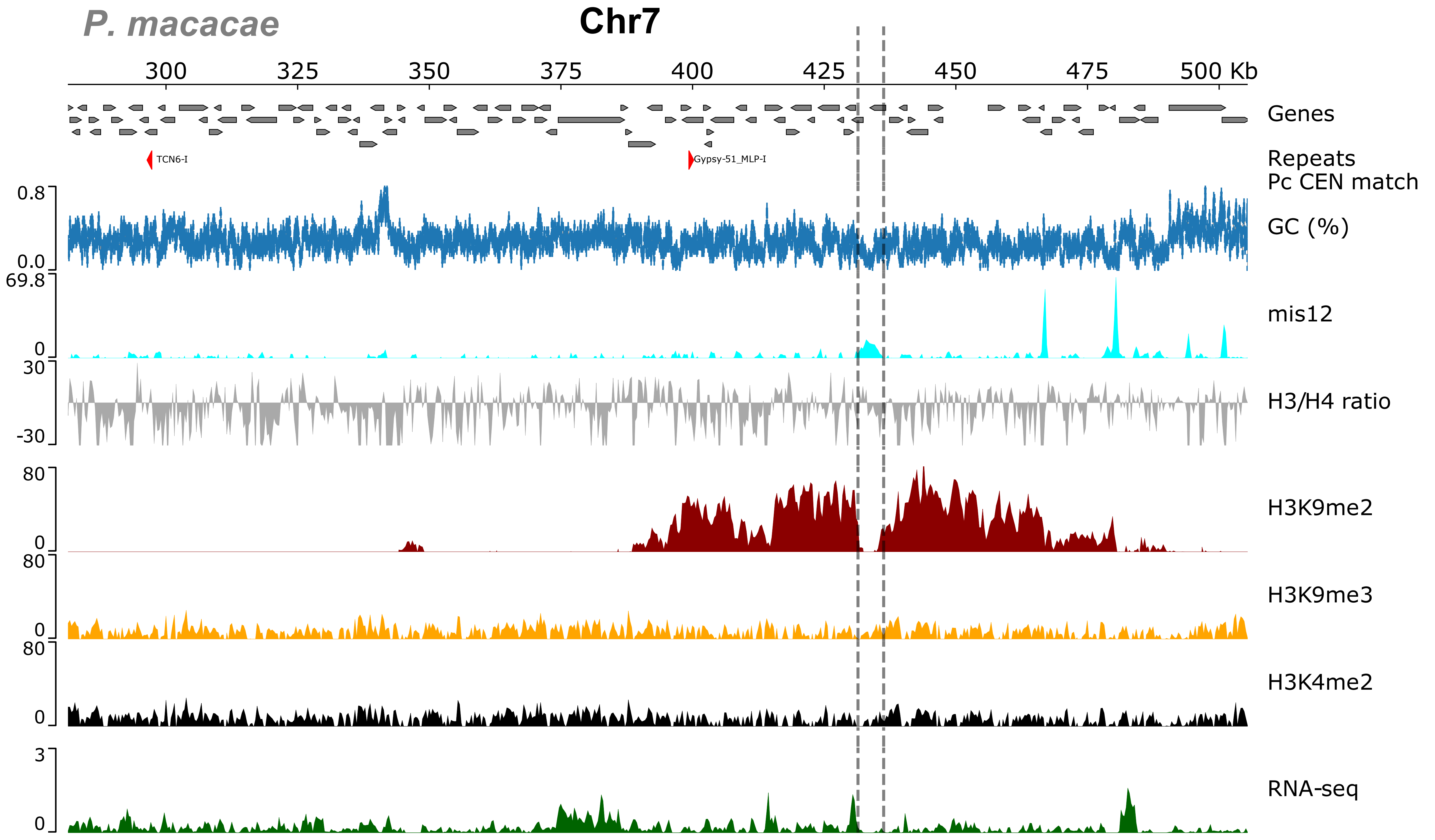
P. macacae
Chr7
300
325
350
375
400
425
450
475
500 Kb
Genes
TCN6-I
Gypsy-51_MLP-I
Repeats
Pc CEN match
0.8
0.0
GC (%)
69.8
0
mis12
30
-30
H3/H4 ratio
80
0
H3K9me2
80
0
H3K9me3
80
0
H3K4me2
3
0
RNA-seq

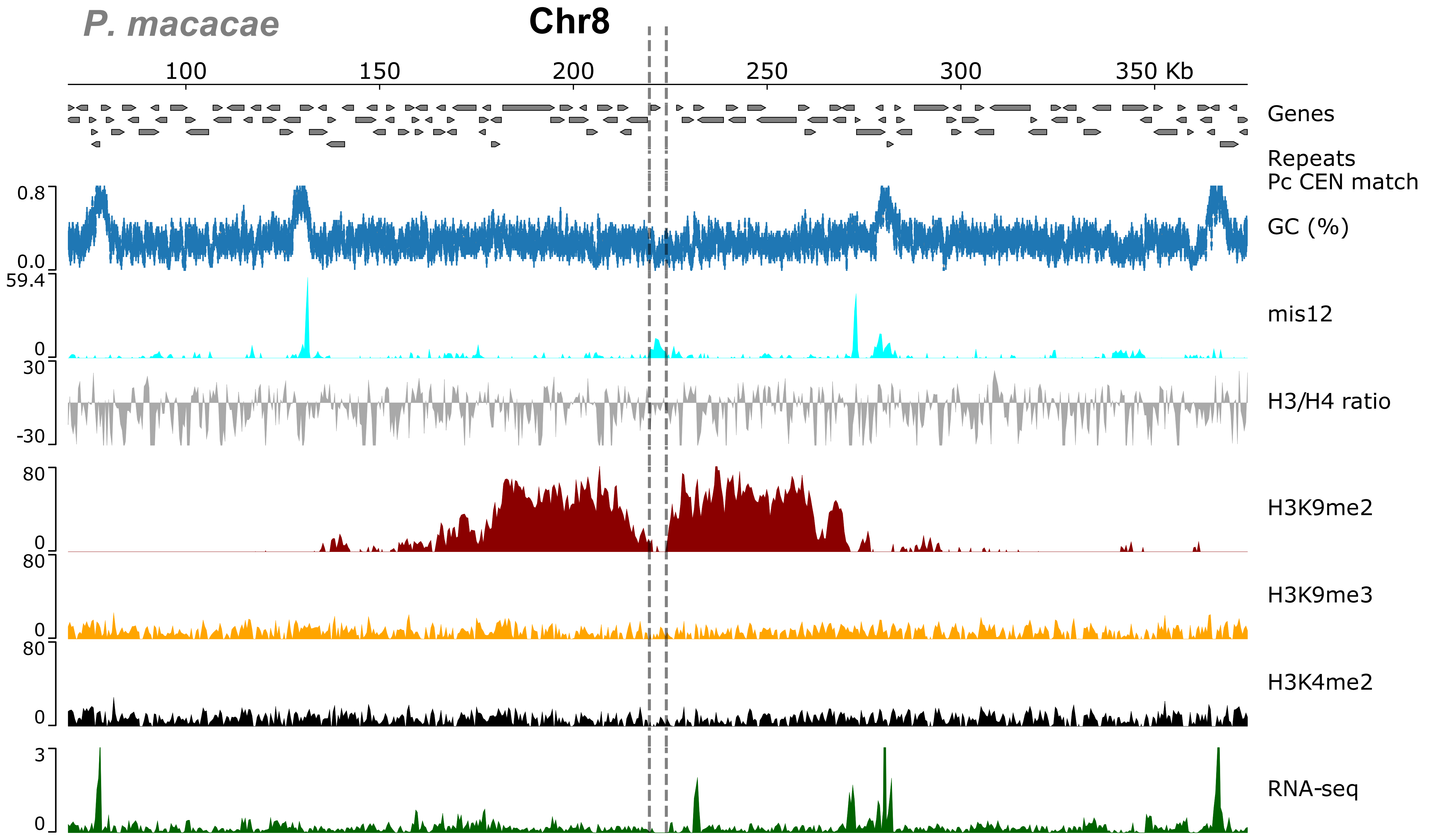

P. macacae
Chr8
100
150
200
250
300
350 Kb
Genes
Repeats
Pc CEN match
0.8
0.0
GC (%)
59.4
0
mis12
30
-30
H3/H4 ratio
80
0
H3K9me2
80
0
H3K9me3
80
0
H3K4me2
3
0
RNA-seq

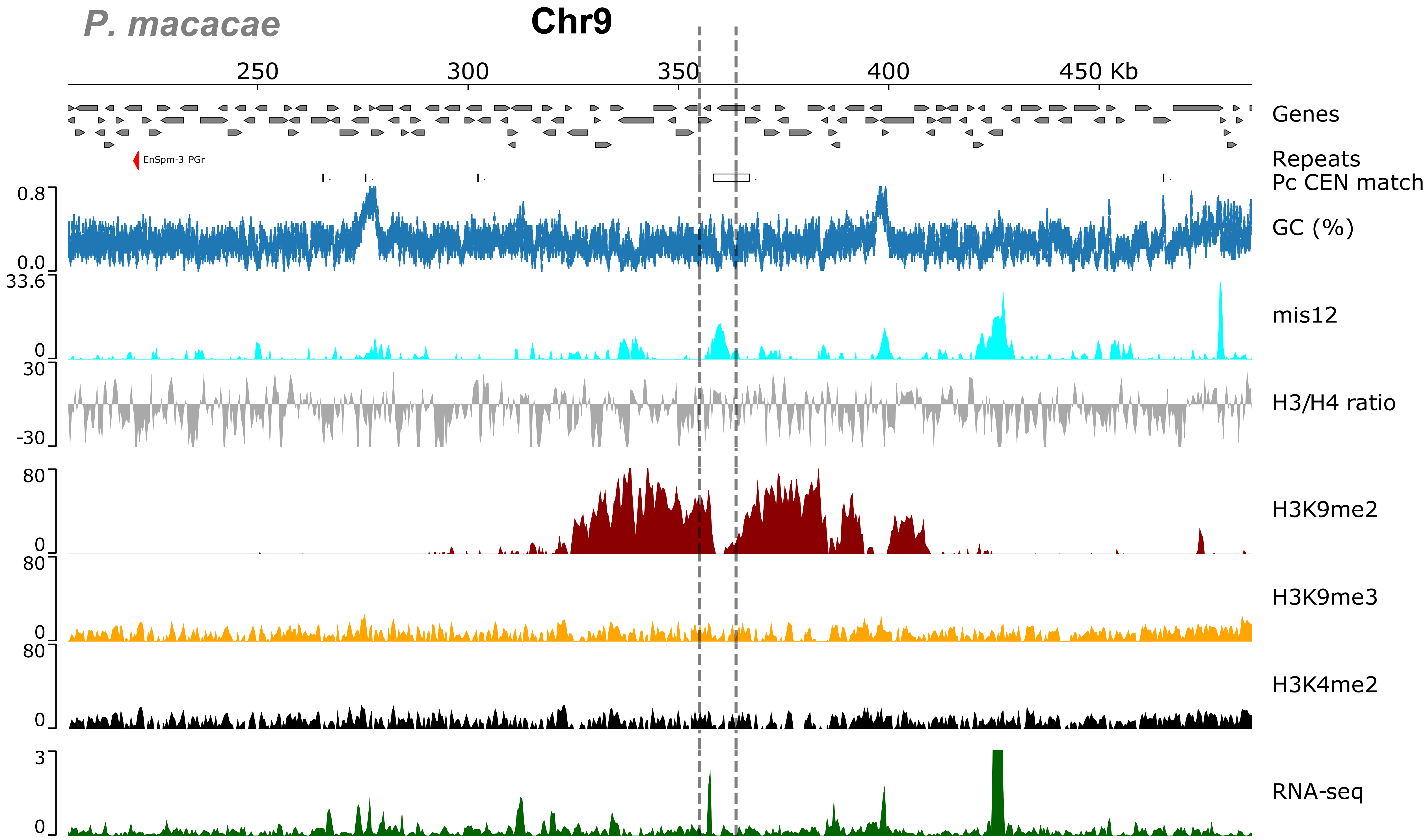
P. macacae
Chr9
250
300
350
400
450 Kb
Genes
EnSpm-3_PGr
Repeats
Pc CEN match
0.8
0.0
GC (%)
33.6
0
mis12
30
-30
H3/H4 ratio
80
0
H3K9me2
80
0
H3K9me3
80
0
H3K4me2
3
0
RNA-seq

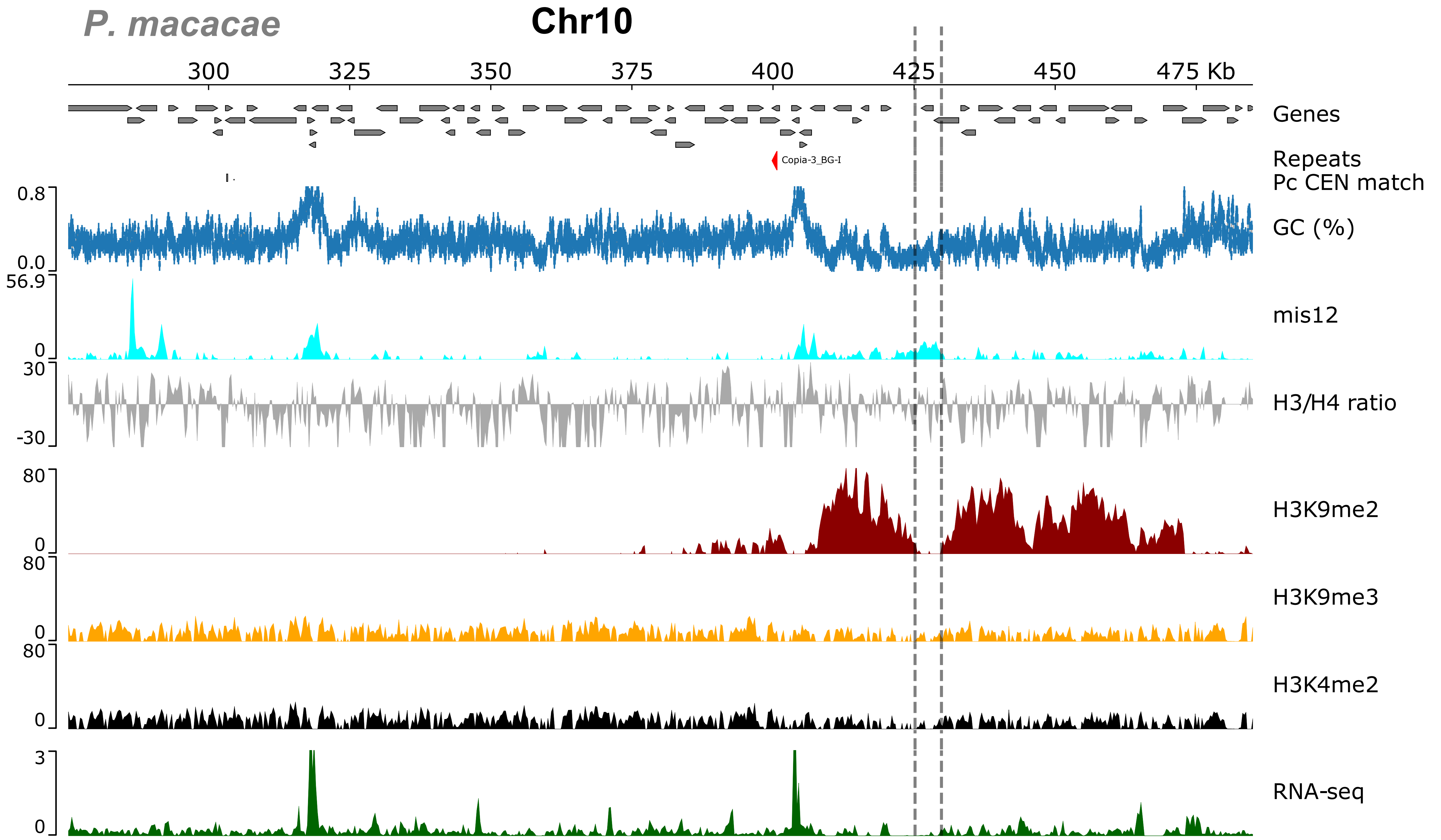
P. macacae
Chr10
300
325
350
375
400
425
450
475 Kb
Genes
Repeats
Pc CEN match
Copia-3_BG-I
GC (%)
0.8
0.0
mis12
56.9
0
H3/H4 ratio
30
-30
H3K9me2
80
0
H3K9me3
80
0
H3K4me2
80
0
RNA-seq
3
0

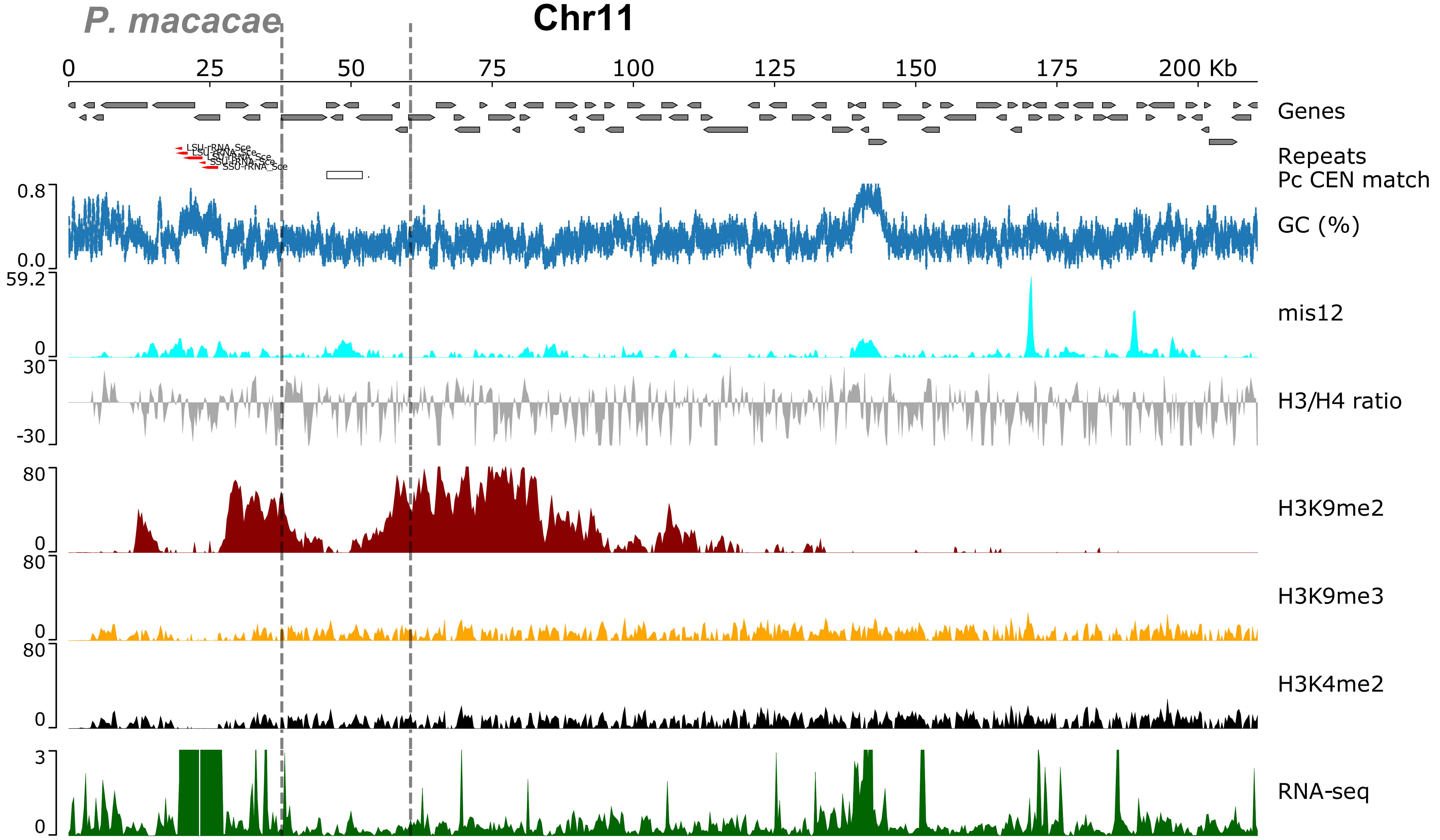
P. macacae
Chr11
0
25
50
75
100
125
150
175
200 Kb
Genes
Repeats
Pc CEN match
GC (%)
0.8
0.0
mis12
59.2
0
H3/H4 ratio
30
-30
H3K9me2
80
0
H3K9me3
80
0
H3K4me2
80
0
RNA-seq
3
0

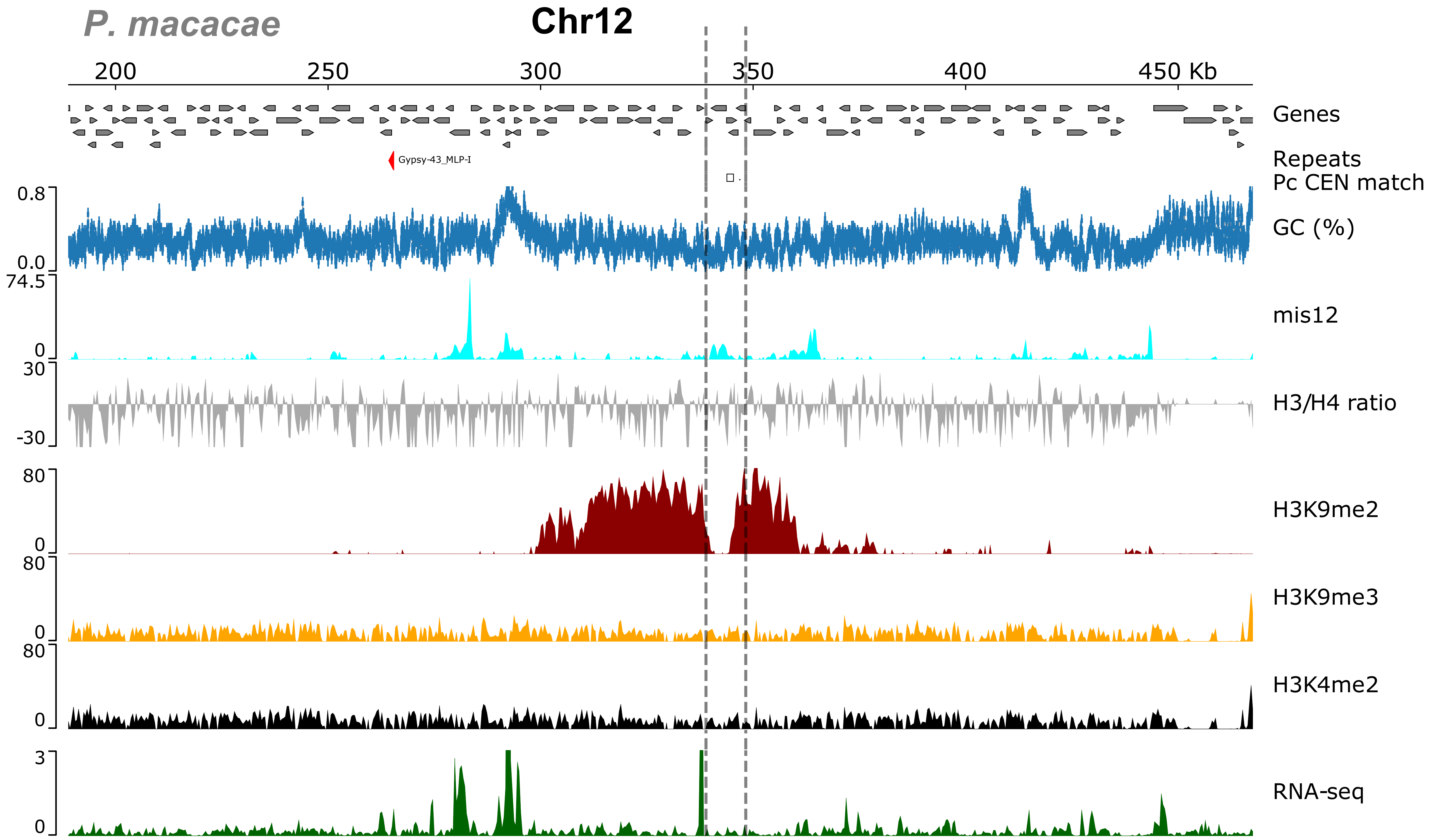

P. macacae
Chr12
200
250
300
350
400
450 Kb
Genes
Repeats
Pc CEN match
Gypsy-43_MLP-I
GC (%)
0.8
0.0
mis12
74.5
0
H3/H4 ratio
30
-30
H3K9me2
80
0
H3K9me3
80
0
H3K4me2
80
0
RNA-seq
3
0

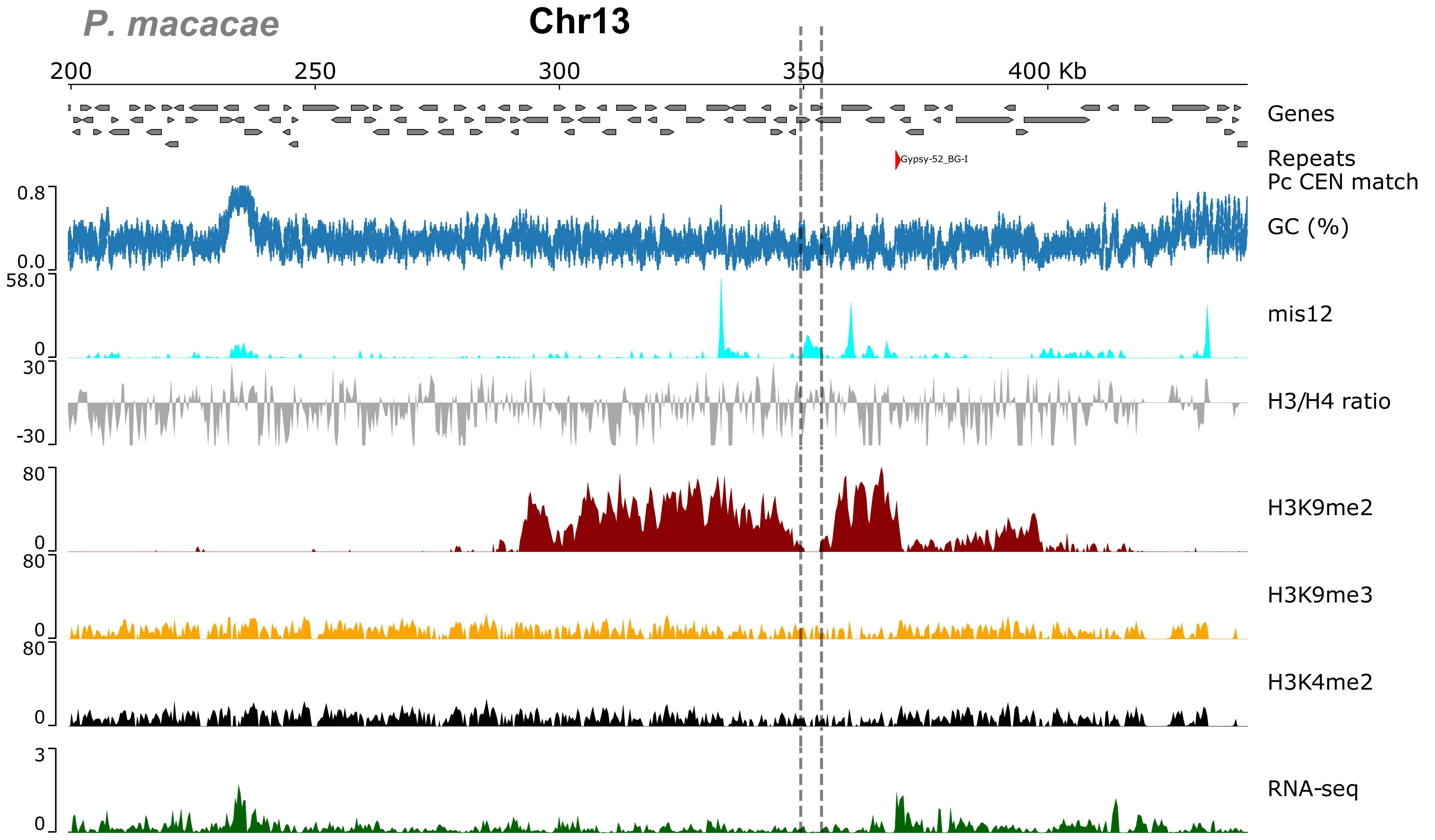

P. macacae
Chr13
200
250
300
350
400 Kb
Genes
Gypsy-52_BG-I
Repeats
Pc CEN match
0.8
0.0
GC (%)
58.0
0
mis12
30
-30
H3/H4 ratio
80
0
H3K9me2
80
0
H3K9me3
80
0
H3K4me2
3
0
RNA-seq

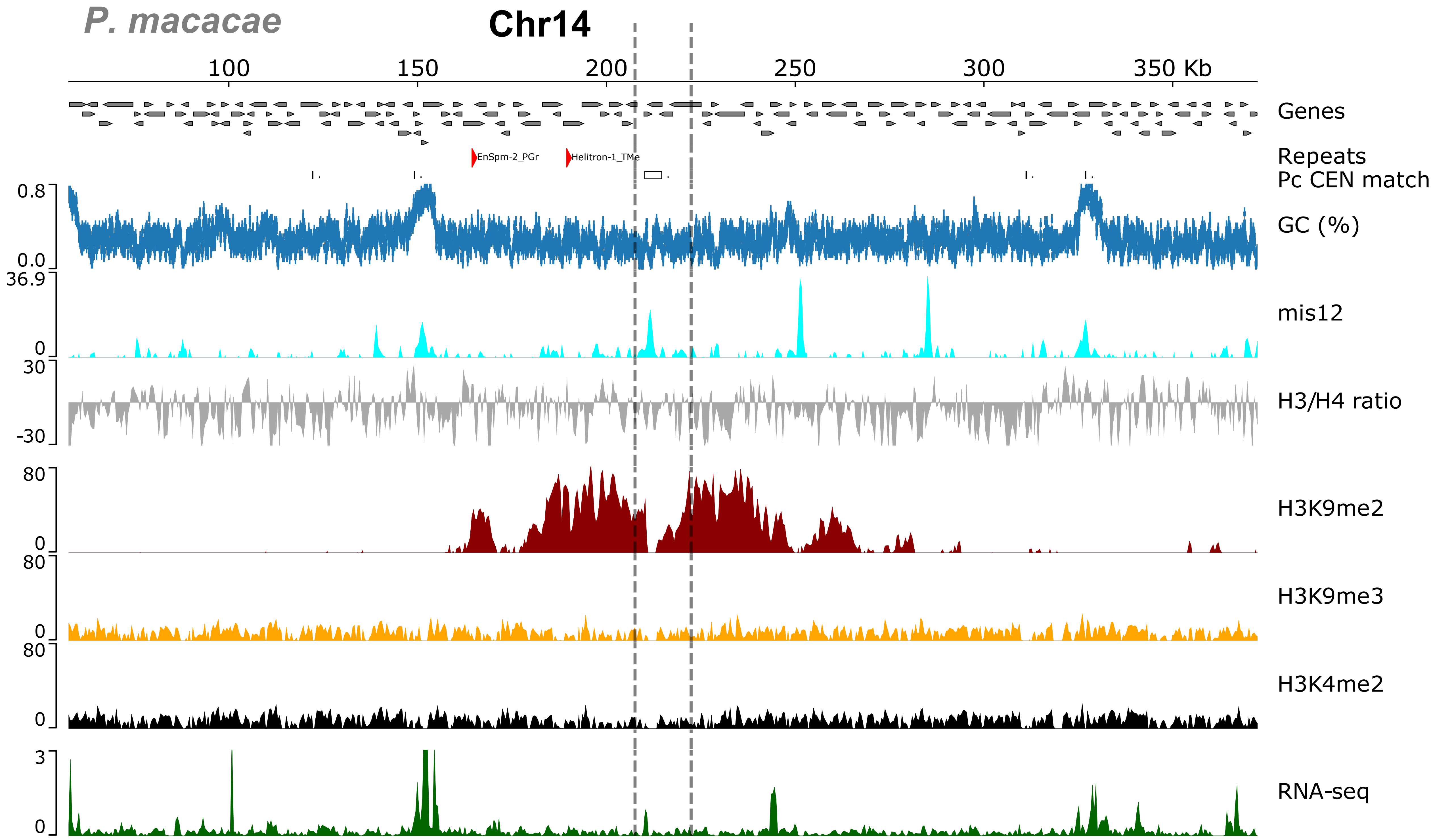
P. macacae
Chr14
100
150
200
250
300
350 Kb
Genes
EnSpm-2_PGr
Helitron-1_TMe
Repeats
Pc CEN match
0.8
0.0
GC (%)
36.9
0
mis12
30
-30
H3/H4 ratio
80
0
H3K9me2
80
0
H3K9me3
80
0
H3K4me2
3
0
RNA-seq

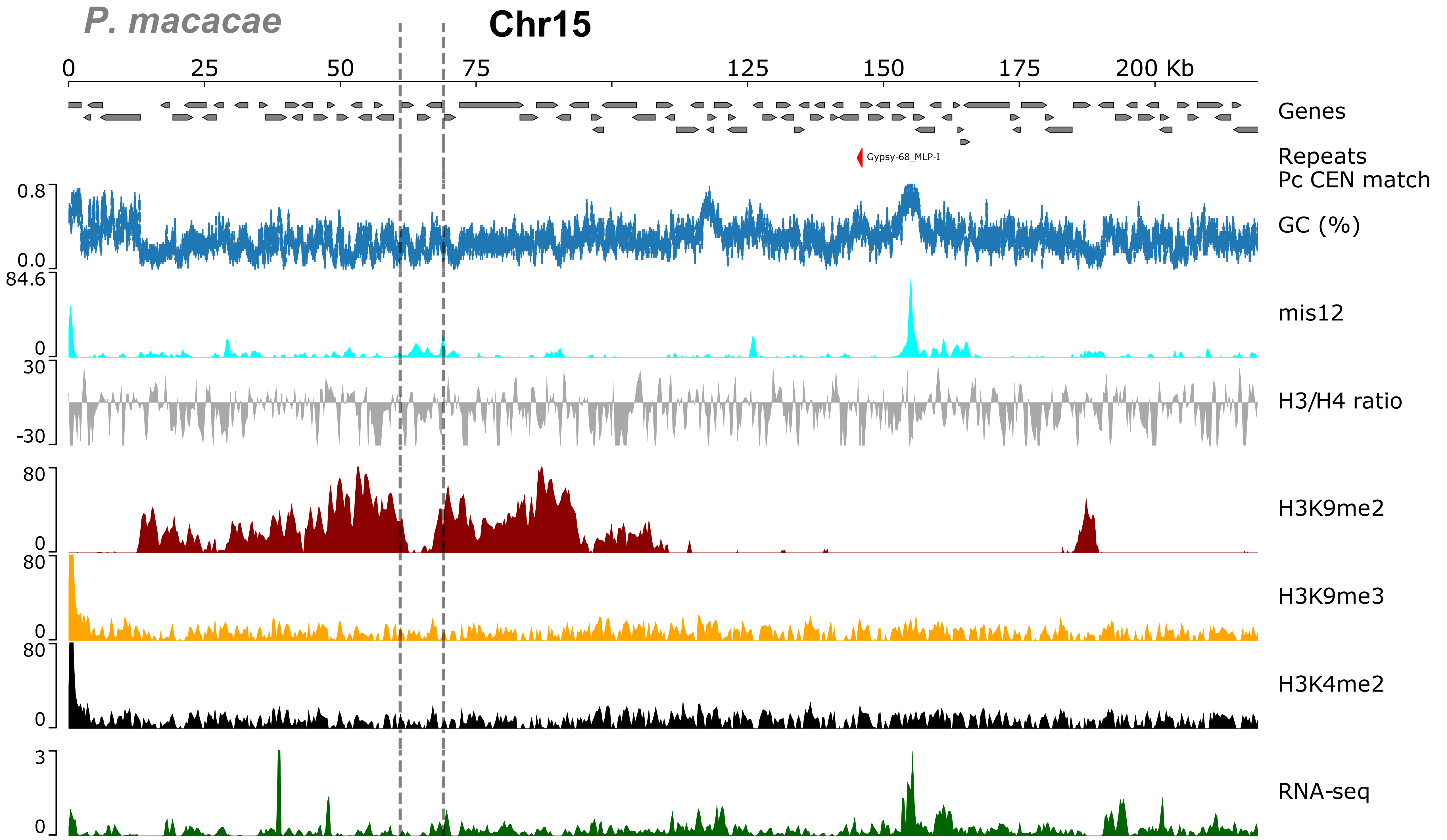

P. macacae
Chr15
0
25
50
75
125
150
175
200 Kb
Genes
Gypsy-68_MLP-I
Repeats
Pc CEN match
0.8
0.0
GC (%)
84.6
0
mis12
30
-30
H3/H4 ratio
80
0
H3K9me2
80
0
H3K9me3
80
0
H3K4me2
3
0
RNA-seq

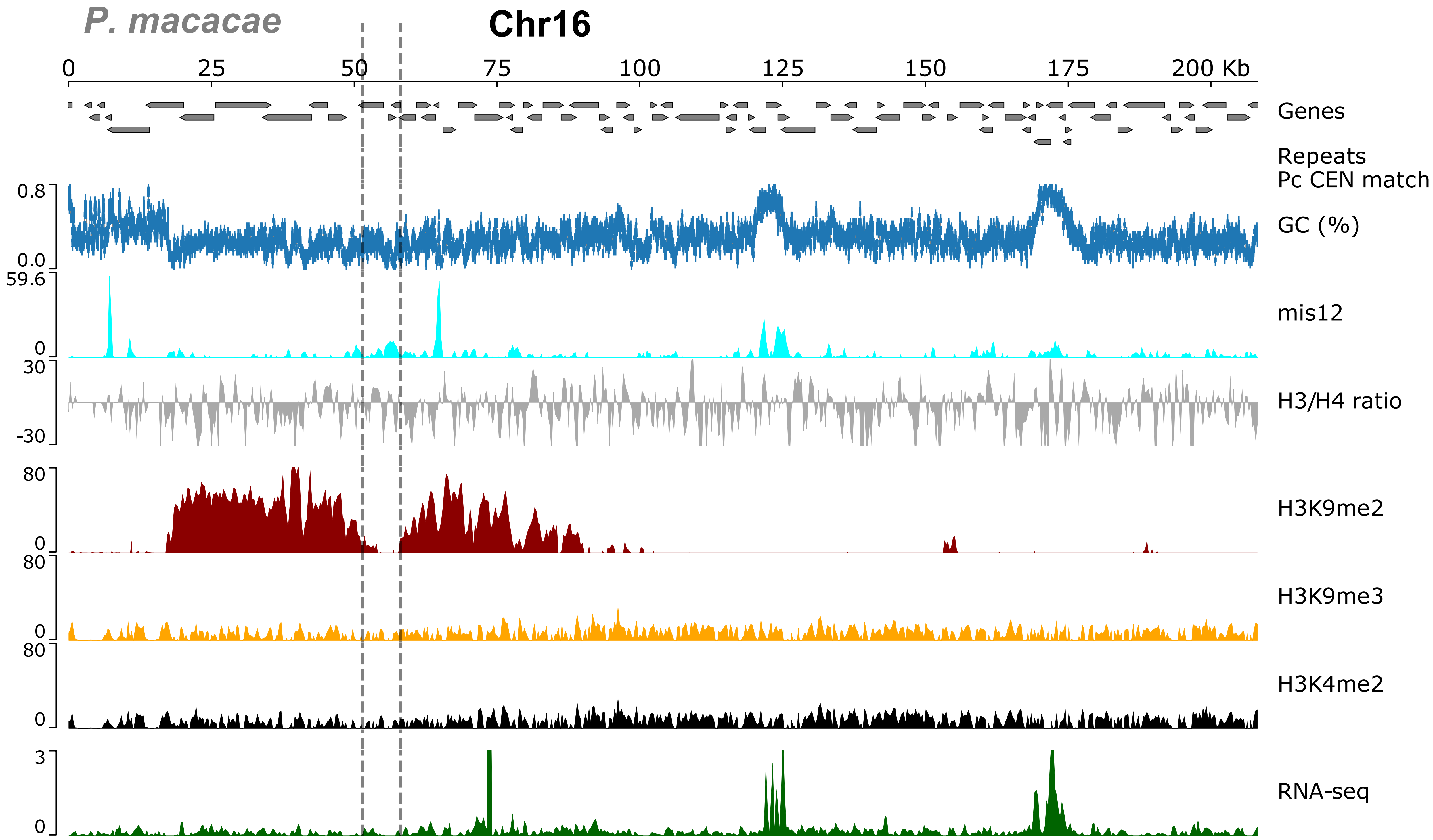
P. macacae
Chr16
0
25
50
75
100
125
150
175
200 Kb
Genes
Repeats
Pc CEN match
0.8
0.0
GC (%)
59.6
0
mis12
30
-30
H3/H4 ratio
80
0
H3K9me2
80
0
H3K9me3
80
0
H3K4me2
3
0
RNA-seq

Supplementary figure 6. Genomic view of *P. carinii* genome showing pericentromeric heterochromatin.

Genomic views of chromosomal -level scaffolds of *Pneumocystis carinii* genome subsequently showing annotated genes (directed grey boxes), repeats (DNA transposons and retrotransposons), percent GC content (blue), ChIP-seq read coverage distribution (BPM normalized over bins of 50 bp; input subtracted) of CENP-A, CENP-C, histones H3 and H4 ratio, heterochromatin-associated modifications (H3K9me2 and H3K9me3), euchromatin (H3K4me2) and gene expression (RNA-seq) in relation with centromeres.

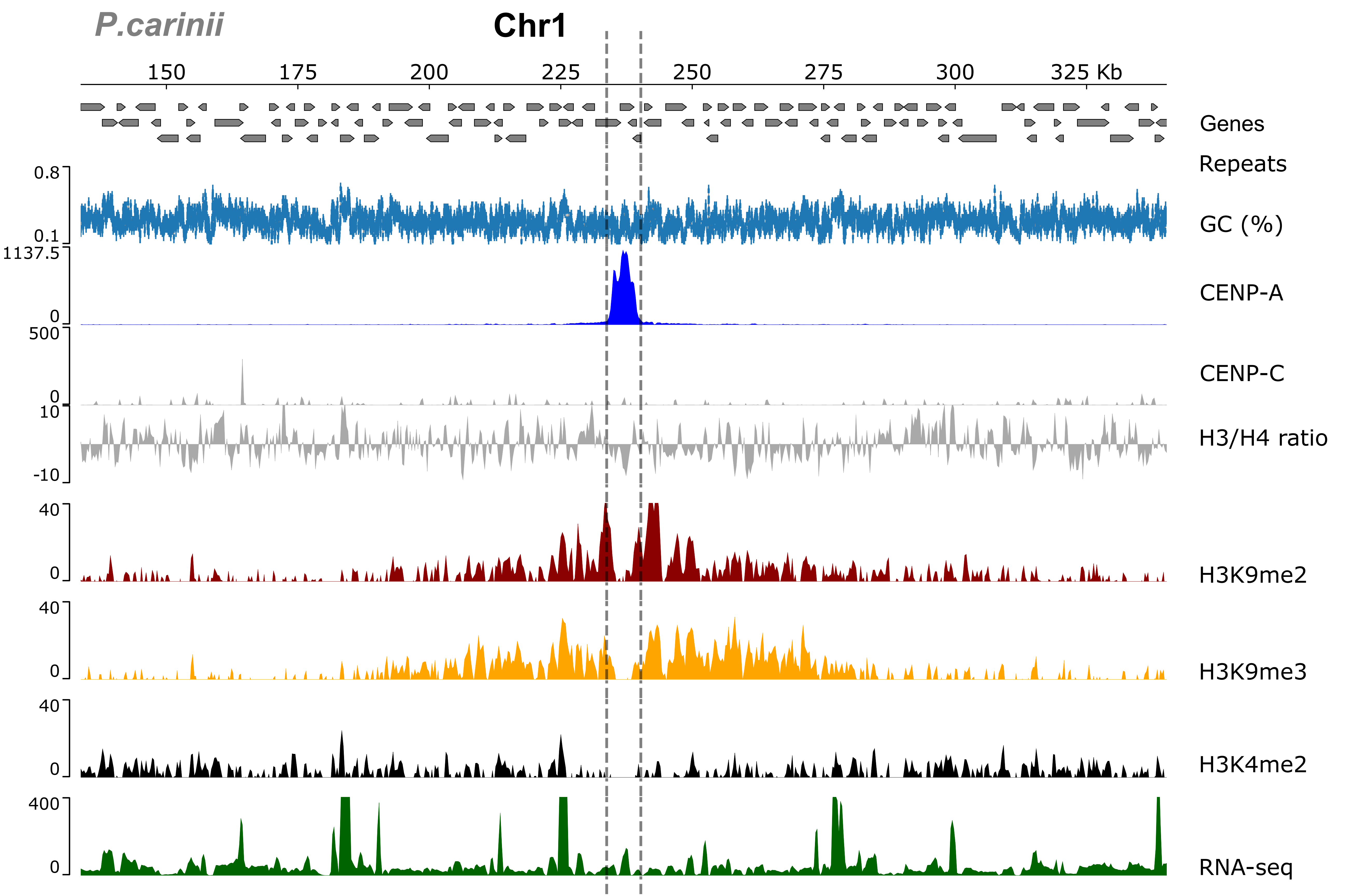

P.carinii
Chr1
150
175
200
225
250
275
300
325 Kb
Genes
Repeats
0.8
0.1
GC (%)
1137.5
0
CENP-A
500
0
CENP-C
10
-10
H3/H4 ratio
40
0
H3K9me2
40
0
H3K9me3
40
0
H3K4me2
400
0
RNA-seq

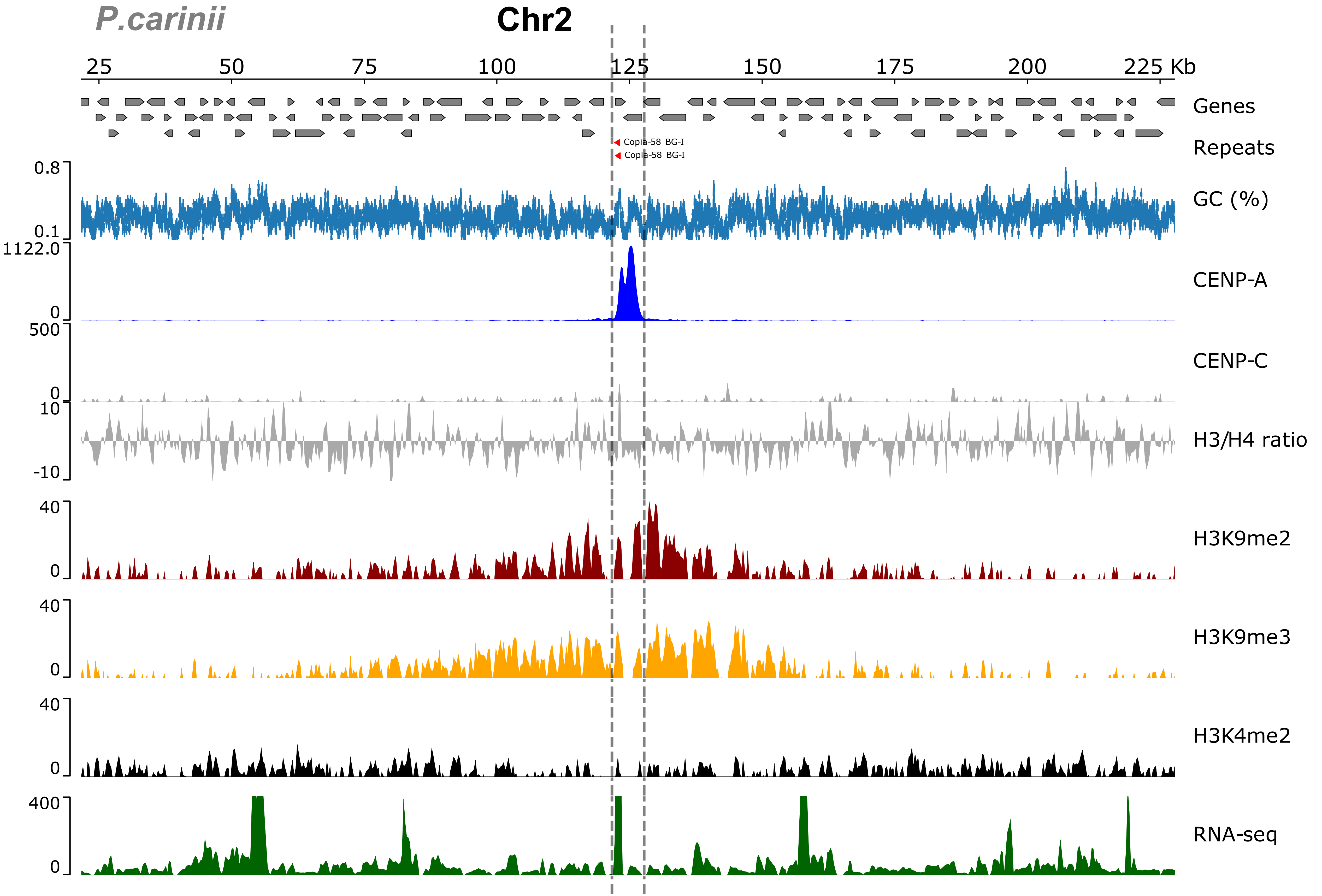

P.carinii
Chr2
25
50
75
100
125
150
175
200
225 Kb
Genes
Repeats
Copia-58_BG-I
Copia-58_BG-I
0.8
0.1
GC (%)
1122.0
0
CENP-A
500
0
CENP-C
10
-10
H3/H4 ratio
40
0
H3K9me2
40
0
H3K9me3
40
0
H3K4me2
400
0
RNA-seq

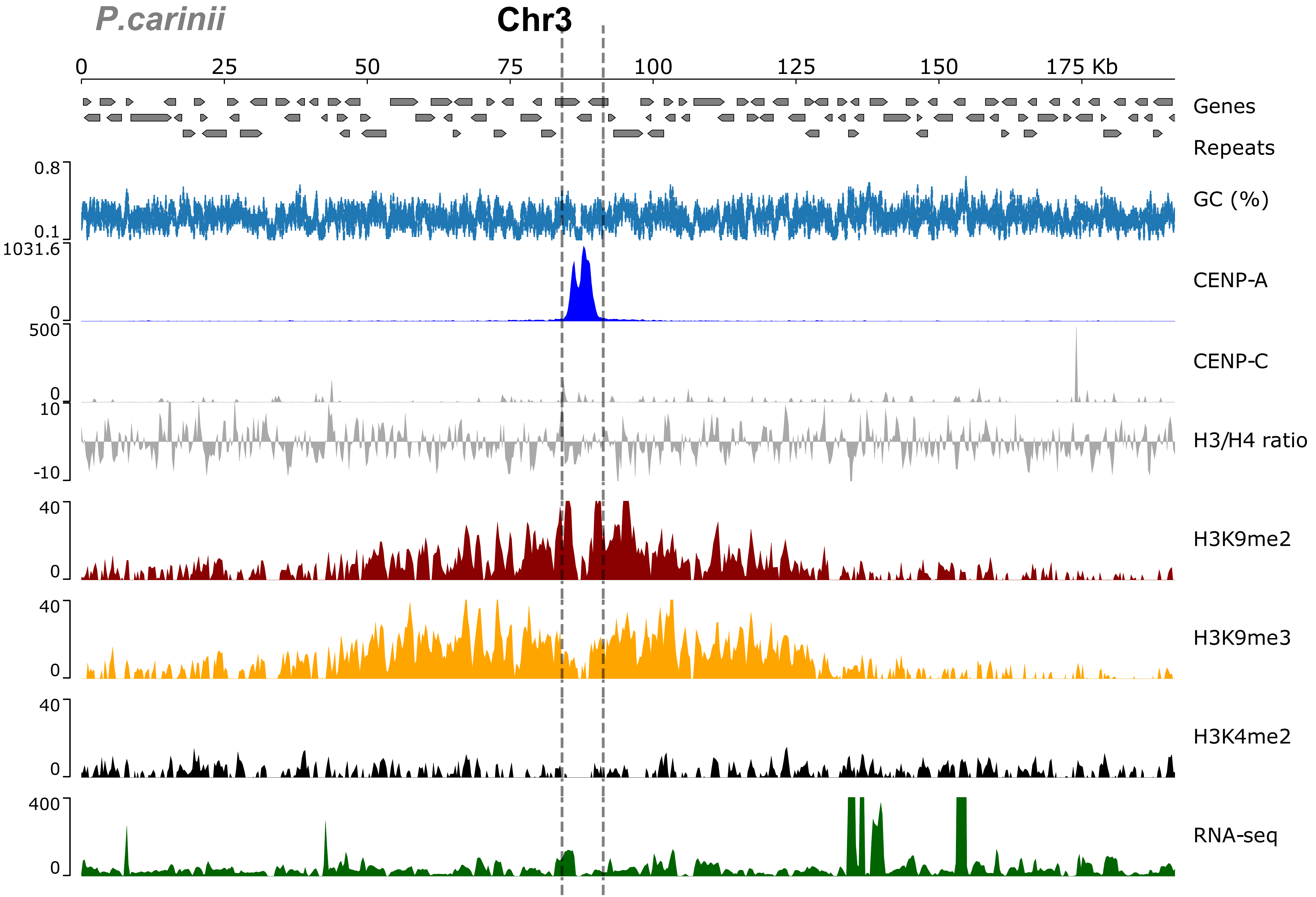

P.carinii
Chr3
0
25
50
75
100
125
150
175 Kb
Genes
Repeats
0.8
0.1
GC (%)
1031.6
0
CENP-A
500
0
CENP-C
10
-10
H3/H4 ratio
40
0
H3K9me2
40
0
H3K9me3
40
0
H3K4me2
400
0
RNA-seq

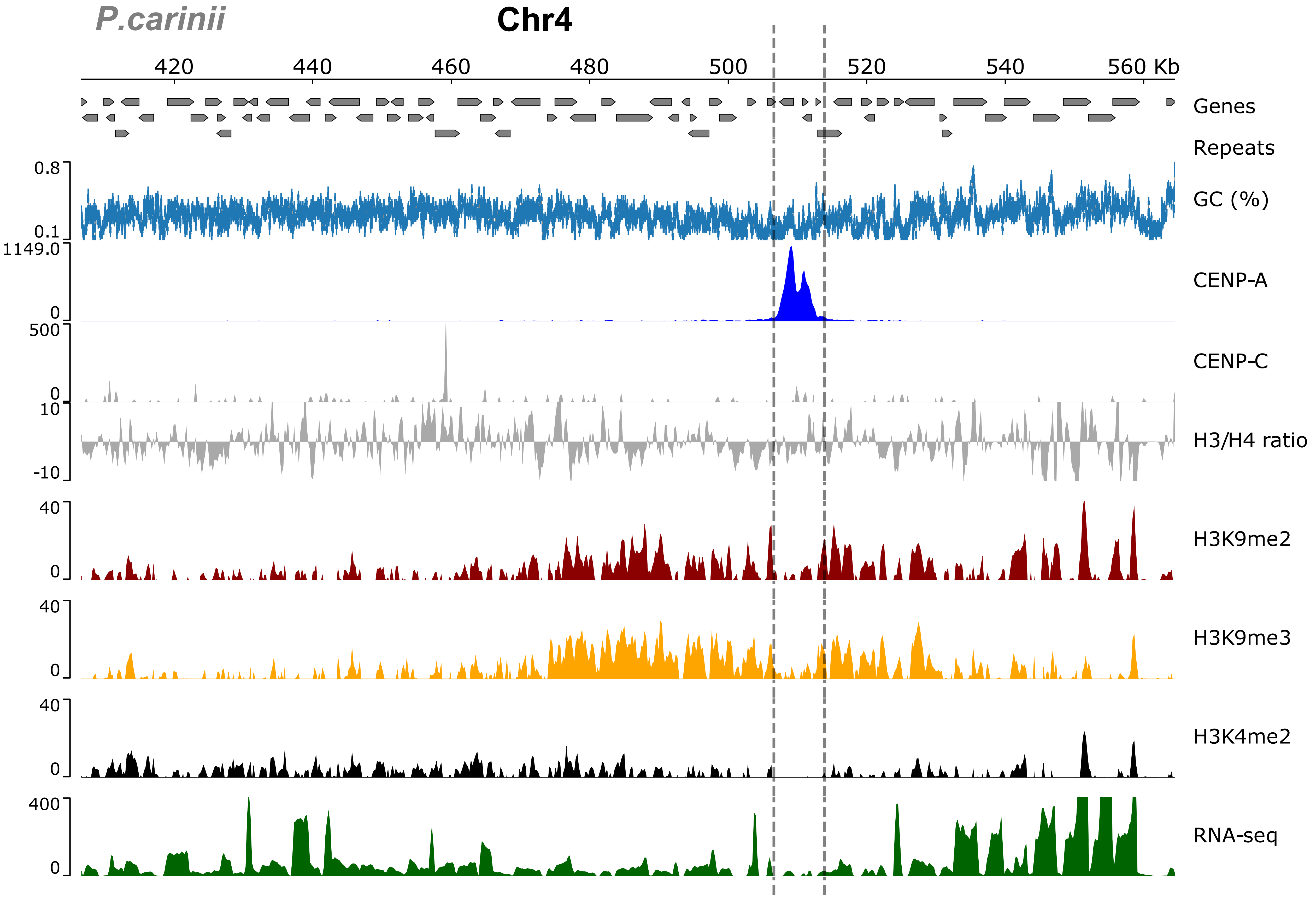

P.carinii
Chr4
420
440
460
480
500
520
540
560 Kb
Genes
Repeats
0.8
0.1
GC (%)
1149.0
0
CENP-A
500
0
CENP-C
10
-10
H3/H4 ratio
40
0
H3K9me2
40
0
H3K9me3
40
0
H3K4me2
400
0
RNA-seq

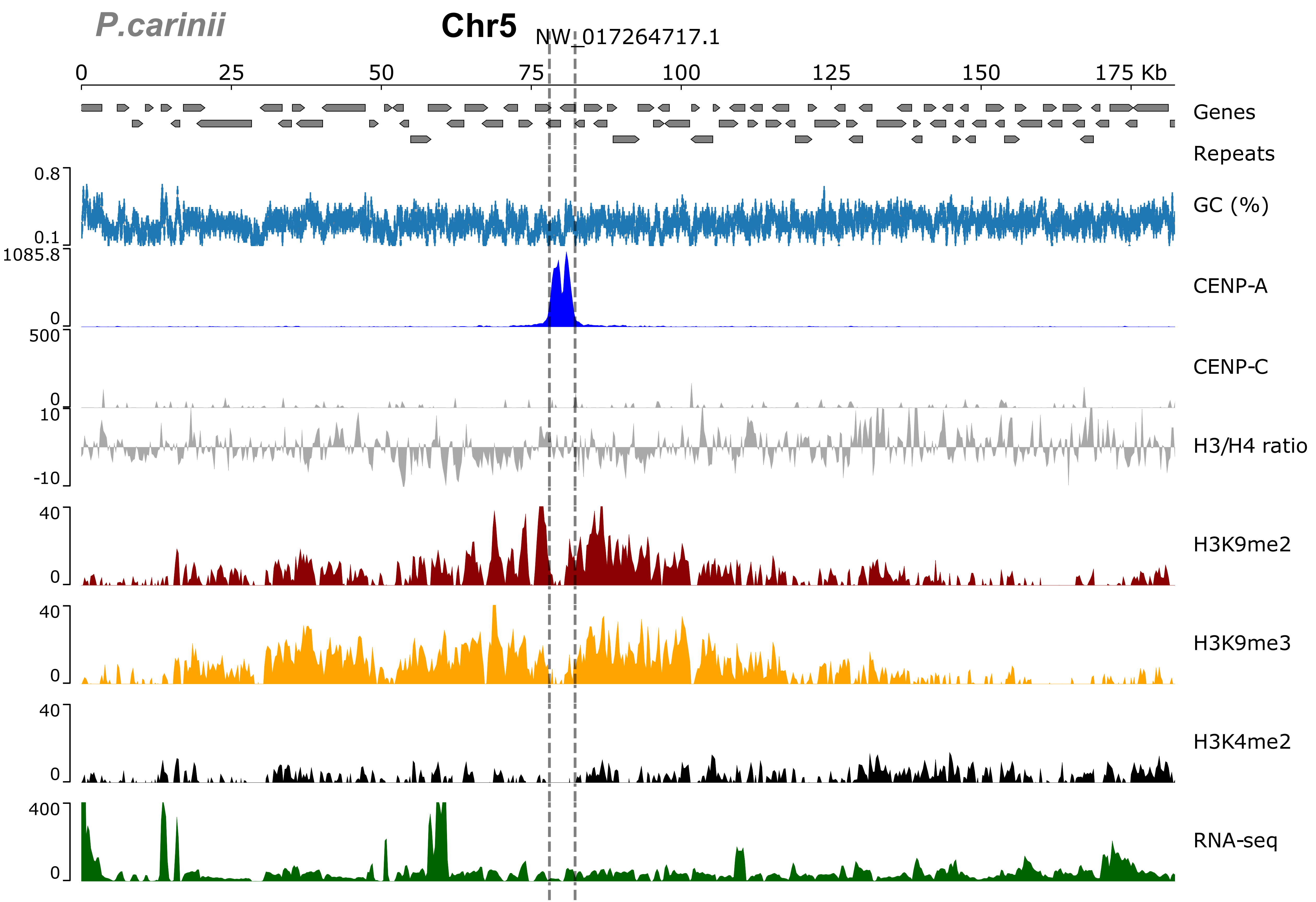

P.carinii
Chr5
NW_017264717.1
0
25
50
75
100
125
150
175 Kb
Genes
Repeats
0.8
0.1
GC (%)
1085.8
0
CENP-A
500
0
CENP-C
10
-10
H3/H4 ratio
40
0
H3K9me2
40
0
H3K9me3
40
0
H3K4me2
400
0
RNA-seq

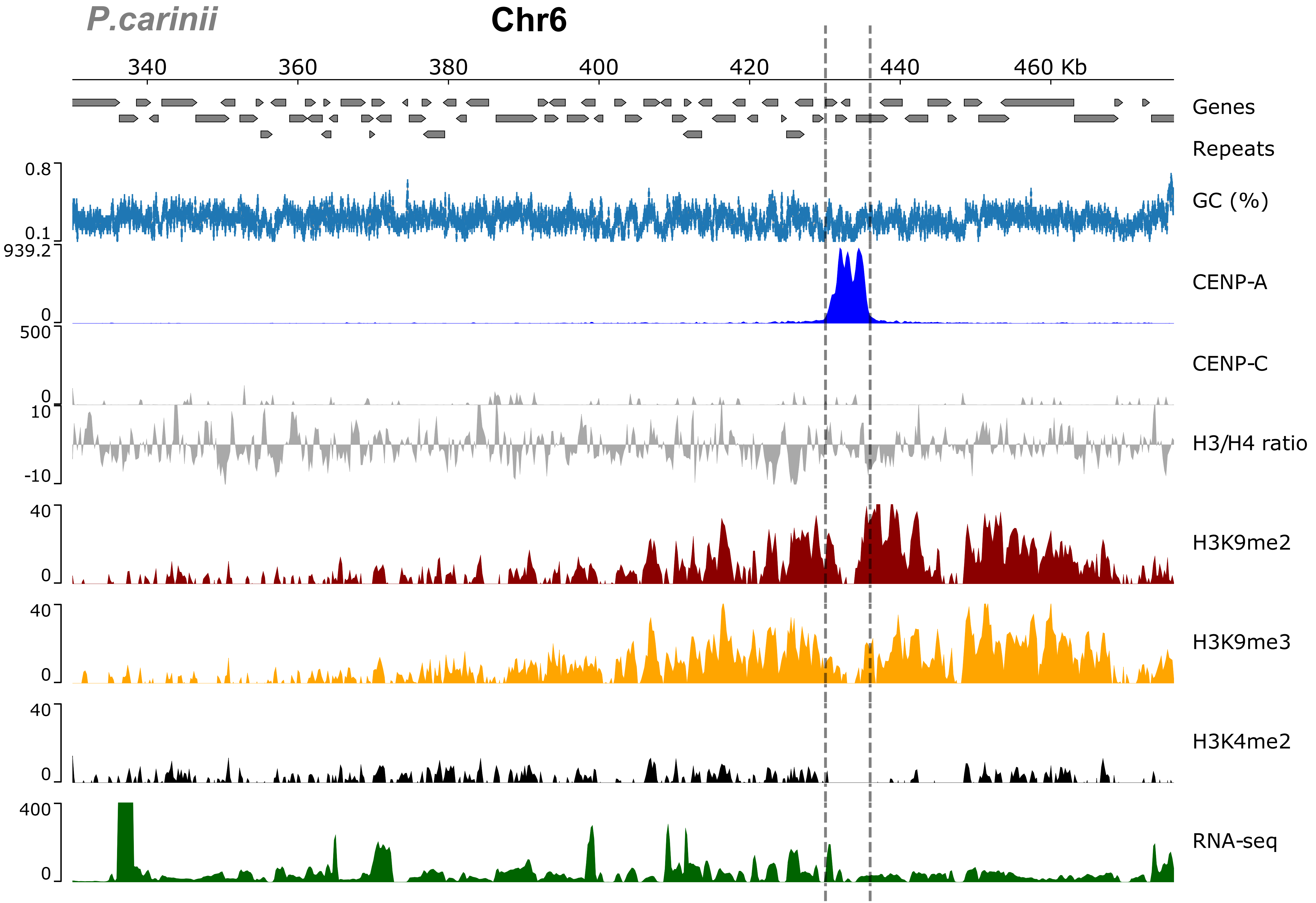

P.carinii
Chr6
340
360
380
400
420
440
460 Kb
Genes
Repeats
0.8
0.1
GC (%)
939.2
0
CENP-A
500
0
CENP-C
10
-10
H3/H4 ratio
40
0
H3K9me2
40
0
H3K9me3
40
0
H3K4me2
400
0
RNA-seq

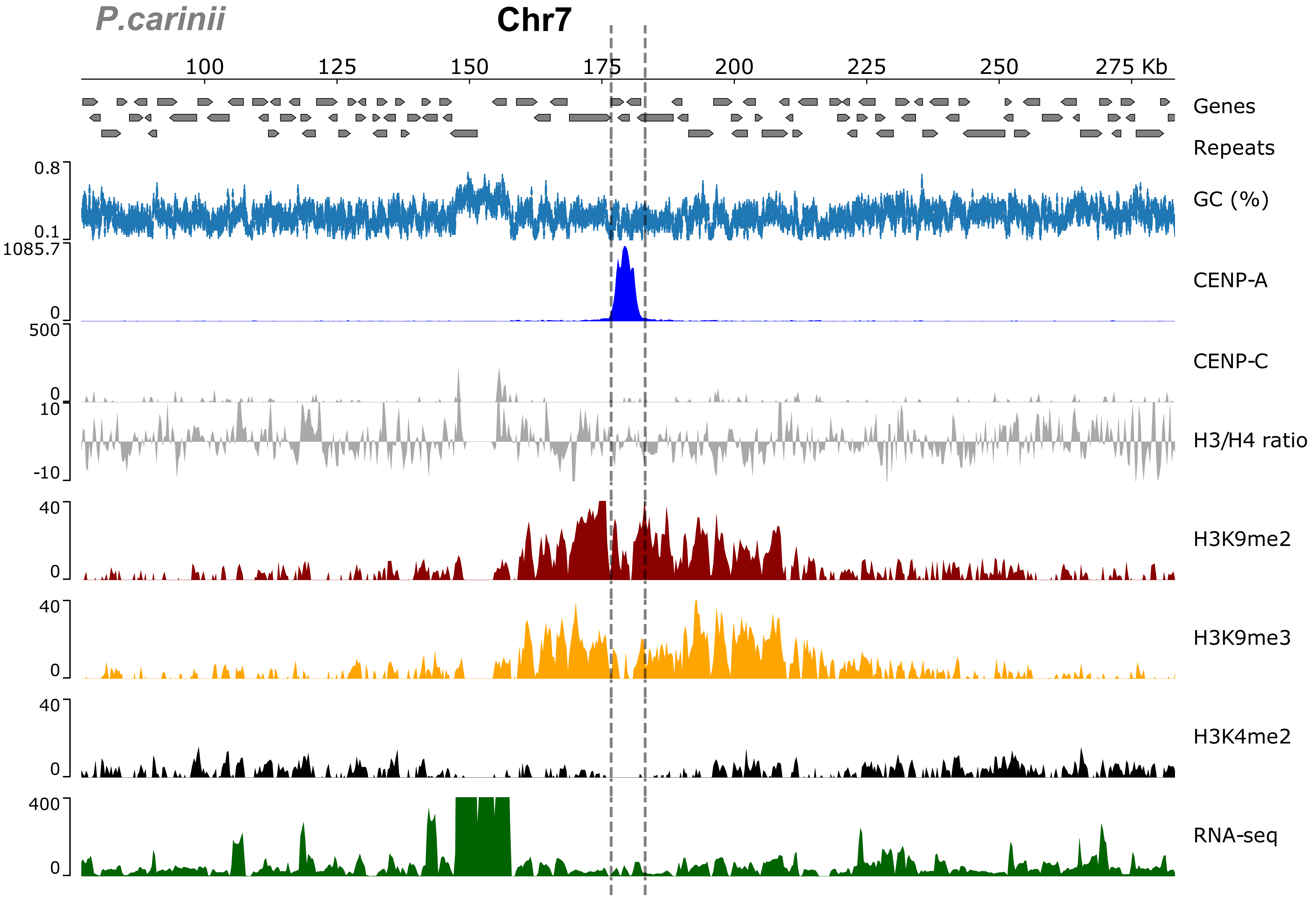

P.carinii
Chr7
100
125
150
175
200
225
250
275 Kb
Genes
Repeats
0.8
0.1
GC (%)
1085.7
0
CENP-A
500
0
CENP-C
10
-10
H3/H4 ratio
40
0
H3K9me2
40
0
H3K9me3
40
0
H3K4me2
400
0
RNA-seq

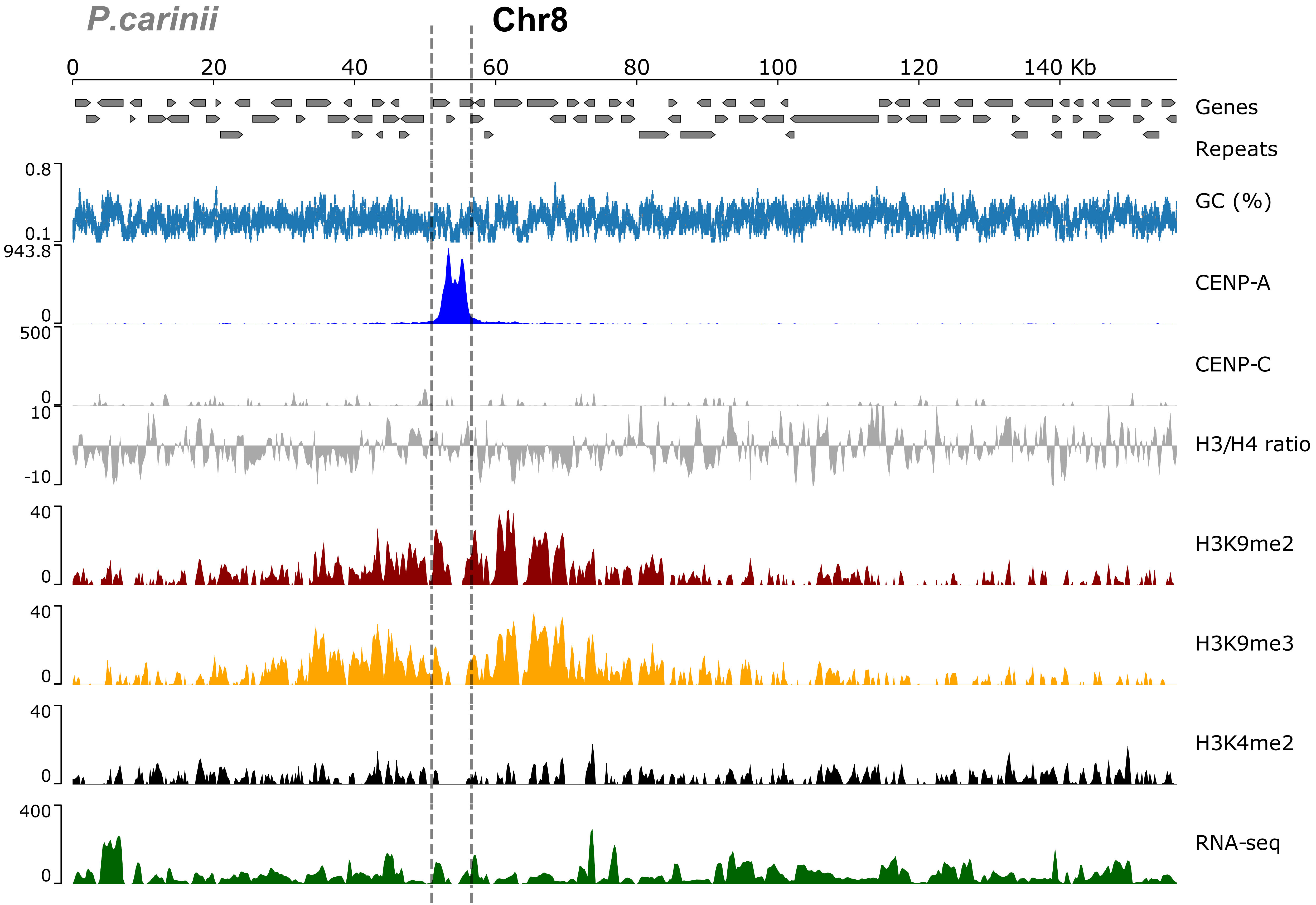

P.carinii
Chr8
0
20
40
60
80
100
120
140 Kb
Genes
Repeats
0.8
0.1
GC (%)
943.8
0
CENP-A
500
0
CENP-C
10
-10
H3/H4 ratio
40
0
H3K9me2
40
0
H3K9me3
40
0
H3K4me2
400
0
RNA-seq

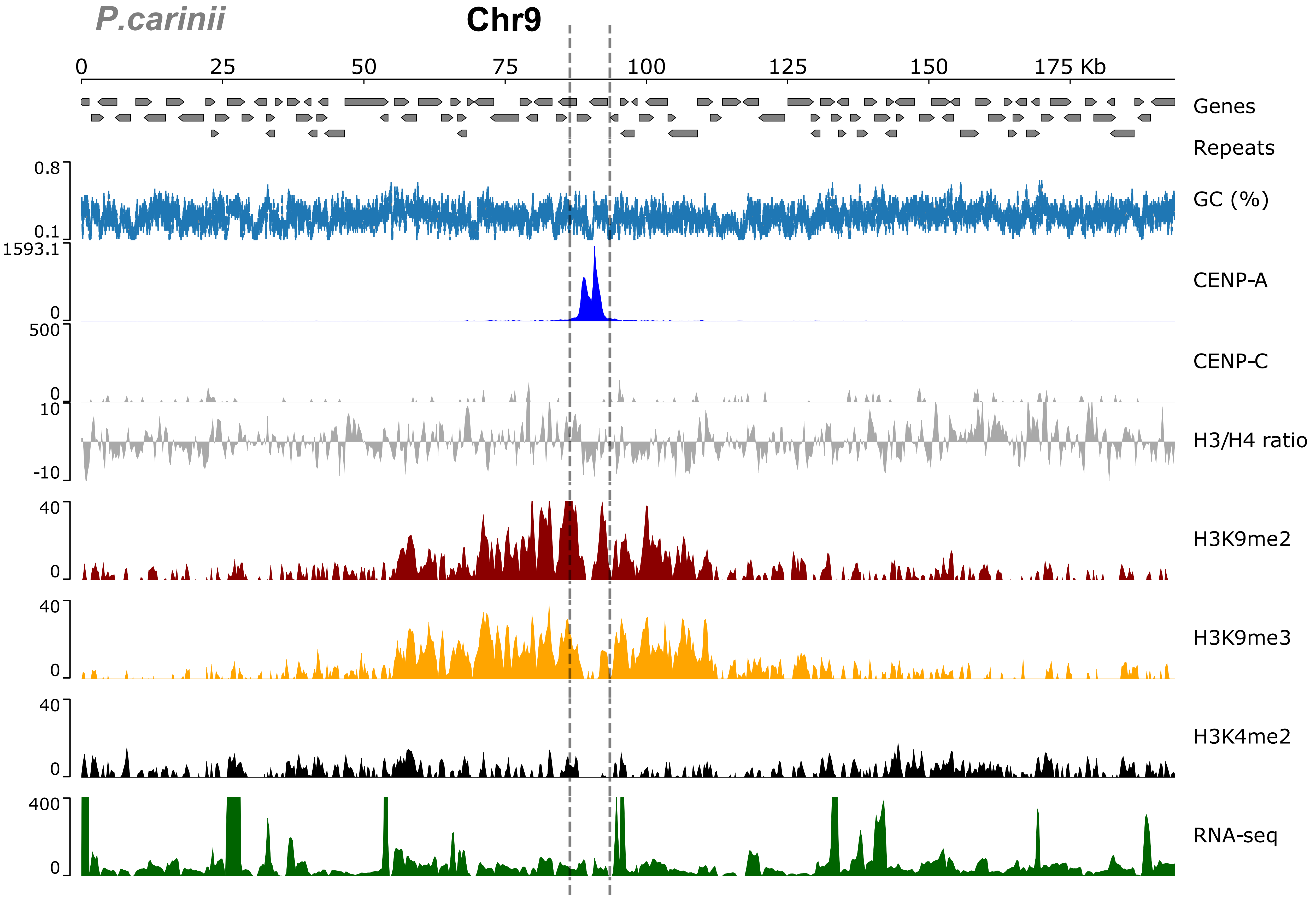

P.carinii
Chr9
0
25
50
75
100
125
150
175 Kb
Genes
Repeats
0.8
0.1
GC (%)
1593.1
0
CENP-A
500
0
CENP-C
10
-10
H3/H4 ratio
40
0
H3K9me2
40
0
H3K9me3
40
0
H3K4me2
400
0
RNA-seq

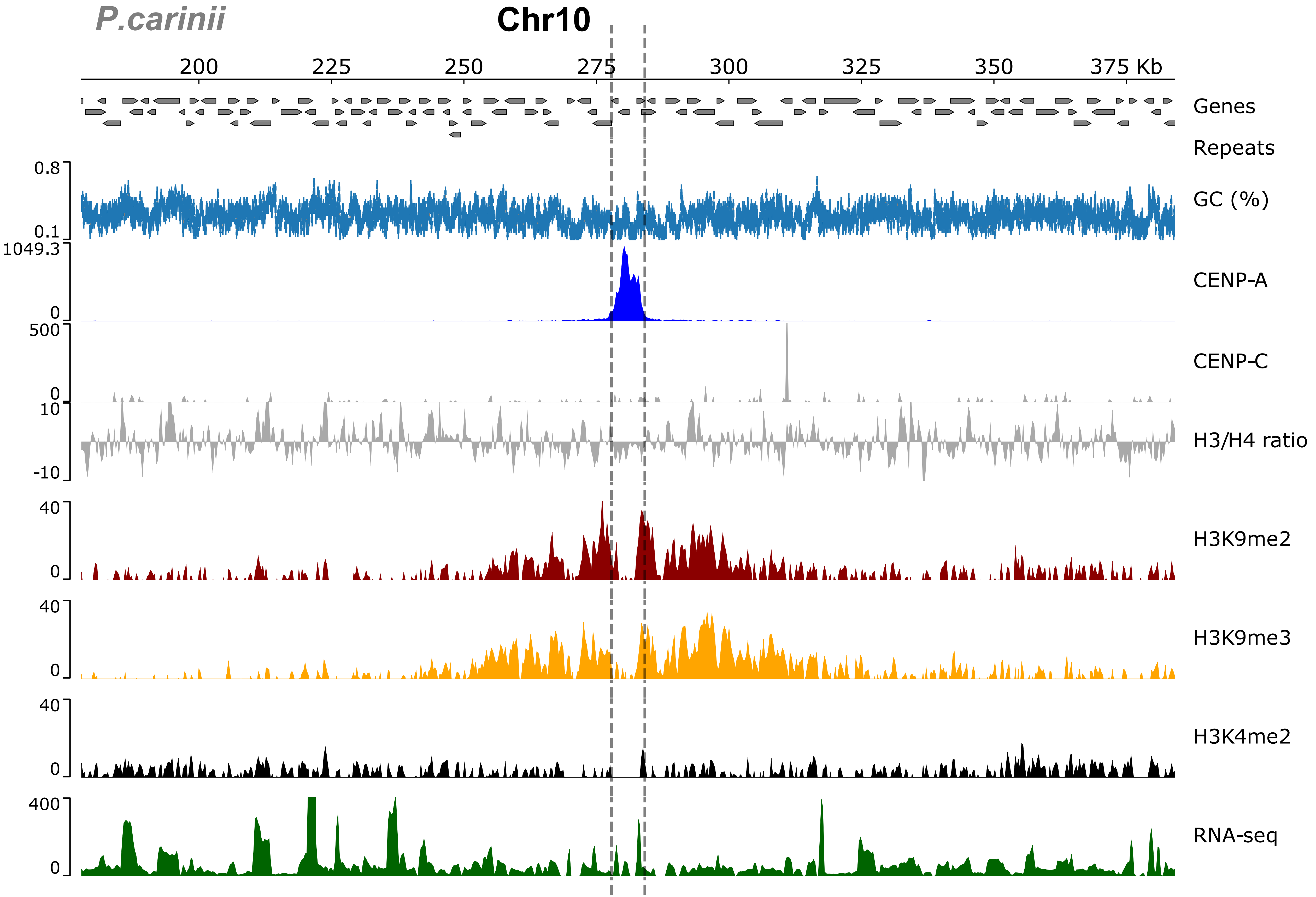

P.carinii
Chr10
200
225
250
275
300
325
350
375 Kb
Genes
Repeats
0.8
0.1
GC (%)
1049.3
0
CENP-A
500
0
CENP-C
10
-10
H3/H4 ratio
40
0
H3K9me2
40
0
H3K9me3
40
0
H3K4me2
400
0
RNA-seq

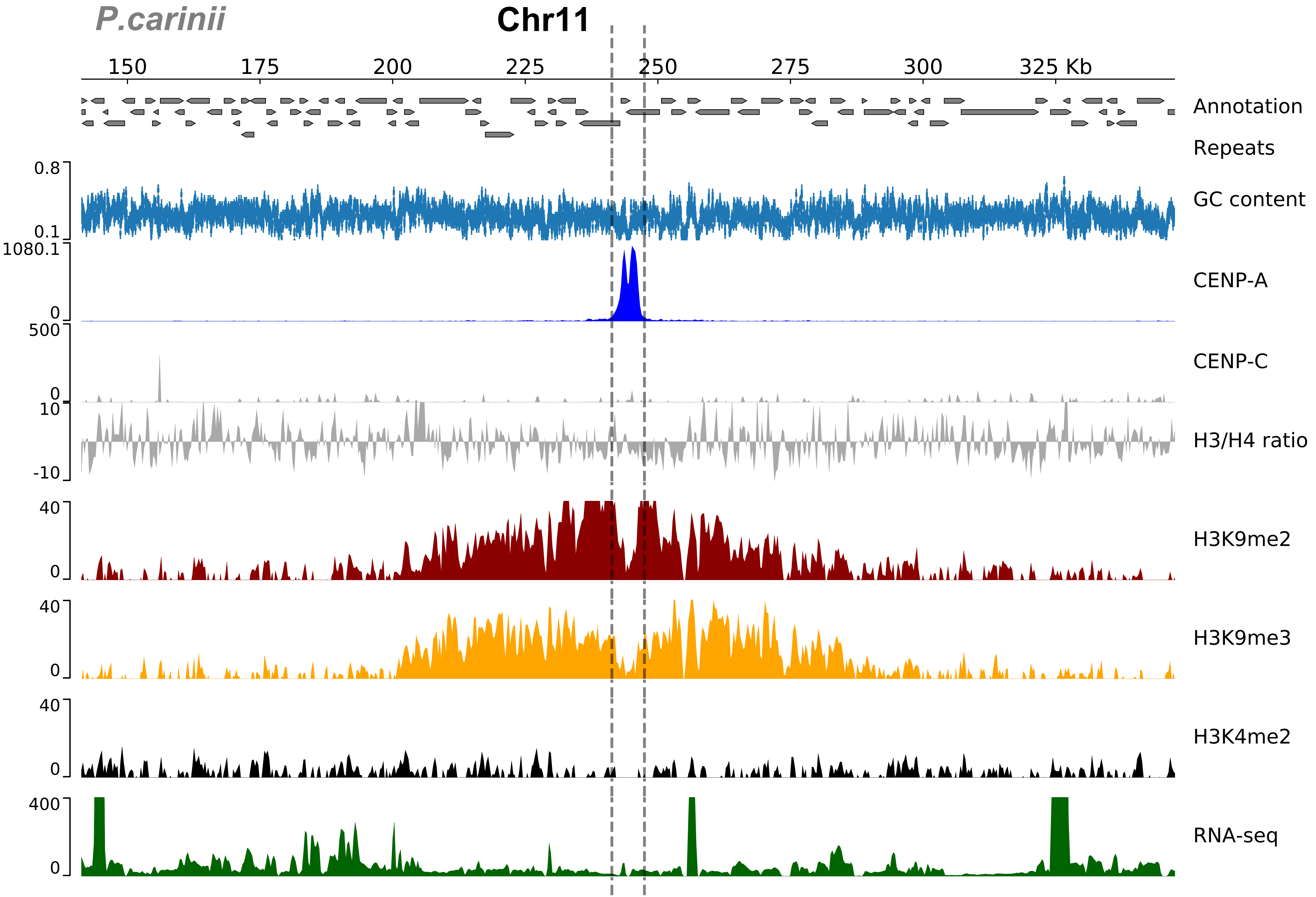

P.carinii
Chr11
150
175
200
225
250
275
300
325 Kb
Annotation
Repeats
0.8
0.1
GC content
1080.1
0
CENP-A
500
0
CENP-C
10
-10
H3/H4 ratio
40
0
H3K9me2
40
0
H3K9me3
40
0
H3K4me2
400
0
RNA-seq

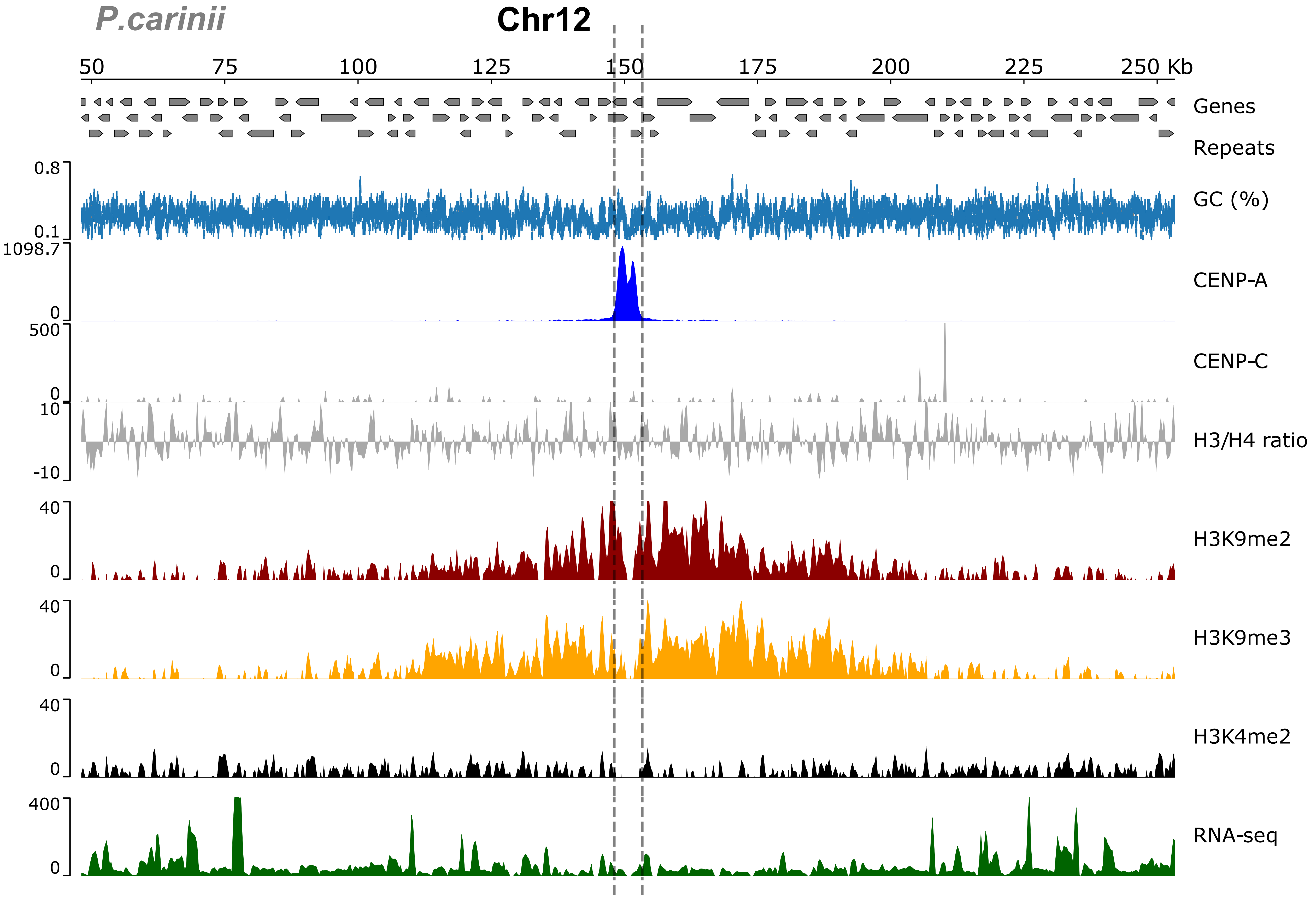

P.carinii
Chr12
50
75
100
125
150
175
200
225
250 Kb
Genes
Repeats
0.8
0.1
GC (%)
1098.7
0
CENP-A
500
0
CENP-C
10
-10
H3/H4 ratio
40
0
H3K9me2
40
0
H3K9me3
40
0
H3K4me2
400
0
RNA-seq

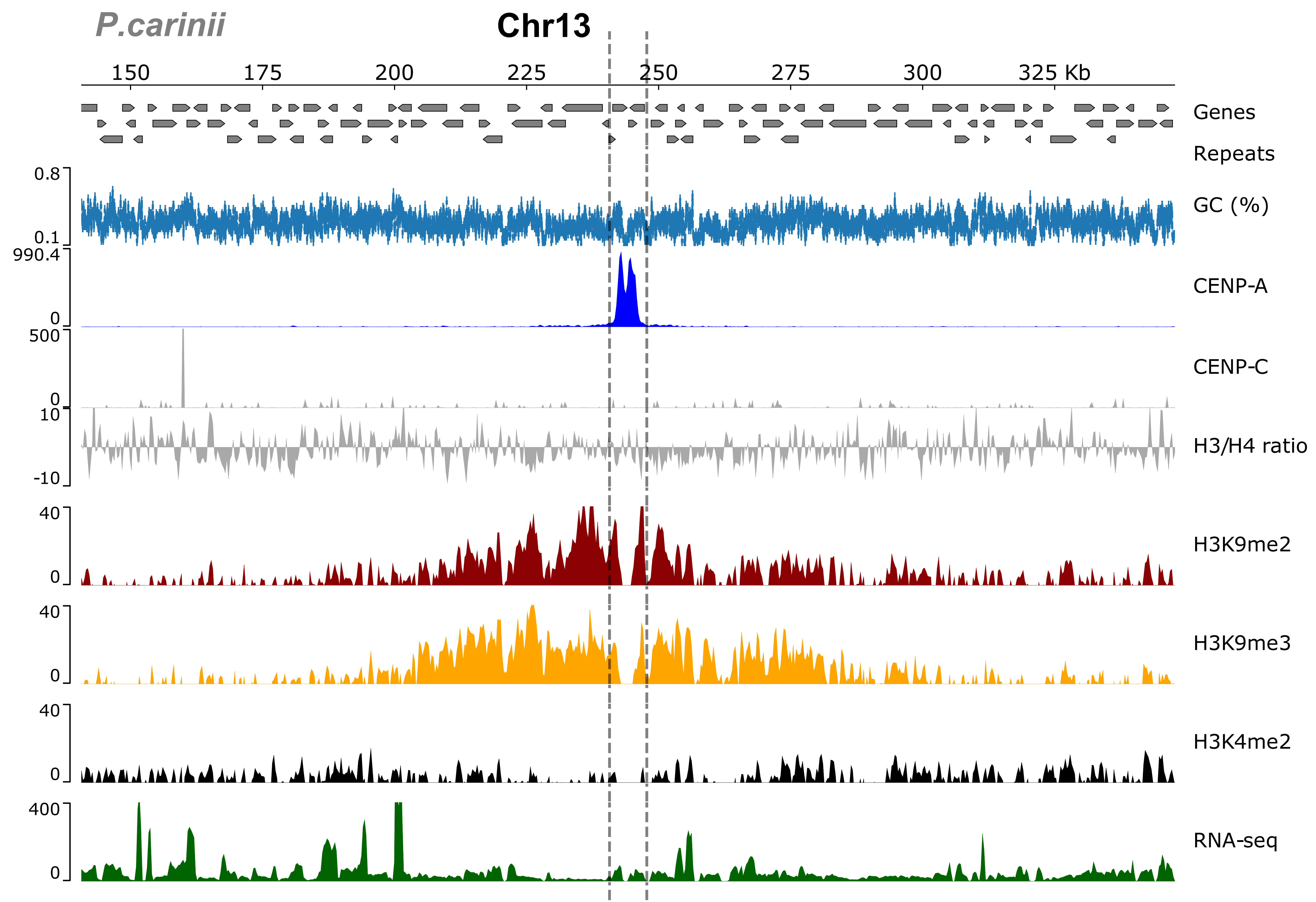
P.carinii
Chr13
150
175
200
225
250
275
300
325 Kb
Genes
Repeats
0.8
0.1
GC (%)
990.4
0
CENP-A
500
0
CENP-C
10
-10
H3/H4 ratio
40
0
H3K9me2
40
0
H3K9me3
40
0
H3K4me2
400
0
RNA-seq

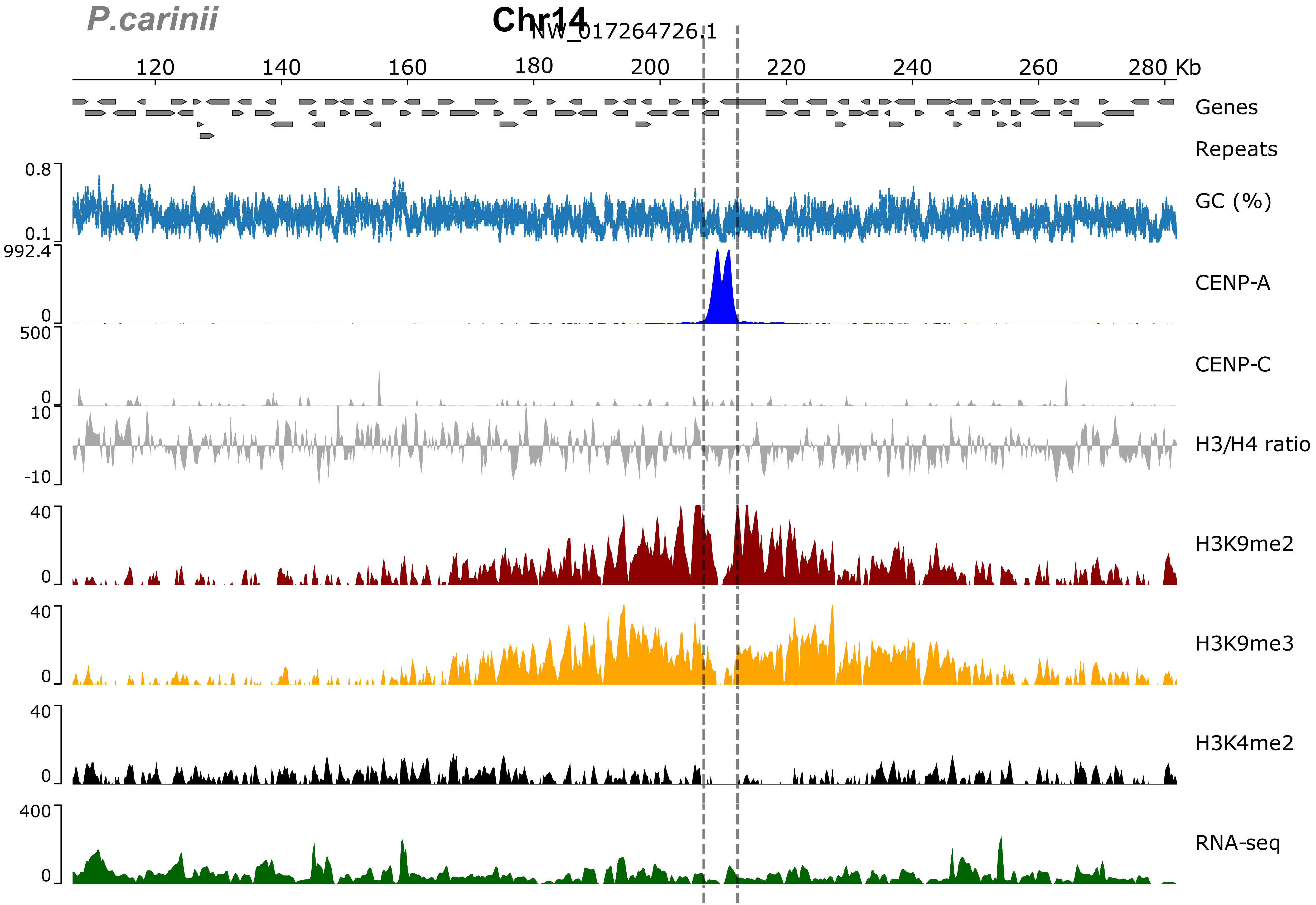

P.carinii
Chr14
NW_017264726.1
120
140
160
180
200
220
240
260
280 Kb
Genes
Repeats
0.8
0.1
GC (%)
992.4
0
CENP-A
500
0
CENP-C
10
-10
H3/H4 ratio
40
0
H3K9me2
40
0
H3K9me3
40
0
H3K4me2
400
0
RNA-seq

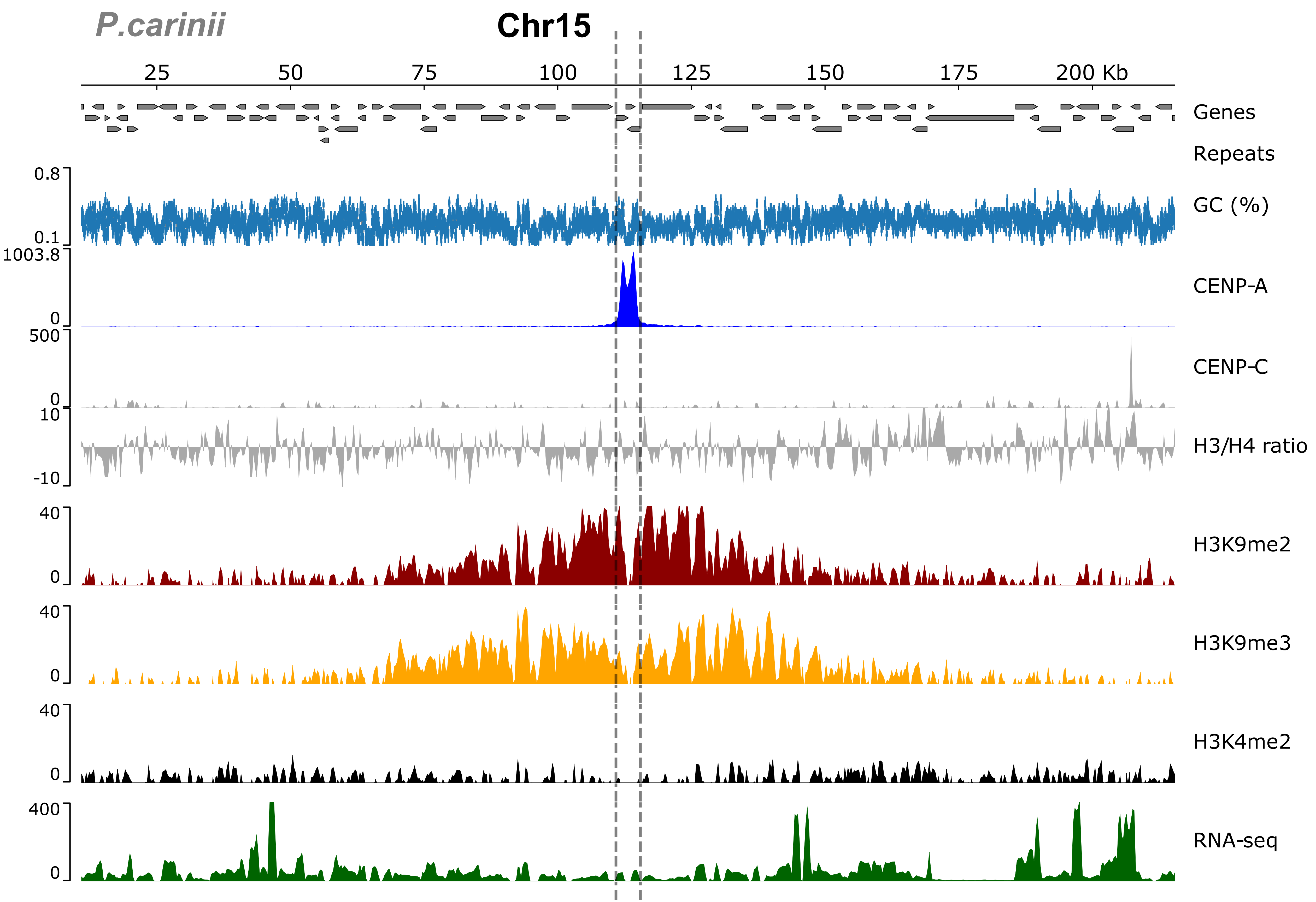

P.carinii
Chr15
25
50
75
100
125
150
175
200 Kb
Genes
Repeats
0.8
0.1
GC (%)
1003.8
0
CENP-A
500
0
CENP-C
10
-10
H3/H4 ratio
40
0
H3K9me2
40
0
H3K9me3
40
0
H3K4me2
400
0
RNA-seq

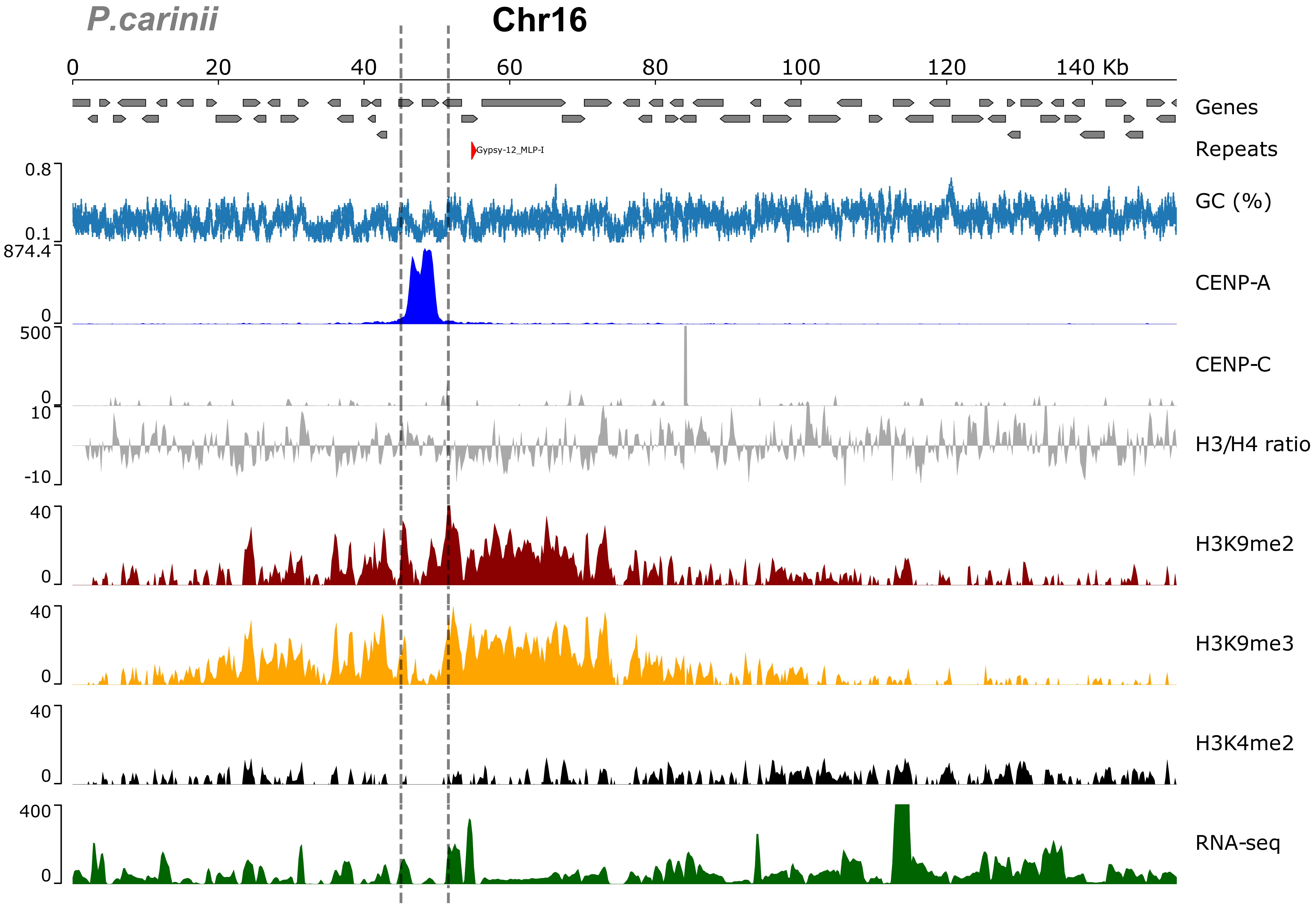

P.carinii
Chr16
0
20
40
60
80
100
120
140 Kb
Genes
Repeats
Gypsy-12_MLP-I
0.8
0.1
GC (%)
874.4
0
CENP-A
500
0
CENP-C
10
-10
H3/H4 ratio
40
0
H3K9me2
40
0
H3K9me3
40
0
H3K4me2
400
0
RNA-seq

Supplementary figure 7. Genomic view of *P. murina* genome showing pericentromeric heterochromatin.

Genomic views of *Pneumocystis murina* scaffolds subsequently showing annotated genes (directed grey boxes), repeats (DNA transposons and retrotransposons), percent GC content (blue), ChIP-seq read coverage distribution (BPM normalized over bins of 50 bp; input subtracted) of CENP-A, CENP-C, histones H3 and H4 ratio, heterochromatin-associated modifications (H3K9me2 and H3K9me3), euchromatin (H3K4me2) and gene expression (RNA-seq) in relation with centromeres.

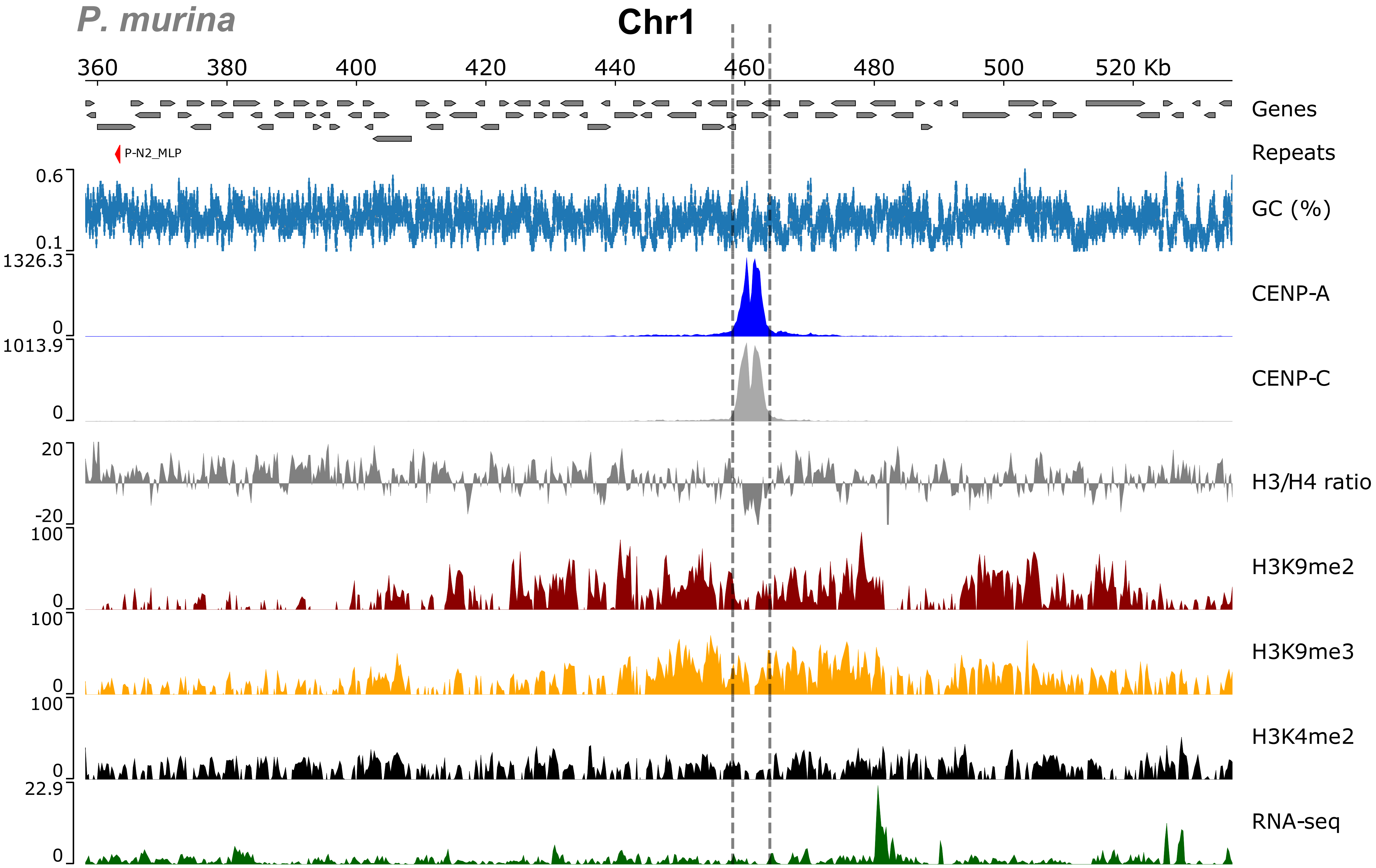
P. murina
Chr1
360
380
400
420
440
460
480
500
520 Kb
Genes
Repeats
P-N2_MLP
GC (%)
0.6
0.1
CENP-A
1326.3
0
CENP-C
1013.9
0
H3/H4 ratio
20
-20
H3K9me2
100
0
H3K9me3
100
0
H3K4me2
100
0
RNA-seq
22.9
0

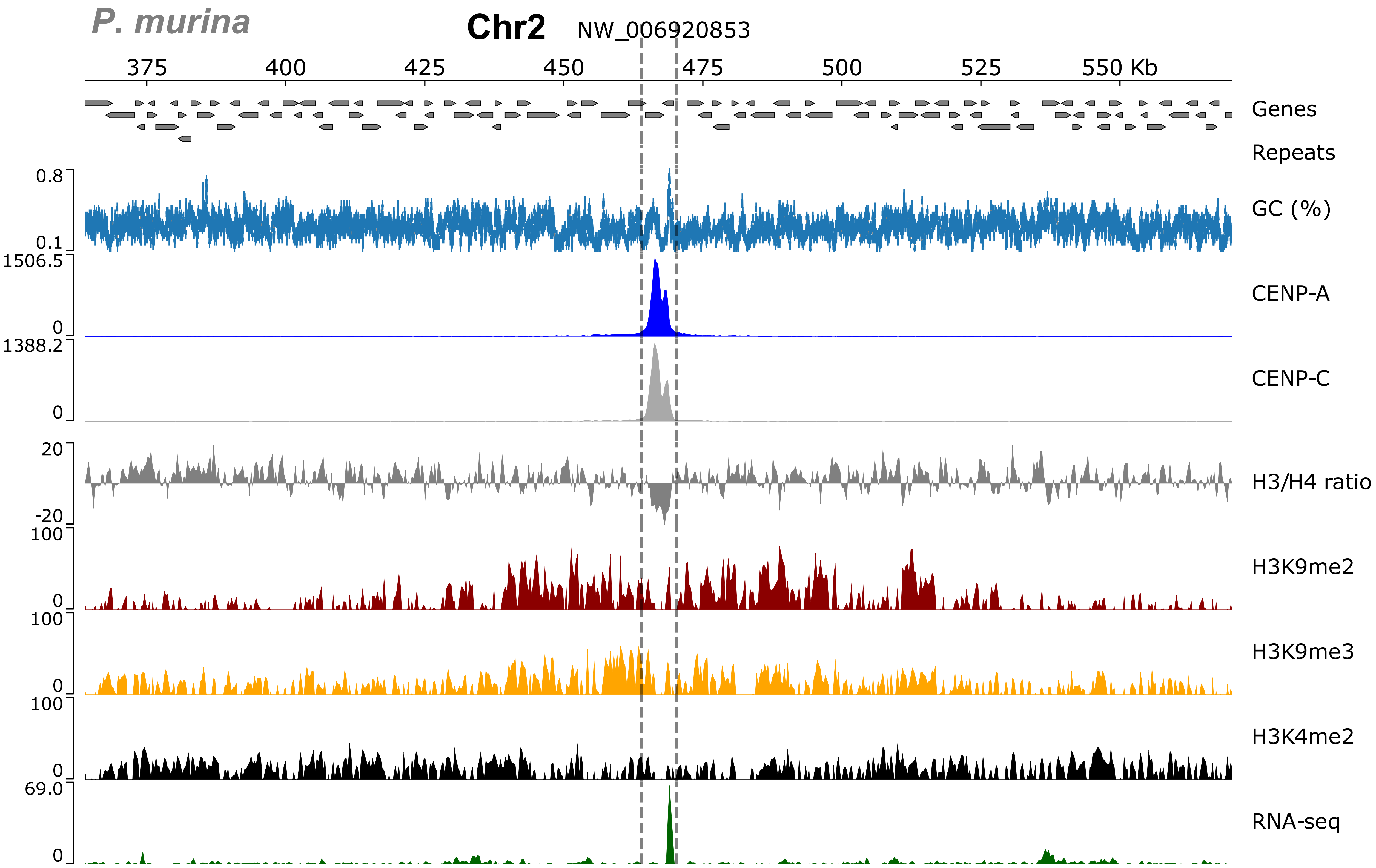

P. murina
Chr2
NW_006920853
375
400
425
450
475
500
525
550 Kb
Genes
Repeats
0.8
0.1
GC (%)
1506.5
0
CENP-A
1388.2
0
CENP-C
20
-20
H3/H4 ratio
100
0
H3K9me2
100
0
H3K9me3
100
0
H3K4me2
69.0
0
RNA-seq

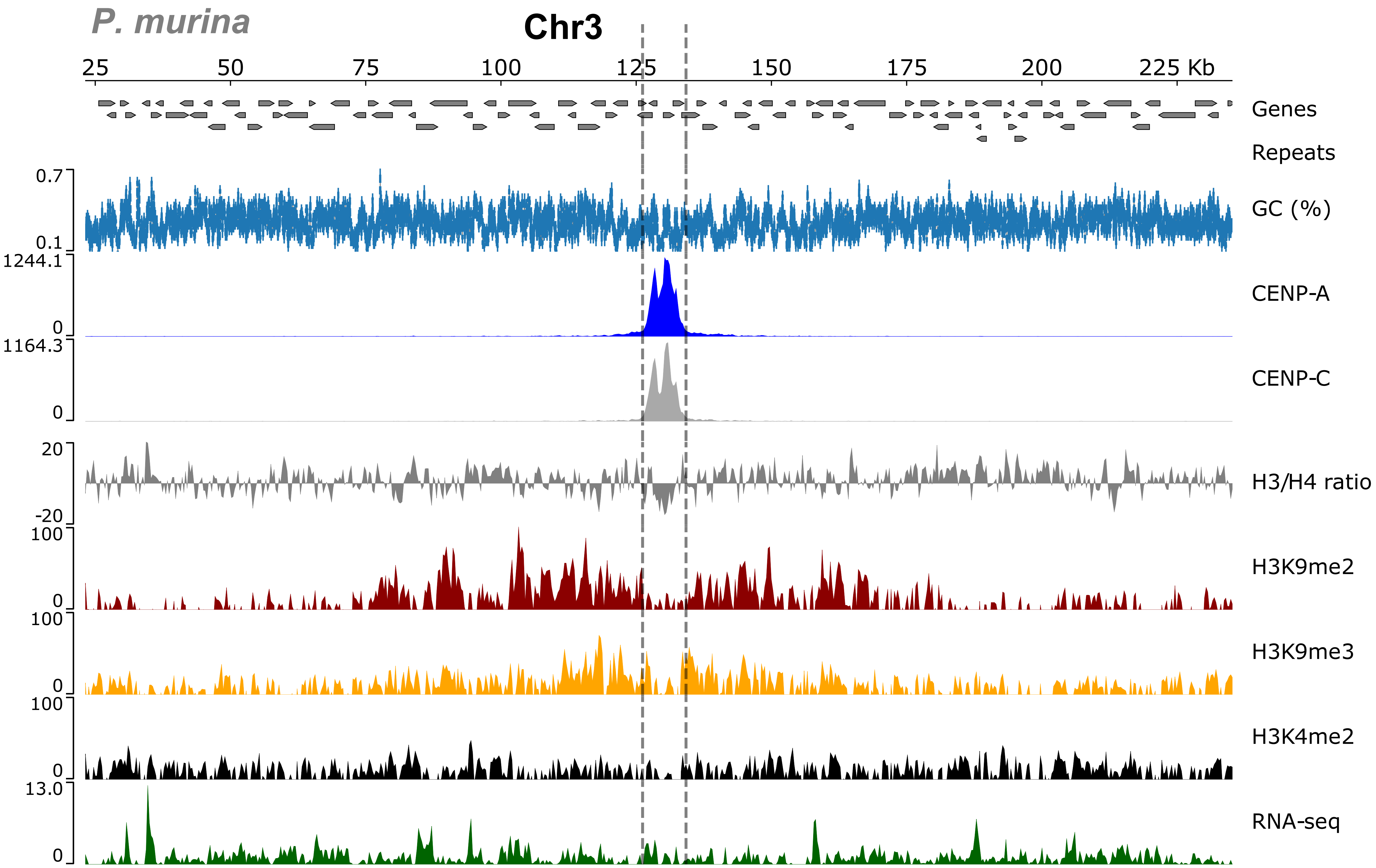

P. murina
Chr3
25
50
75
100
125
150
175
200
225 Kb
Genes
Repeats
0.7
0.1
GC (%)
1244.1
0
CENP-A
1164.3
0
CENP-C
20
-20
H3/H4 ratio
100
0
H3K9me2
100
0
H3K9me3
100
0
H3K4me2
13.0
0
RNA-seq

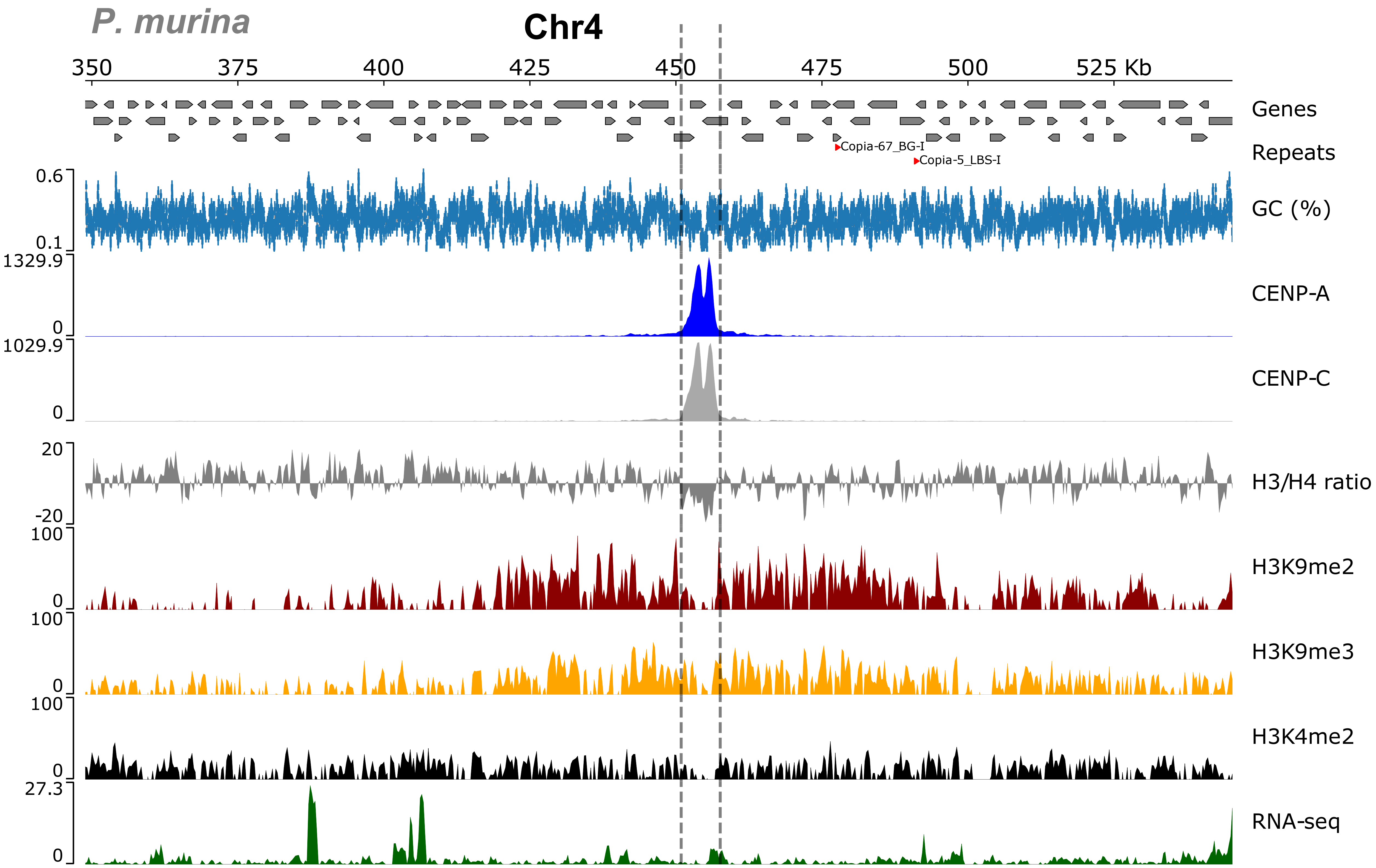

P. murina
Chr4
350
375
400
425
450
475
500
525 Kb
Genes
Repeats
Copia-67_BG-I
Copia-5_LBS-I
0.6
0.1
GC (%)
1329.9
0
CENP-A
1029.9
0
CENP-C
20
-20
H3/H4 ratio
100
0
H3K9me2
100
0
H3K9me3
100
0
H3K4me2
27.3
0
RNA-seq

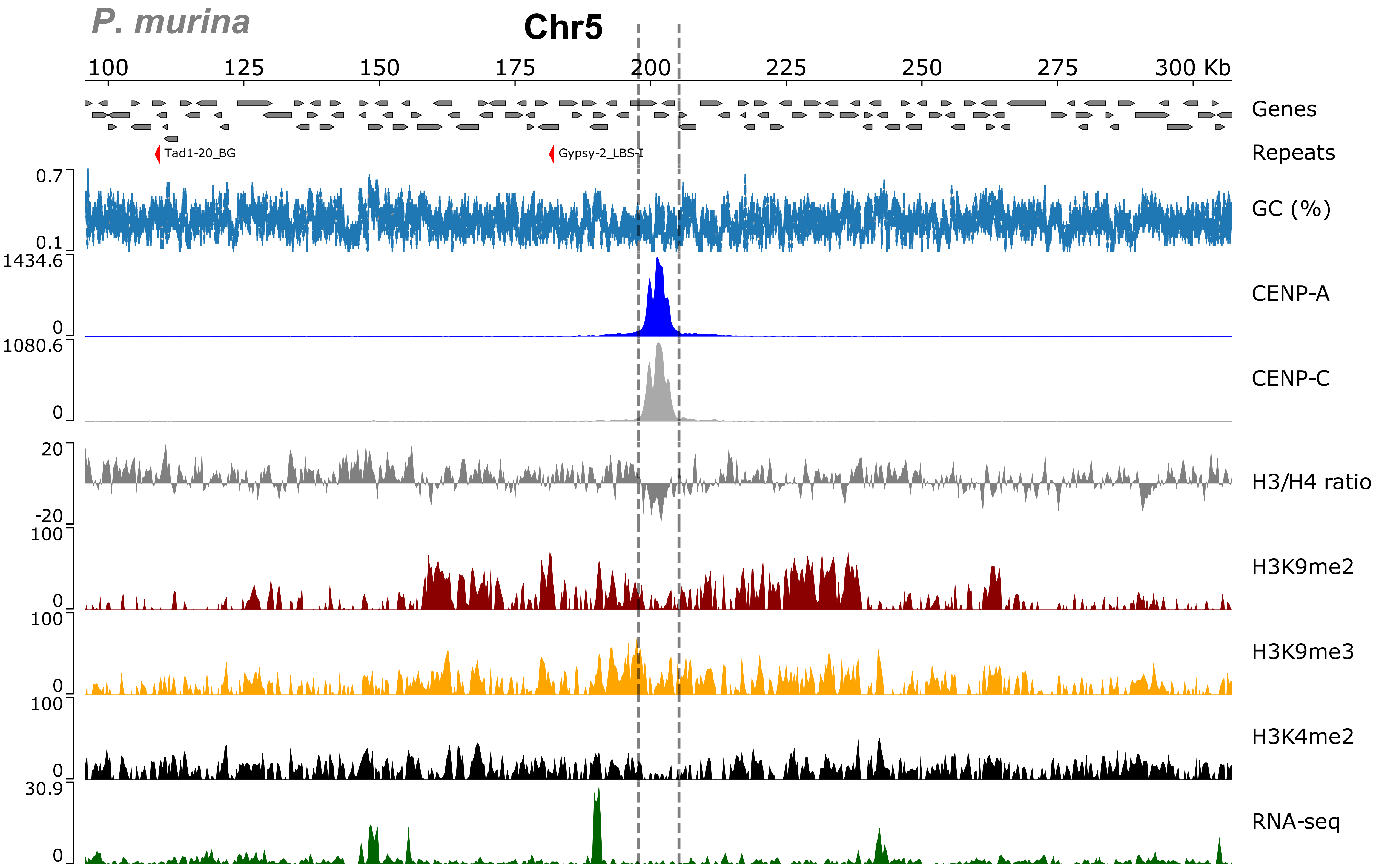

P. murina
Chr5
100
125
150
175
200
225
250
275
300 Kb
Genes
Tad1-20_BG
Gypsy-2_LBS-I
Repeats
0.7
0.1
GC (%)
1434.6
0
CENP-A
1080.6
0
CENP-C
20
-20
H3/H4 ratio
100
0
H3K9me2
100
0
H3K9me3
100
0
H3K4me2
30.9
0
RNA-seq

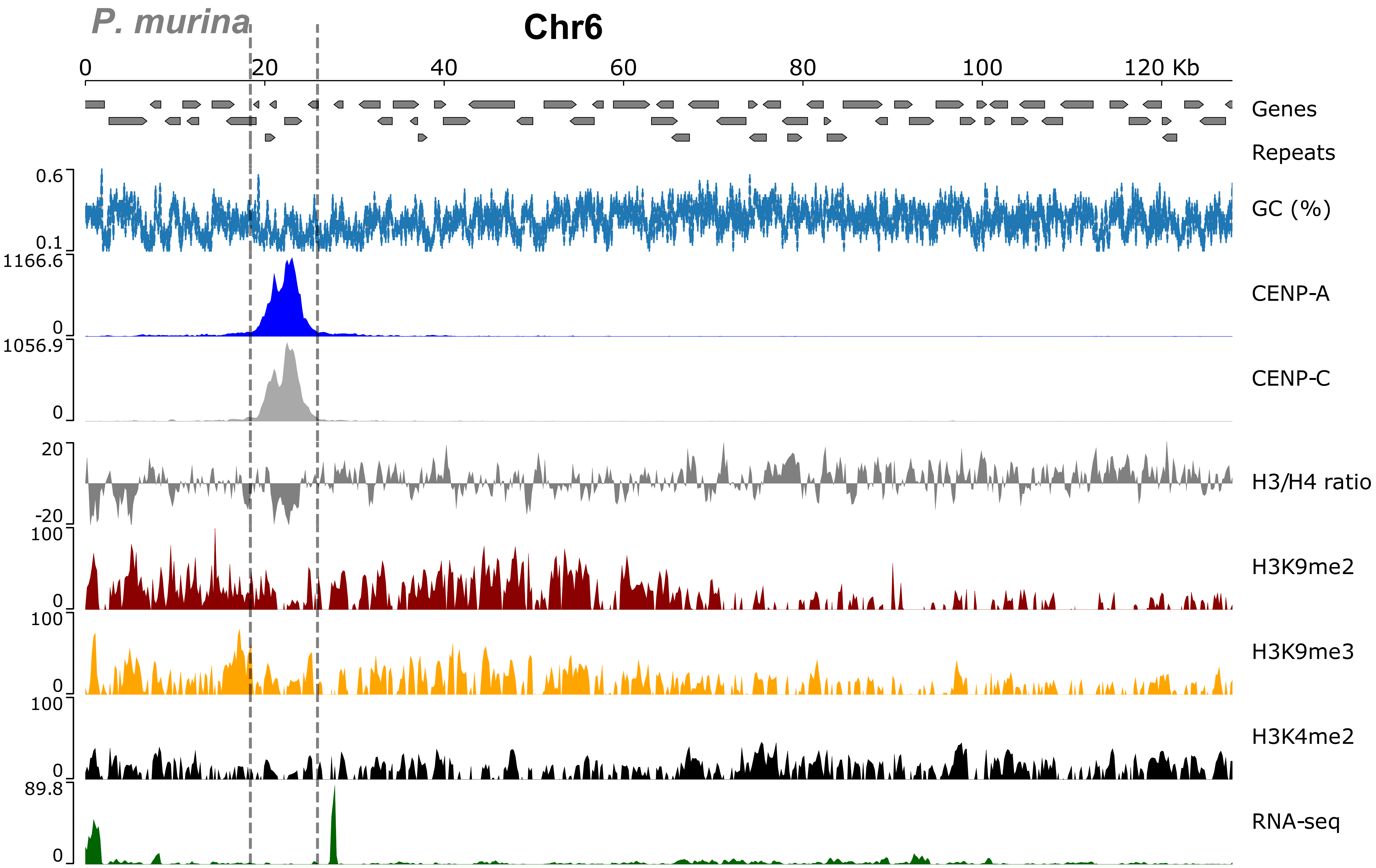

P. murina
Chr6
0
20
40
60
80
100
120 Kb
Genes
Repeats
0.6
0.1
GC (%)
1166.6
0
CENP-A
1056.9
0
CENP-C
20
-20
H3/H4 ratio
100
0
H3K9me2
100
0
H3K9me3
100
0
H3K4me2
89.8
0
RNA-seq

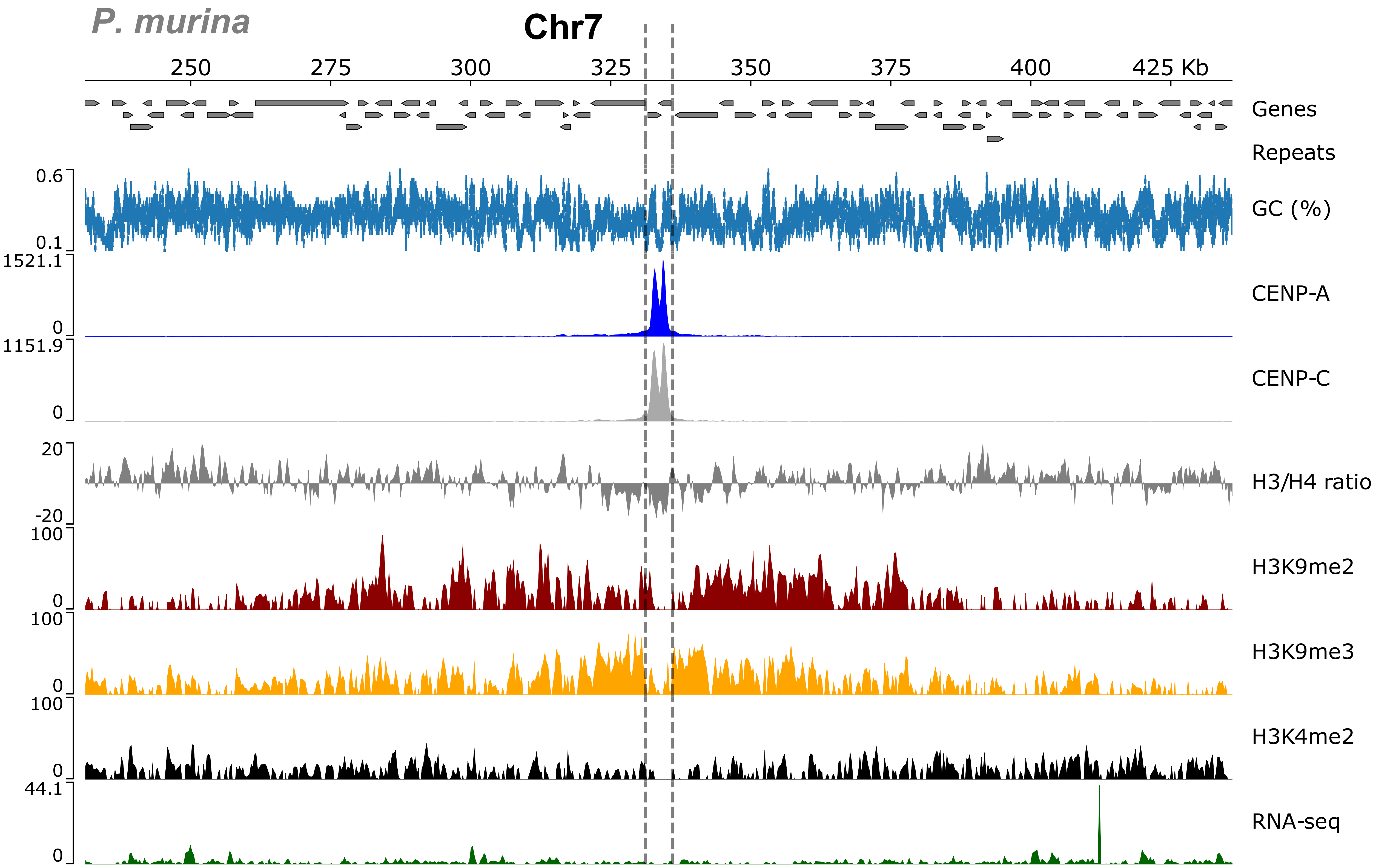

P. murina
Chr7
250
275
300
325
350
375
400
425 Kb
Genes
Repeats
0.6
0.1
GC (%)
1521.1
0
CENP-A
1151.9
0
CENP-C
20
-20
H3/H4 ratio
100
0
H3K9me2
100
0
H3K9me3
100
0
H3K4me2
44.1
0
RNA-seq

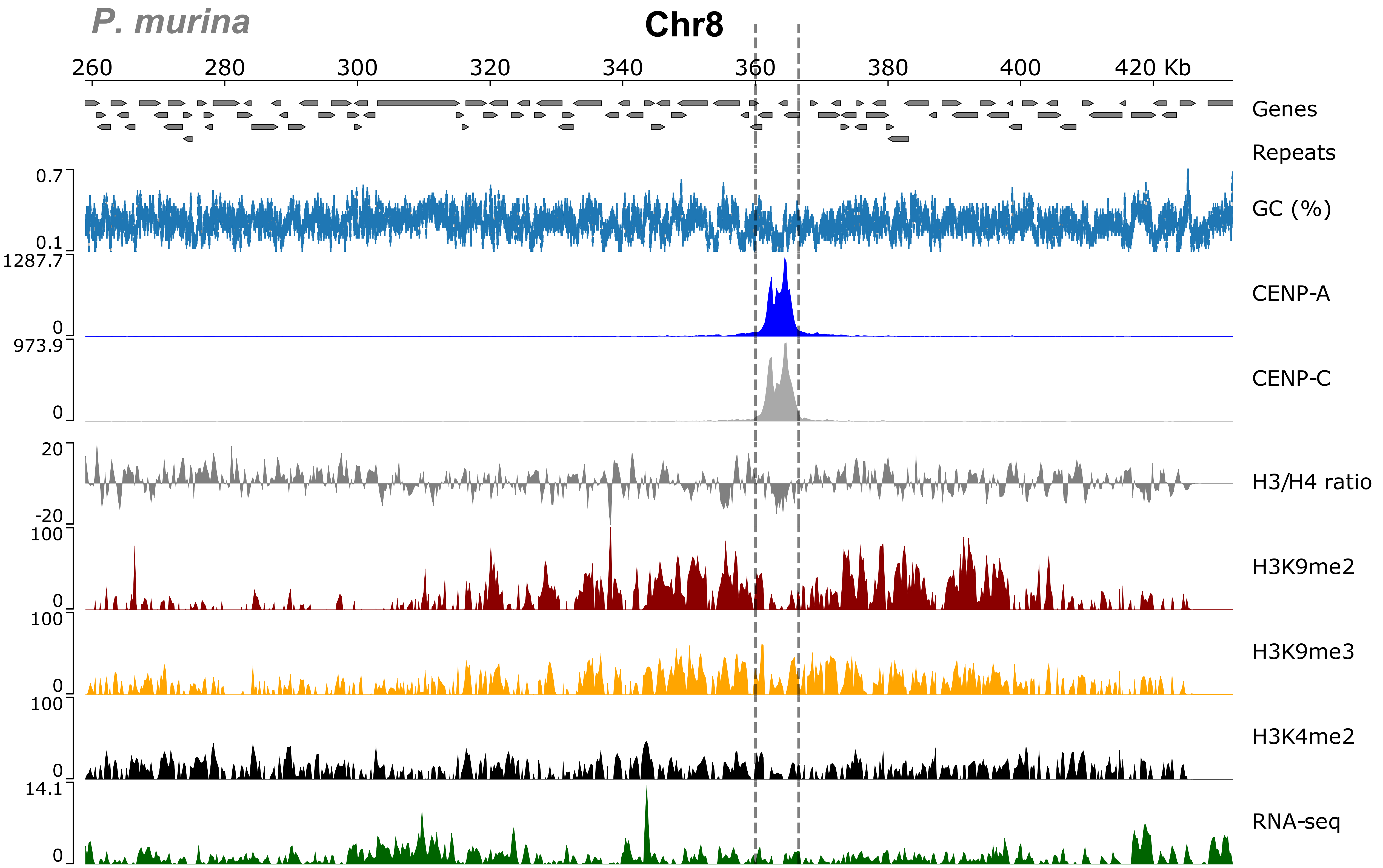

P. murina
Chr8
260
280
300
320
340
360
380
400
420 Kb
Genes
Repeats
0.7
0.1
GC (%)
1287.7
0
CENP-A
973.9
0
CENP-C
20
-20
H3/H4 ratio
100
0
H3K9me2
100
0
H3K9me3
100
0
H3K4me2
14.1
0
RNA-seq

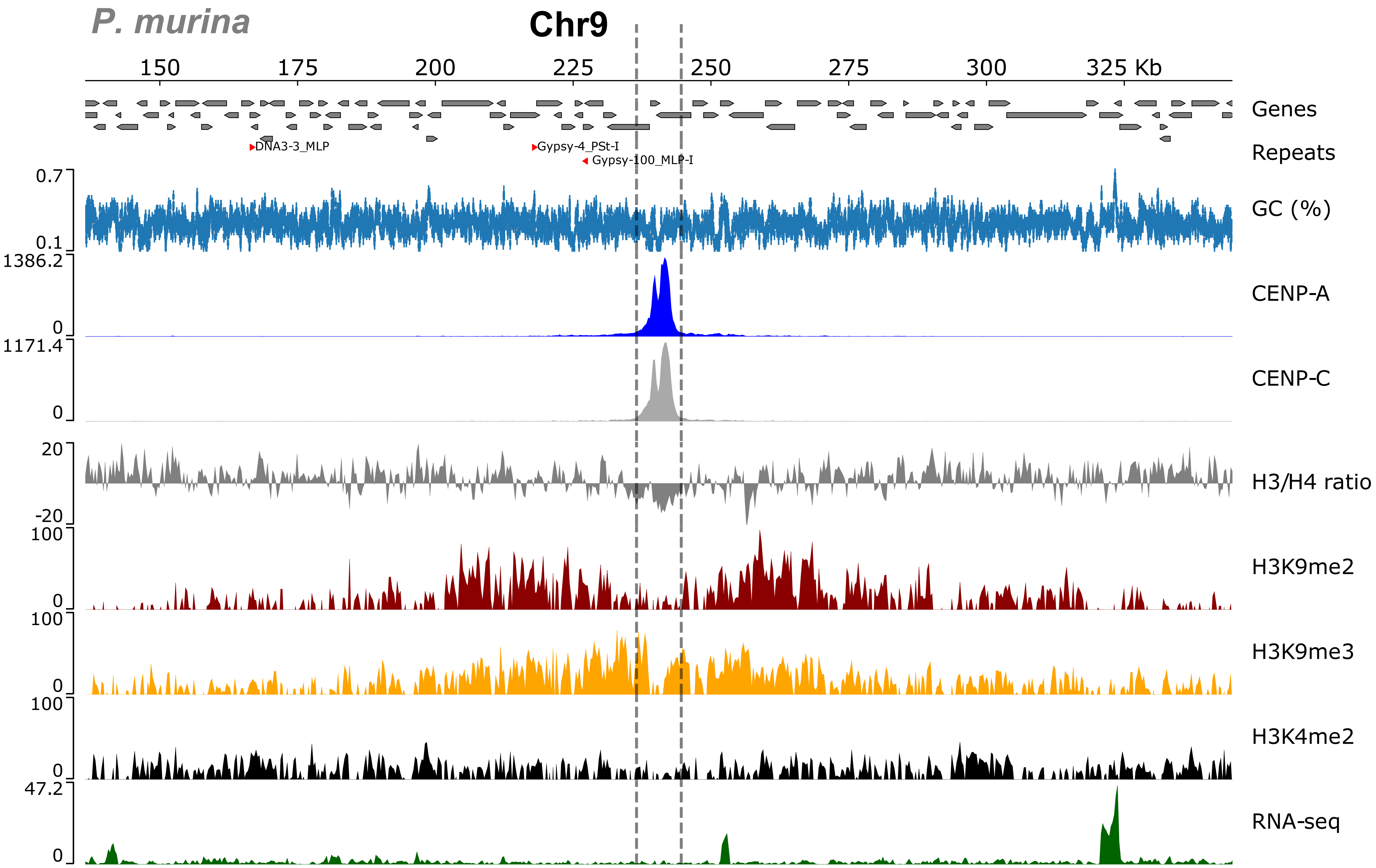
P. murina
Chr9
150
175
200
225
250
275
300
325 Kb
Genes
Repeats
DNA3-3_MLP
Gypsy-4_PSt-I
Gypsy-100_MLP-I
0.7
0.1
GC (%)
1386.2
0
CENP-A
1171.4
0
CENP-C
20
-20
H3/H4 ratio
100
0
H3K9me2
100
0
H3K9me3
100
0
H3K4me2
47.2
0
RNA-seq

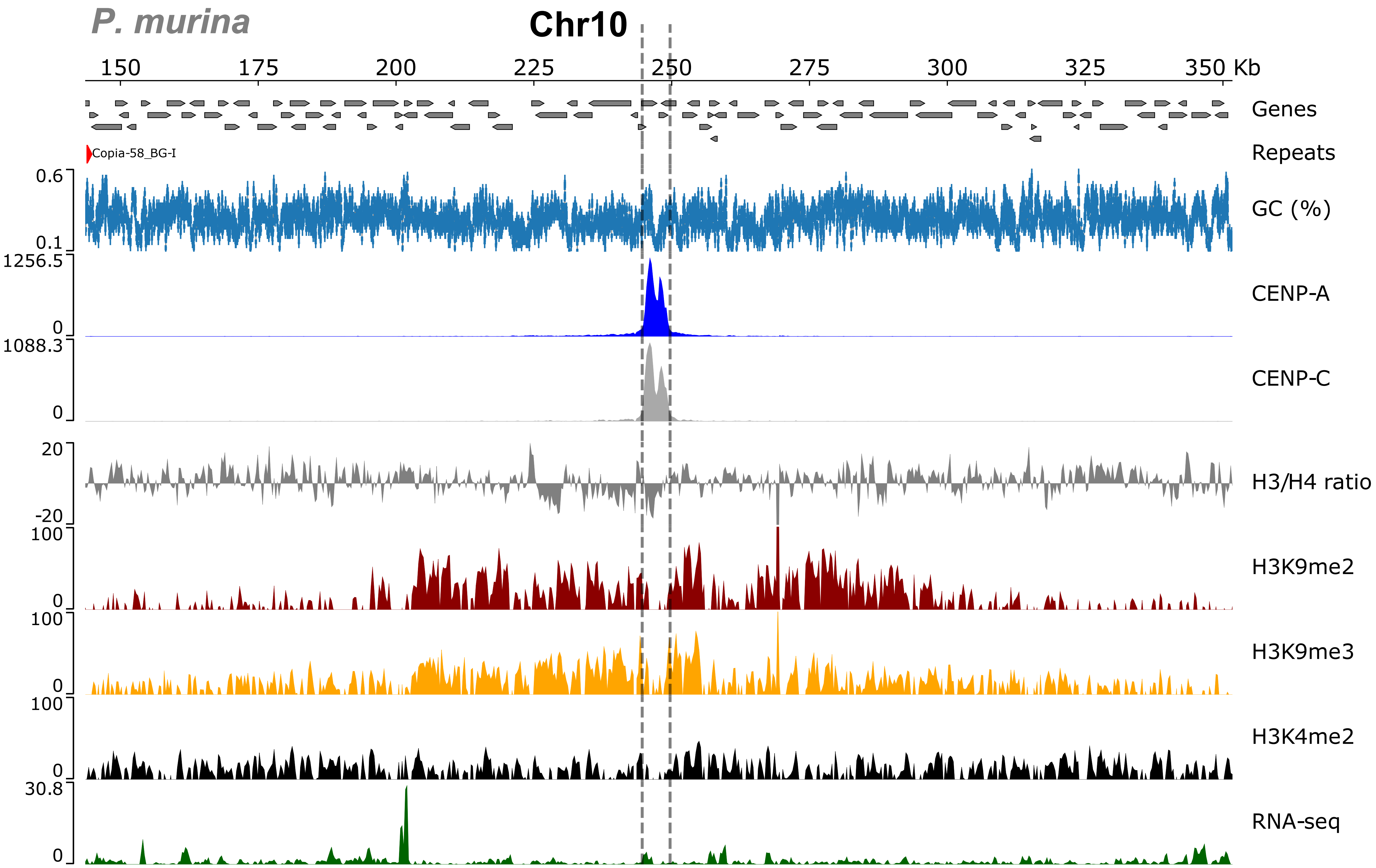

P. murina
Chr10
150
175
200
225
250
275
300
325
350 Kb
Genes
Copia-58_BG-I
Repeats
0.6
0.1
GC (%)
1256.5
0
CENP-A
1088.3
0
CENP-C
20
-20
H3/H4 ratio
100
0
H3K9me2
100
0
H3K9me3
100
0
H3K4me2
30.8
0
RNA-seq

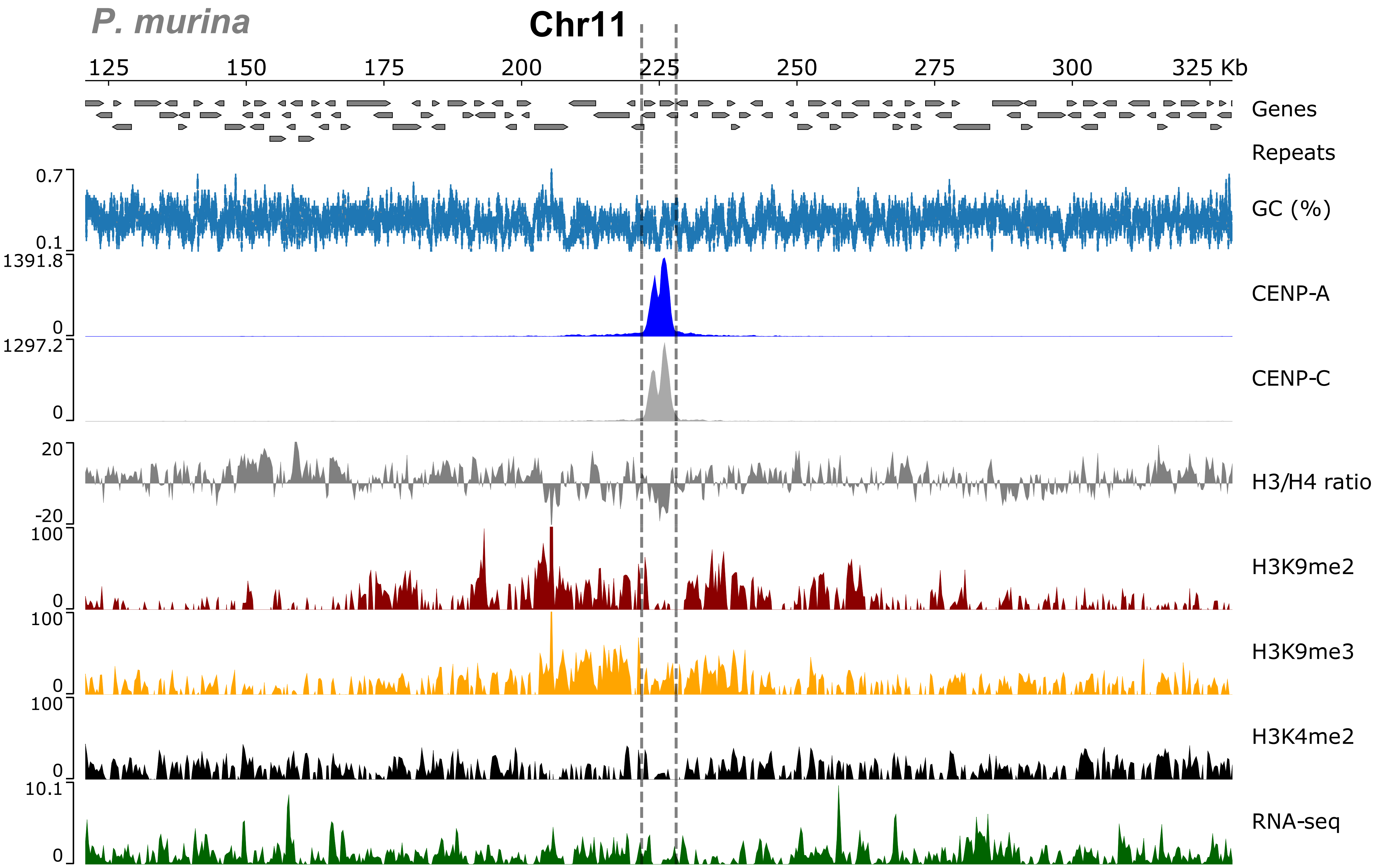
P. murina
Chr11
125
150
175
200
225
250
275
300
325 Kb
Genes
Repeats
0.7
0.1
GC (%)
1391.8
0
CENP-A
1297.2
0
CENP-C
20
-20
H3/H4 ratio
100
0
H3K9me2
100
0
H3K9me3
100
0
H3K4me2
10.1
0
RNA-seq

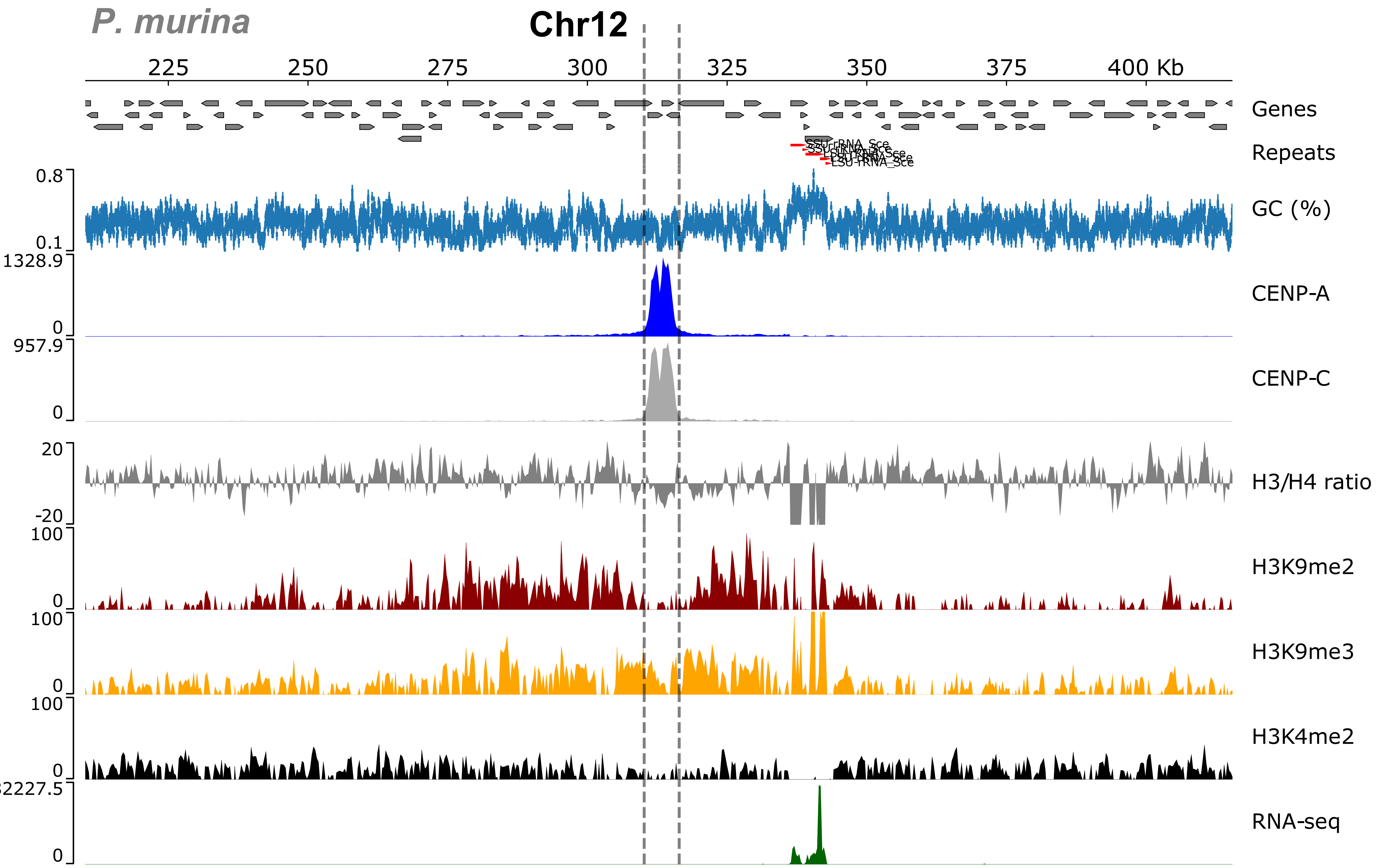
P. murina
Chr12
225
250
275
300
325
350
375
400 Kb
Genes
Repeats
GC (%)
0.8
0.1
CENP-A
1328.9
0
CENP-C
957.9
0
H3/H4 ratio
20
-20
H3K9me2
100
0
H3K9me3
100
0
H3K4me2
100
0
RNA-seq
0

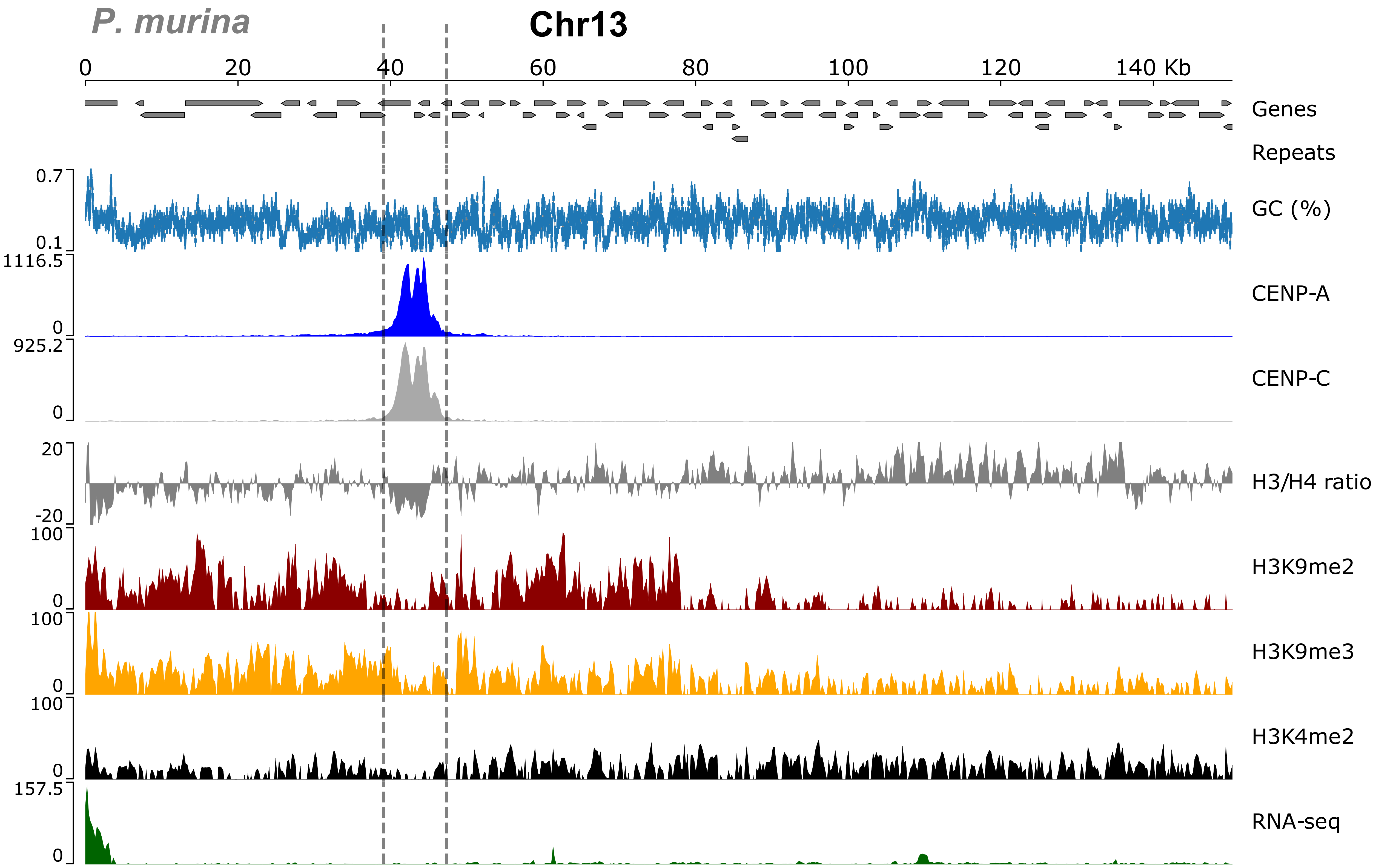
P. murina
Chr13
0
20
40
60
80
100
120
140 Kb
Genes
Repeats
0.7
0.1
GC (%)
1116.5
0
CENP-A
925.2
0
CENP-C
20
-20
H3/H4 ratio
100
0
H3K9me2
100
0
H3K9me3
100
0
H3K4me2
157.5
0
RNA-seq

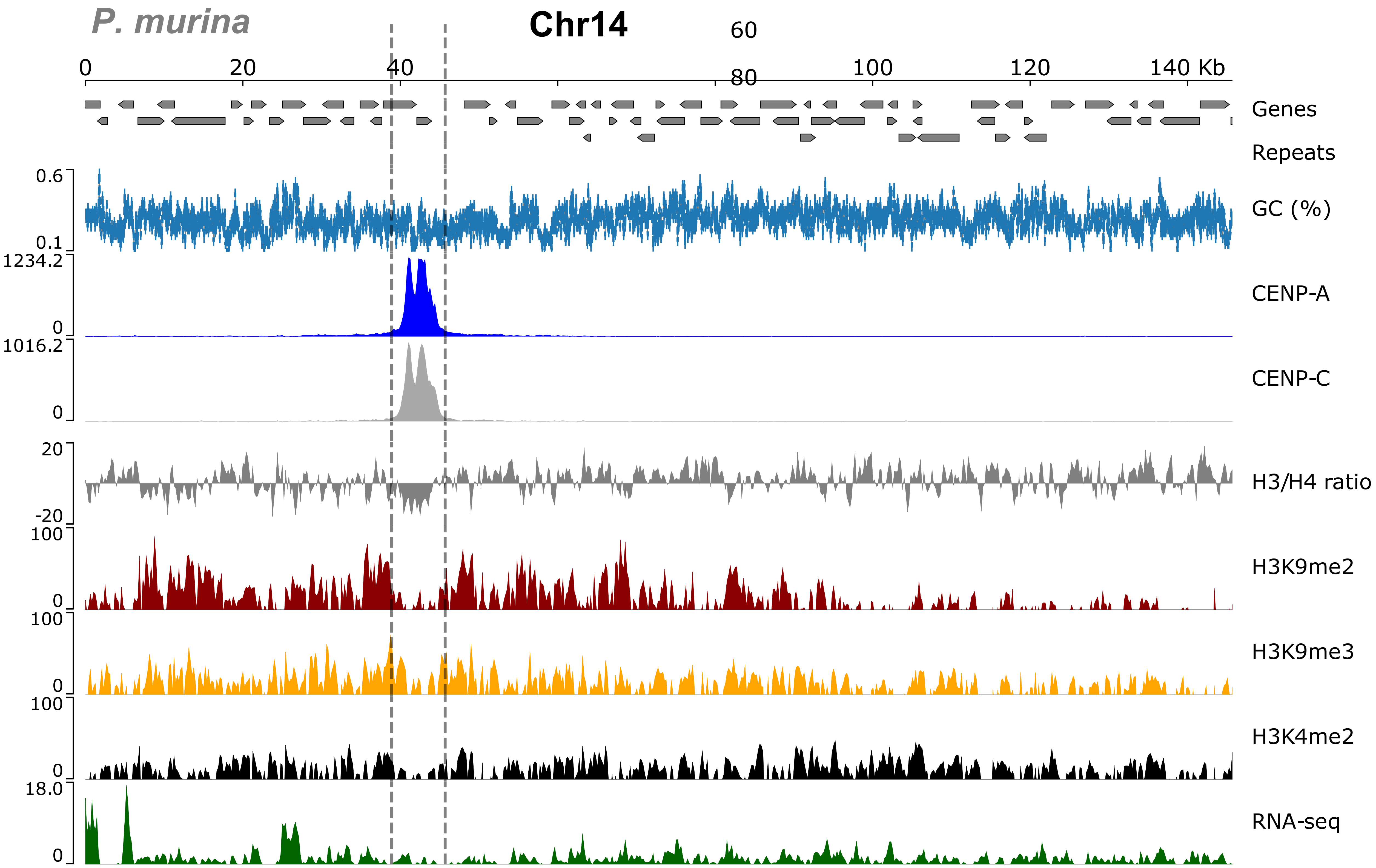

P. murina
Chr14
0
20
40
60
80
100
120
140 Kb
Genes
Repeats
GC (%)
0.6
0.1
CENP-A
1234.2
0
CENP-C
1016.2
0
H3/H4 ratio
20
-20
H3K9me2
100
0
H3K9me3
100
0
H3K4me2
100
0
RNA-seq
18.0
0

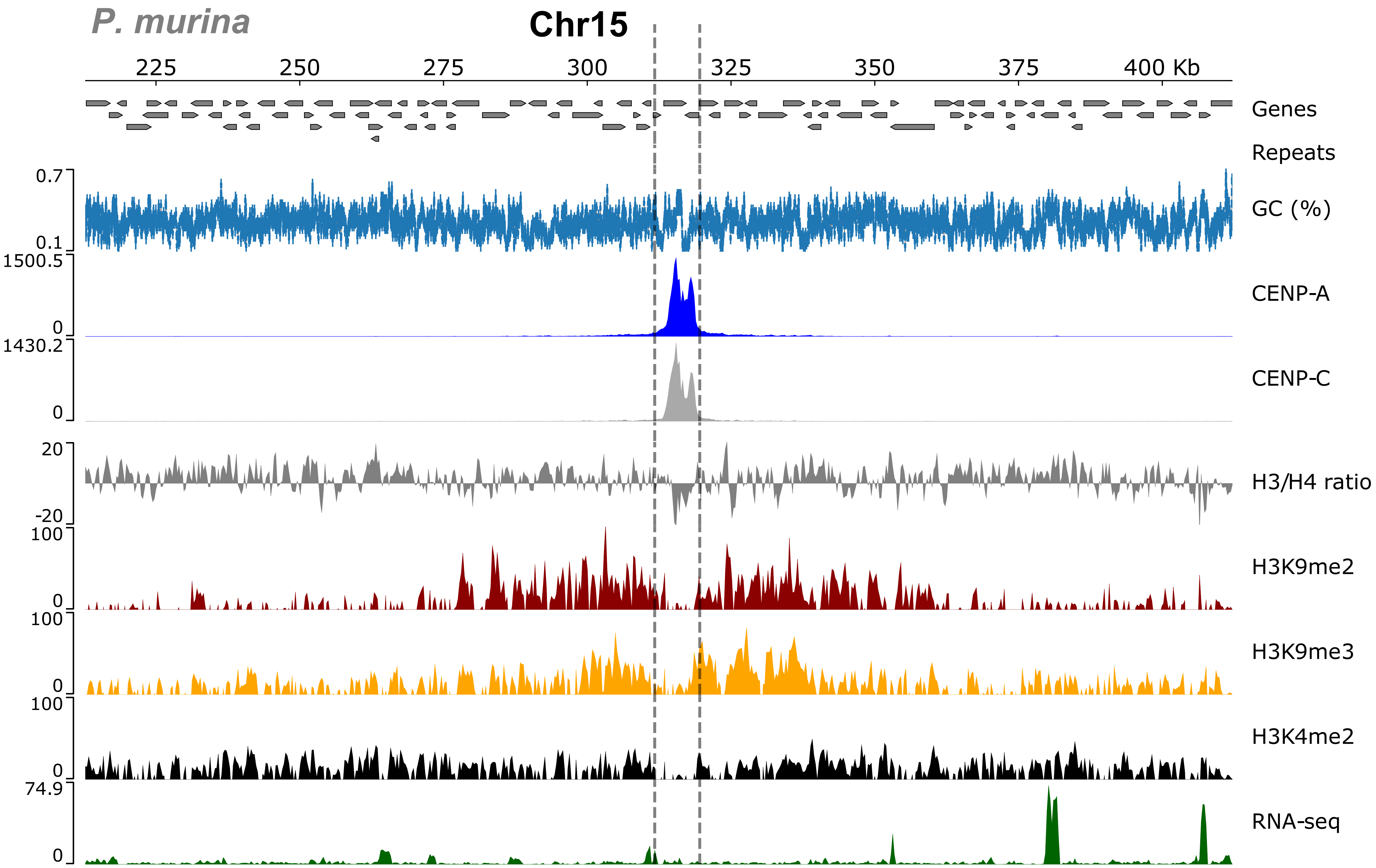

P. murina
Chr15
225
250
275
300
325
350
375
400 Kb
Genes
Repeats
0.7
0.1
GC (%)
1500.5
0
CENP-A
1430.2
0
CENP-C
20
-20
H3/H4 ratio
100
0
H3K9me2
100
0
H3K9me3
100
0
H3K4me2
74.9
0
RNA-seq

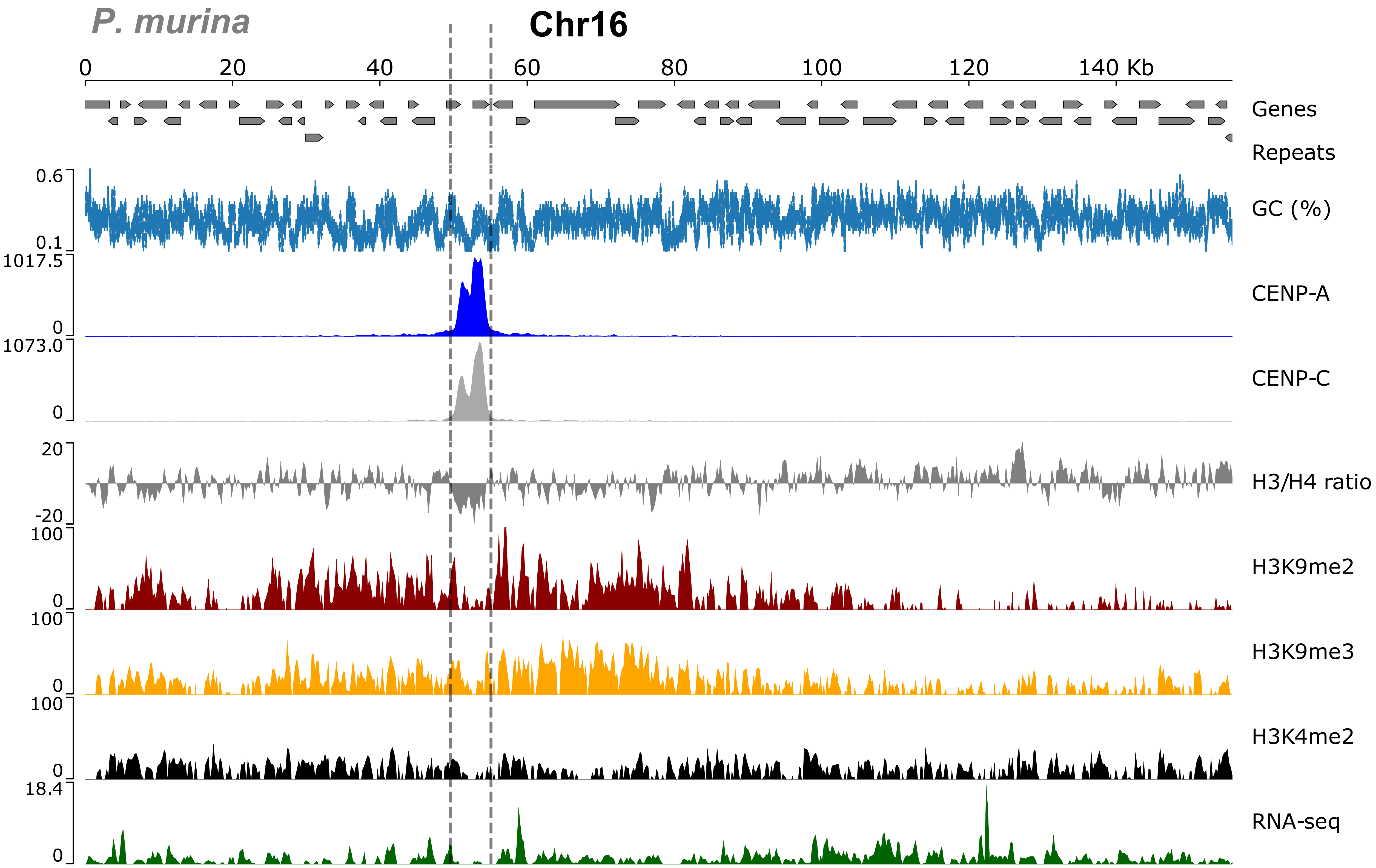

P. murina
Chr16
0
20
40
60
80
100
120
140 Kb
Genes
Repeats
0.6
0.1
GC (%)
1017.5
0
CENP-A
1073.0
0
CENP-C
20
-20
H3/H4 ratio
100
0
H3K9me2
100
0
H3K9me3
100
0
H3K4me2
18.4
0
RNA-seq

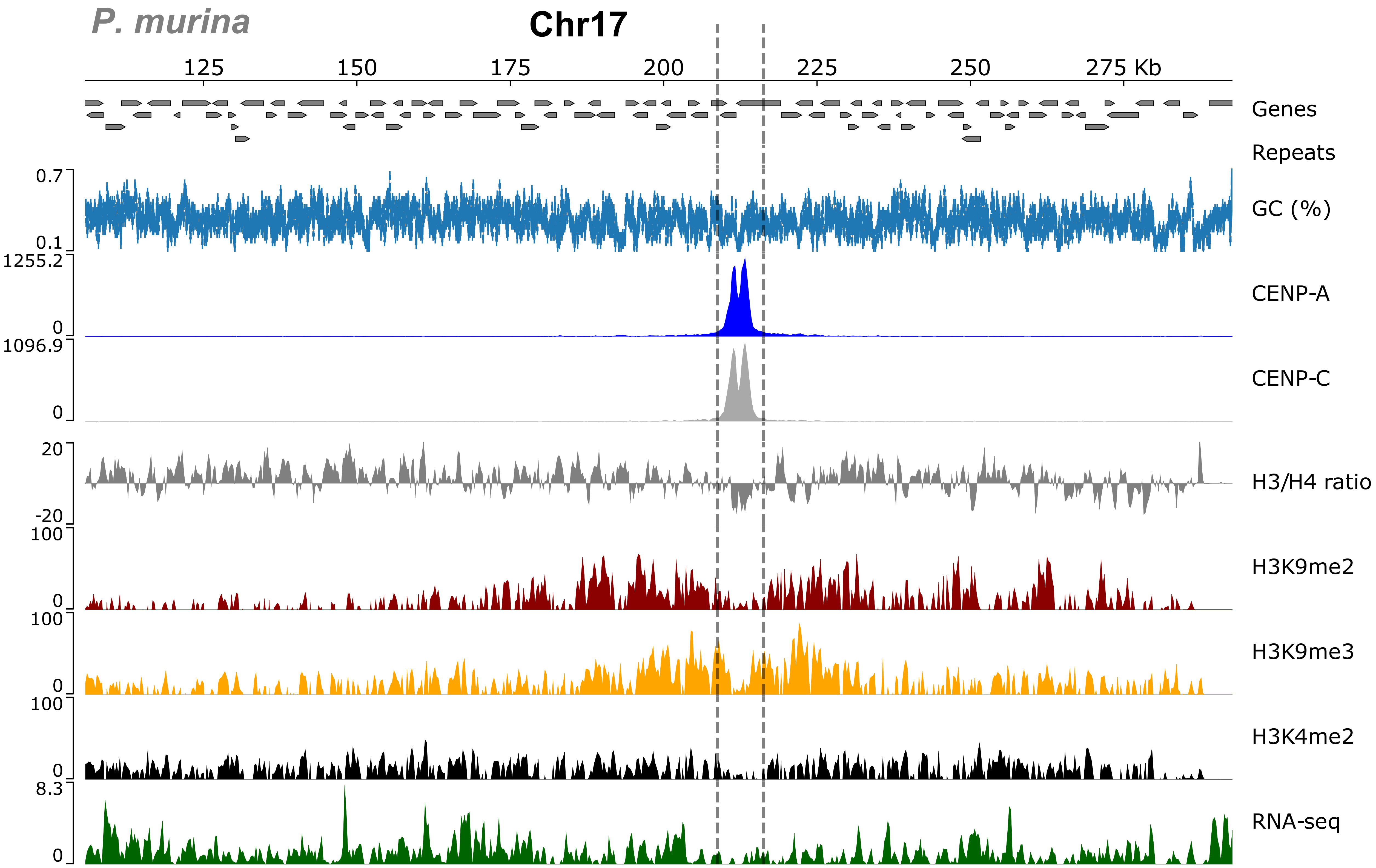
P. murina
Chr17
125
150
175
200
225
250
275 Kb
Genes
Repeats
0.7
0.1
GC (%)
1255.2
0
CENP-A
1096.9
0
CENP-C
20
-20
H3/H4 ratio
100
0
H3K9me2
100
0
H3K9me3
100
0
H3K4me2
8.3
0
RNA-seq

Supplementary figure 8. Centromeres are flanked by heterochromatin and contain active genes.

a) Boxplots of sequence read counts from different ChIP-seq experiments per genomic context summarized over all *P. murina* centromeres (Cens), 30-kilobases regions flanking centromeres (Cens_flank), control regions selected 25-kb away from the centromeres (farCens). ChIP-seq data are presented as the number of reads per bins per millions (BPM) normalized in 50 bp bins with 5-kb windows smoothening and 500-bp slide; *n* represents the numbers of 5-kb windows used for calculations). As ChIP-seq counts are input subtracted (i.e., counts from input DNA are removed), thus negative values indicate that the input DNA (controls) have more coverage than the immunoprecipitated DNA. Statistical differences for the indicated comparisons were obtained using one-sided nonparametric Mann-Whitney test: *P*-values < .0001, ****; $P < .001$, ***; $P < .01$, **; $P < .05$, *; ns, not significant. BPM: number of reads per bin (in millions); TPM, transcripts per million.

b) Boxplots summarizing read counts from different ChIP-seq experiments per genomic context summarized over all *P. carinii* centromeres (Cens), 30-kilobases regions flanking centromeres (Cen_flank), control controls selected 25 kb away from the centromeres for each scaffold (farCens).

c) Boxplots summarizing read counts from different ChIP-seq experiments per genomic context summarized over all *P. macacae* centromeres (Cens), 30-kb regions flanking centromeres (Cens_flank), control regions selected 25-kb away from the centromeres (farCens).

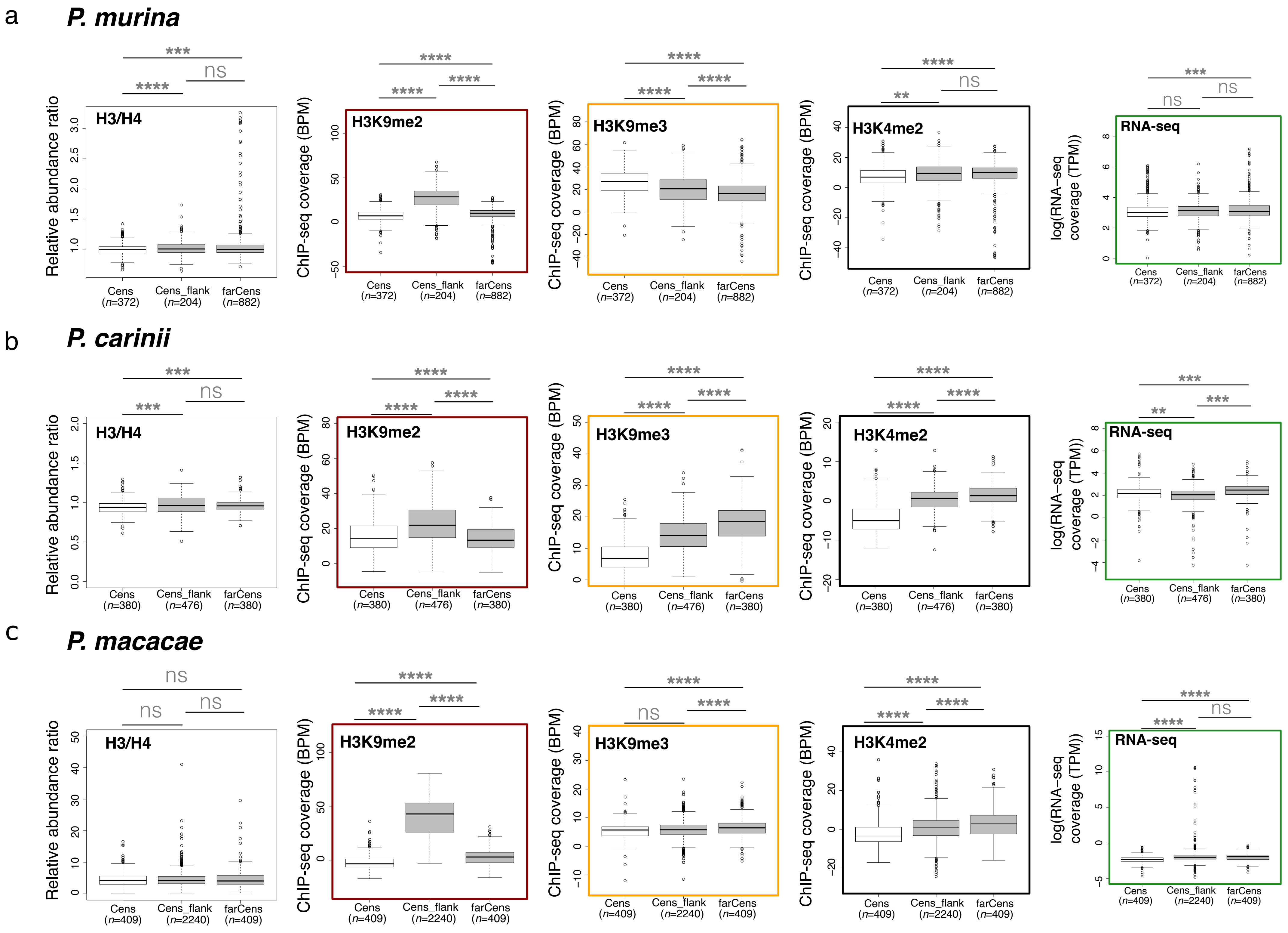
a
P. murina
b
P. carinii
c
P. macacae
H3/H4
H3K9me2
H3K9me3
H3K4me2
RNA-seq
Relative abundance ratio
ChIP-seq coverage (BPM)
log(RNA-seq coverage (TPM))
Cens
Cens_flank
farCens
(n=372)
(n=204)
(n=882)
(n=380)
(n=476)
(n=409)
(n=2240)
ns

Supplementary figure 9. Analysis of canonical histone H3 at *Pneumocystis* centromeres by ChIP-qPCR.

Formalin-fixed chromatins were prepared from *P. murina* and *P. carinii* organisms cultured in coculture of A549 and Let1 cells for 14 days, followed by immunoprecipitation with commercial anti histone H3 and H4 antibodies and DNA extraction. ChIP-qPCR values were determined from three technical replicates. The percent input values are compared to a non-centromeric control region (far-CEN1). Three experiments were performed for reproducibility with the result from a representative experiment presented. Error bars represent the standard errors of the mean. Statistical analyses were performed using one-Way ANOVA and pairwise comparisons were performed using unpaired t-test; ****, $P < 0.0001$; *** $P < 0.001$; ns, not significant). Top, histone H3 fold enrichment is significantly reduced in 15 out 17 centromeres in *P. carinii*. Bottom, H3/H4 relative ratio is significantly reduced in 2 out 17 centromeres in *P. murina*.

a

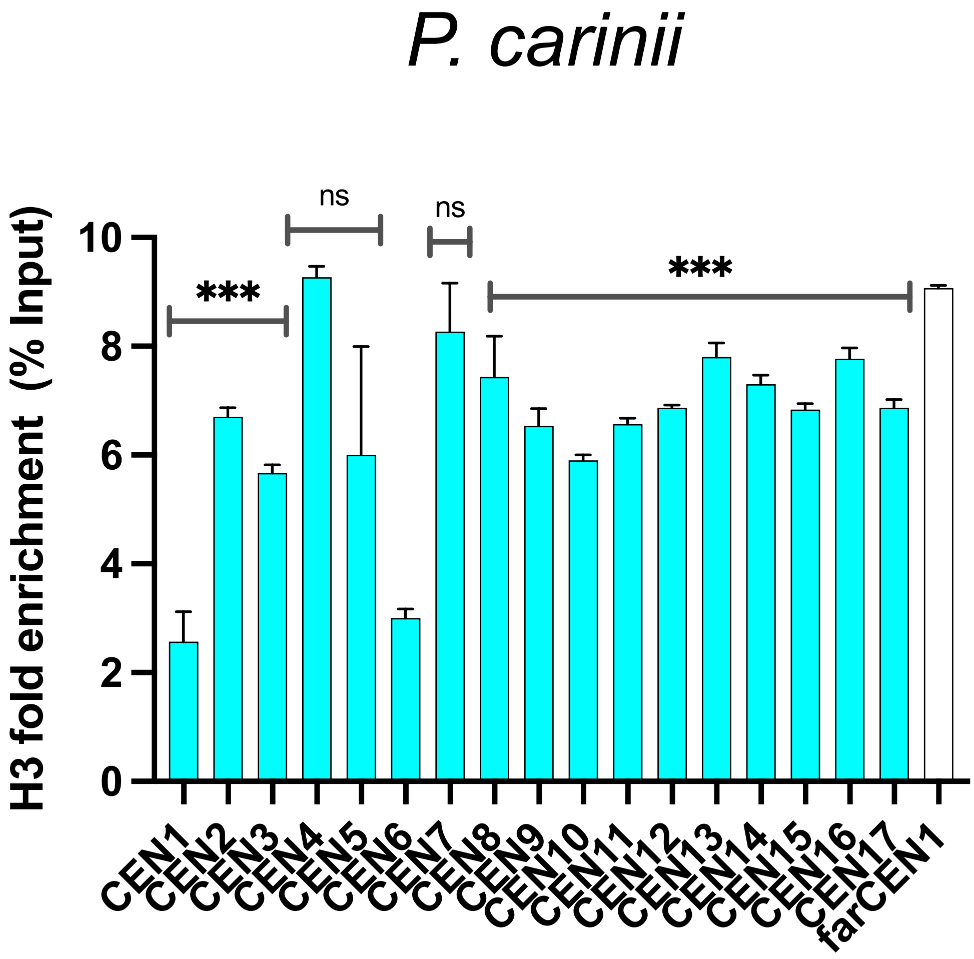
P. carinii
H3 fold enrichment (% Input)
0
2
4
6
8
10
***
ns
ns
***
CEN1
CEN2
CEN3
CEN4
CEN5
CEN6
CEN7
CEN8
CEN9
CEN10
CEN11
CEN12
CEN13
CEN14
CEN15
CEN16
CEN17
farCEN1

b

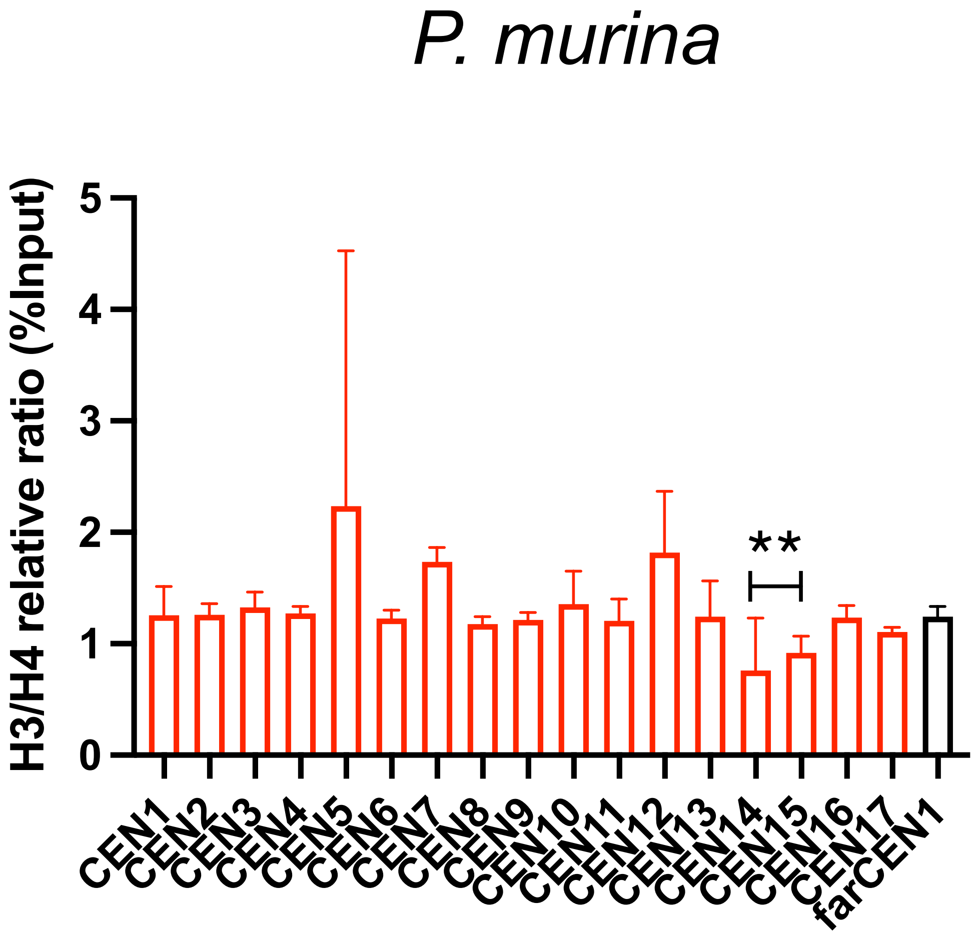
P. murina
H3/H4 relative ratio (%Input)
0
1
2
3
4
5
**
CEN1
CEN2
CEN3
CEN4
CEN5
CEN6
CEN7
CEN8
CEN9
CEN10
CEN11
CEN12
CEN13
CEN14
CEN15
CEN16
CEN17
farCEN1

Supplementary figure 10. 5-methylcytosine (5mC) DNA methylation in *Pneumocystis*.

Top, Summary of DNA methylation in different *Pneumocystis* species as measured by whole genome bisulfite sequencing calculated over 3-kb non overlapping windows. Bottom, boxplots of weighted DNA methylation levels per genomic context (mCG, mCHG, or mCHH) are summarized over genes, intergenic spaces (igs), centromeres and centromeres flanking regions (cen_flank) with a 3-kb genomic window (500-slide). No statistical difference was observed.

Species	Avg. weigthed mCG	Avg. weigthed mCHG	Avg. weigthed mCHH
P.carinii	0.0067	0.0063	0.0065
P.macacae P2C	0.0204	0.0205	0.0216

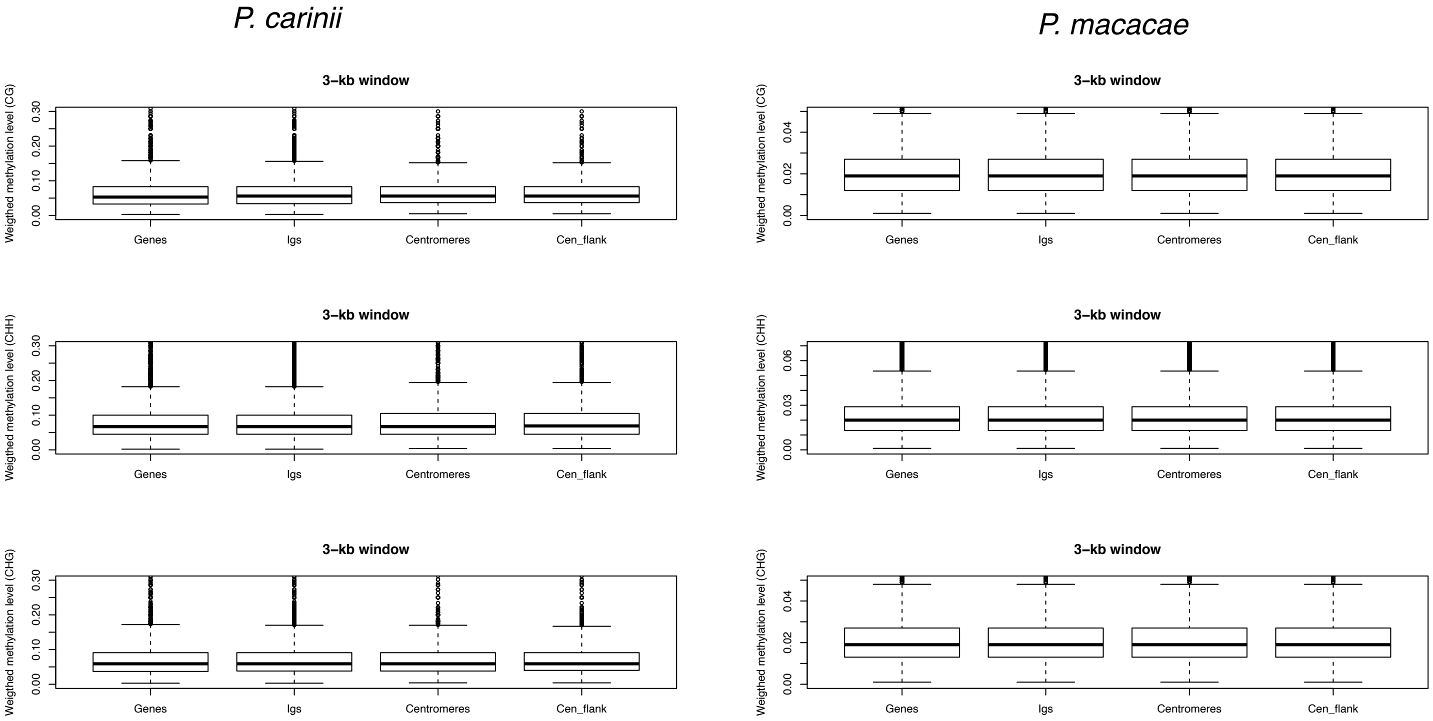

Supplementary figure 11. Genome synteny and centromere locations in *Pneumocystis* species. Circos plots depicting pairwise *P. carinii*, *P. jirovecii* and *P. canis* and *P. oryctolagi* genome synteny. Colored connectors indicate regions of synteny between species. Red links connect *P. carinii* centromeric regions and to their homologous sequences in other species.

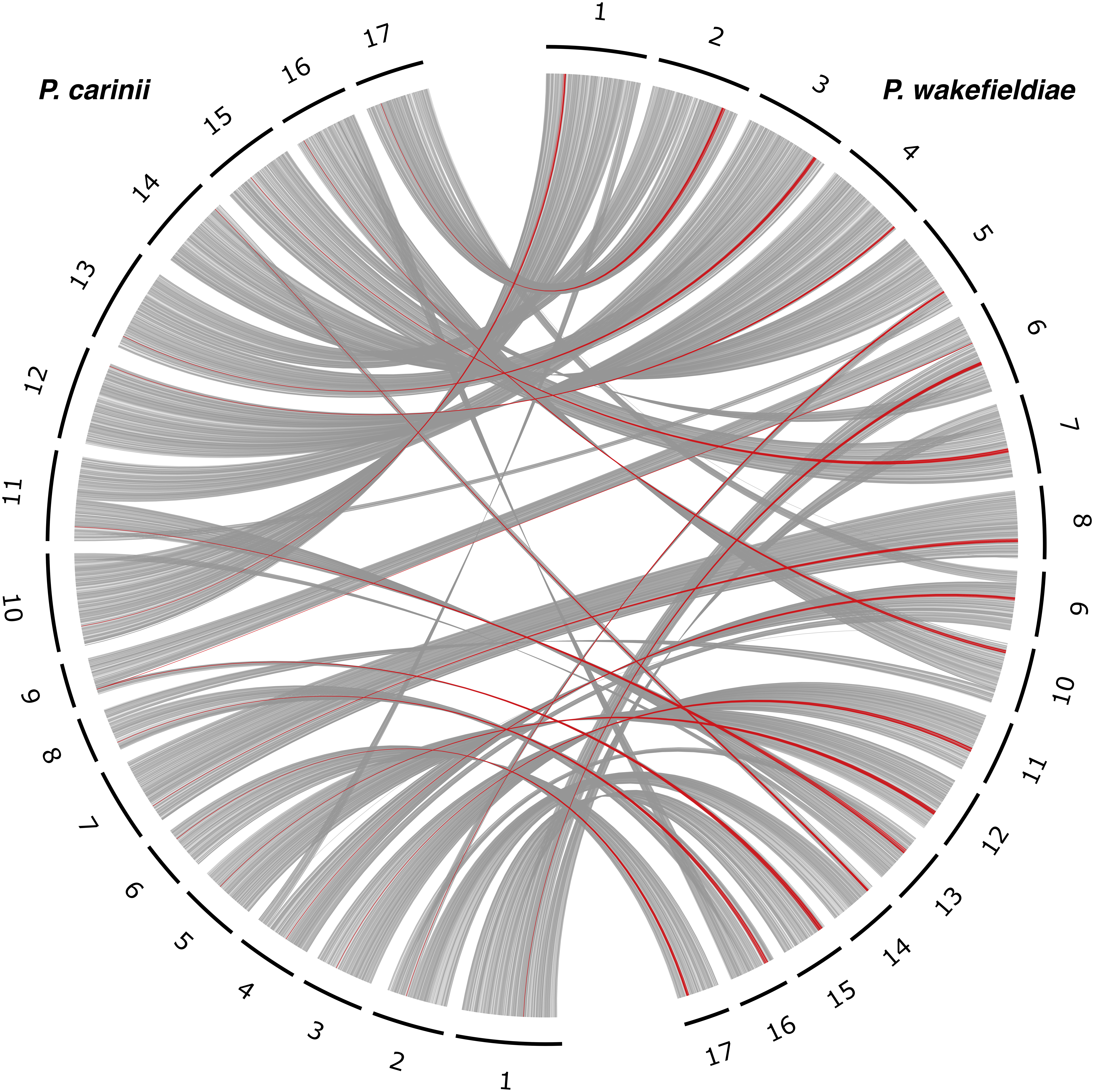
P. carinii
P. wakefieldiae
1
2
3
4
5
6
7
8
9
10
11
12
13
14
15
16
17
1
2
3
4
5
6
7
8
9
10
11
12
13
14
15
16
17

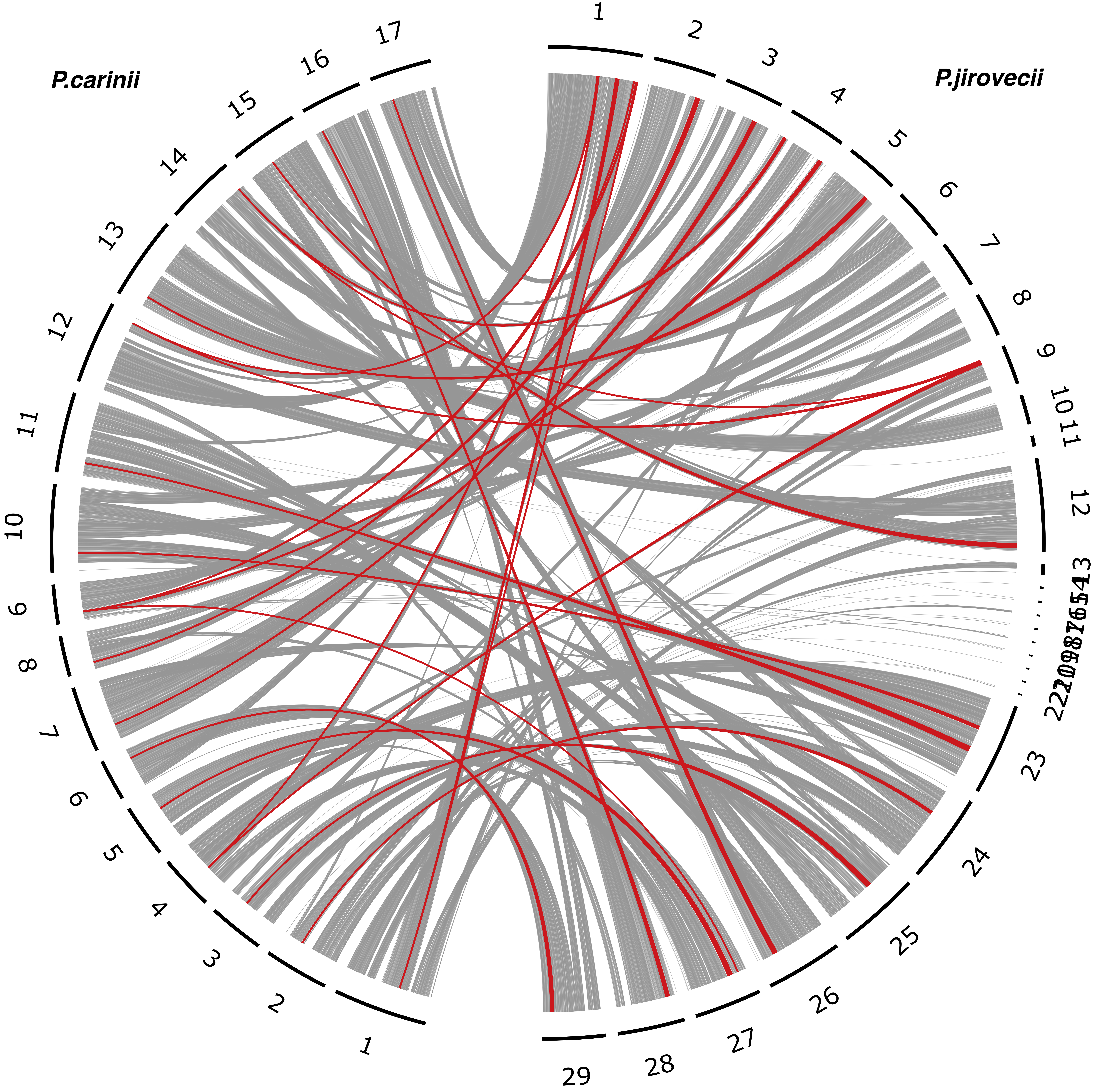

P.carinii
P.jirovecii
1
2
3
4
5
6
7
8
9
10
11
12
13
14
15
16
17
1
2
3
4
5
6
7
8
9
10
11
12
13
23
24
25
26
27
28
29

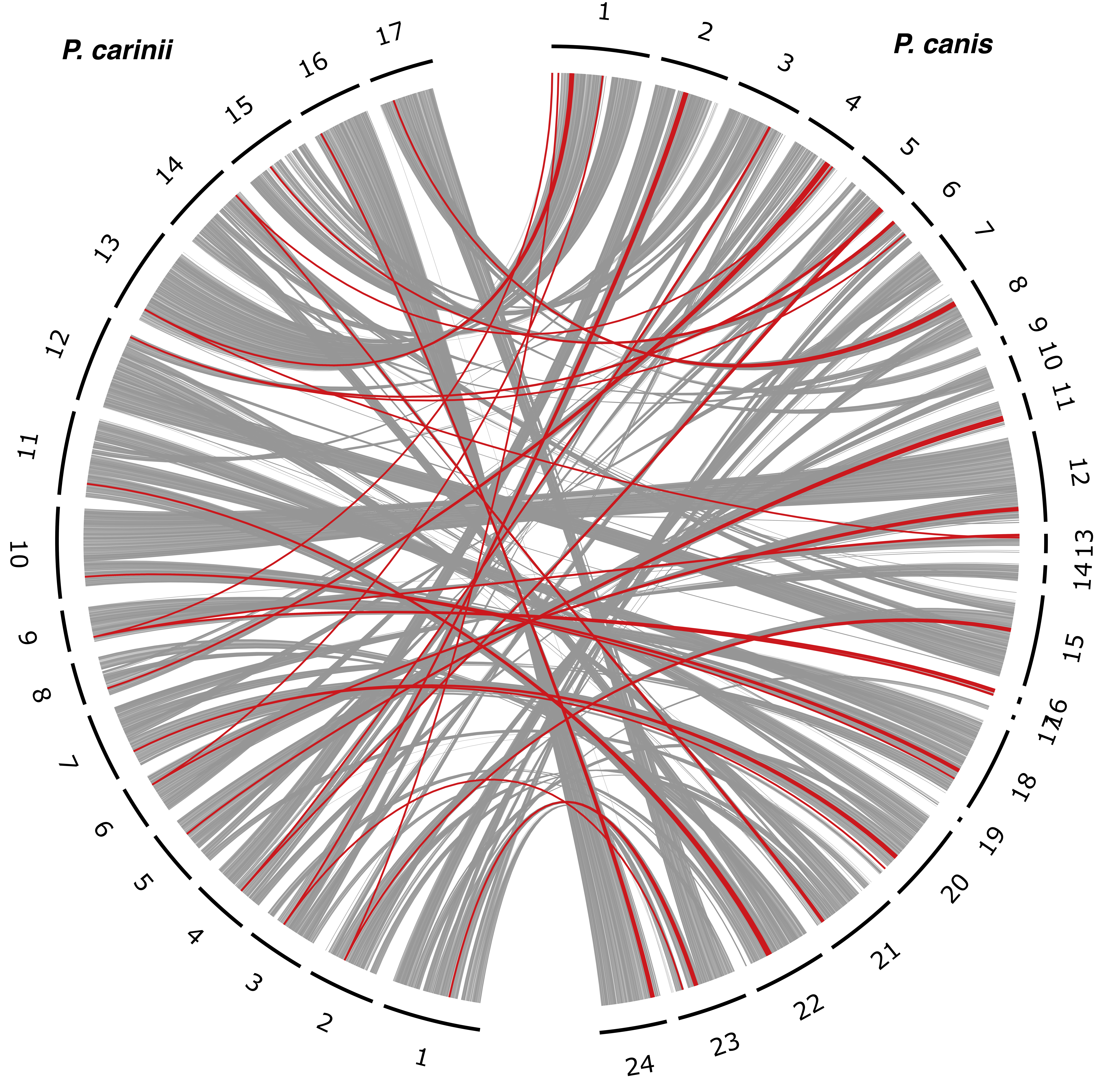

P. carinii
P. canis
17
16
15
14
13
12
11
10
9
8
7
6
5
4
3
2
1
1
2
3
4
5
6
7
8
9
10
11
12
13
14
15
16
17
18
19
20
21
22
23
24

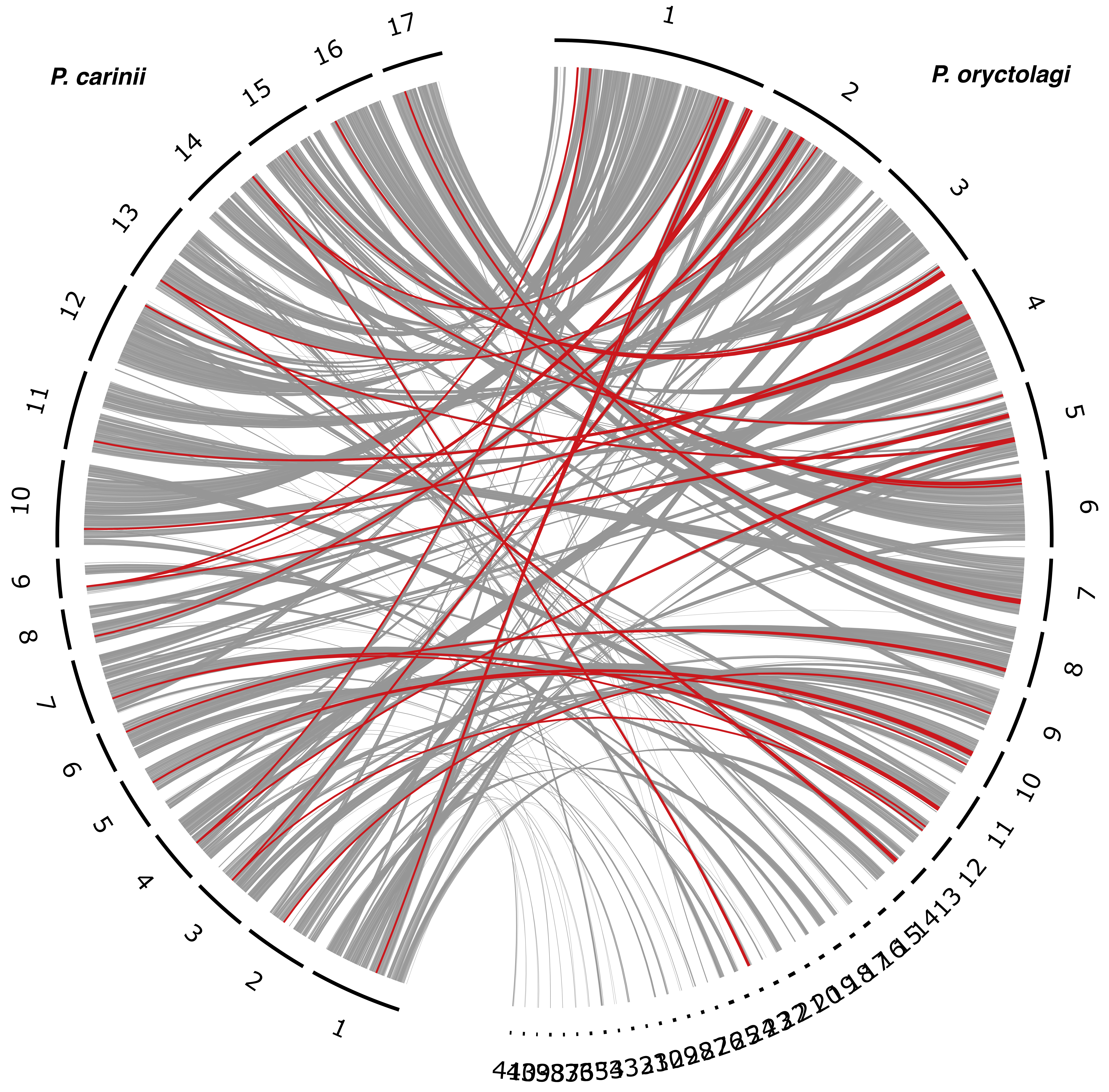
P. carinii
P. oryctolagi
1
2
3
4
5
6
7
8
9
10
11
12
13
14
15
16
17
